# Functional characterization of SGLT1 using SSM-based electrophysiology: Kinetics of sugar binding and translocation

**DOI:** 10.3389/fphys.2023.1058583

**Published:** 2023-02-07

**Authors:** Andre Bazzone, Rocco Zerlotti, Maria Barthmes, Niels Fertig

**Affiliations:** ^1^ Nanion Technologies GmbH, Munich, Germany; ^2^ Department of Structural Biology, Faculty of Biology and Pre-Clinics, Institute of Biochemistry, Genetics and Microbiology, University of Regensburg, Regensburg, Germany

**Keywords:** SGLT1, pre-steady-state kinetics, transport mechanism, solid supported membrane-based electrophysiology, SLC transporters, binding assay, kinetics, membrane transporter

## Abstract

Beside the ongoing efforts to determine structural information, detailed functional studies on transporters are essential to entirely understand the underlying transport mechanisms. We recently found that solid supported membrane-based electrophysiology (SSME) enables the measurement of both sugar binding and transport in the Na^+^/sugar cotransporter SGLT1 (Bazzone et al, 2022a). Here, we continued with a detailed kinetic characterization of SGLT1 using SSME, determining K_M_ and K_D_^app^ for different sugars, k_obs_ values for sugar-induced conformational transitions and the effects of Na^+^, Li^+^, H^+^ and Cl^−^ on sugar binding and transport. We found that the sugar-induced pre-steady-state (PSS) charge translocation varies with the bound ion (Na^+^, Li^+^, H^+^ or Cl^−^), but not with the sugar species, indicating that the conformational state upon sugar binding depends on the ion. Rate constants for the sugar-induced conformational transitions upon binding to the Na^+^-bound carrier range from 208 s^−1^ for D-glucose to 95 s^−1^ for 3-OMG. In the absence of Na^+^, rate constants are decreased, but all sugars bind to the empty carrier. From the steady-state transport current, we found a sequence for sugar specificity (V_max_/K_M_): D-glucose > MDG > D-galactose > 3-OMG > D-xylose. While K_M_ differs 160-fold across tested substrates and plays a major role in substrate specificity, V_max_ only varies by a factor of 1.9. Interestingly, D-glucose has the lowest V_max_ across all tested substrates, indicating a rate limiting step in the sugar translocation pathway following the fast sugar-induced electrogenic conformational transition. SGLT1 specificity for D-glucose is achieved by optimizing two ratios: the sugar affinity of the empty carrier for D-glucose is similarly low as for all tested sugars (K_D,K_^app^ = 210 mM). Affinity for D-glucose increases 14-fold (K_D,Na_^app^ = 15 mM) in the presence of sodium as a result of cooperativity. Apparent affinity for D-glucose during transport increases 8-fold (K_M_ = 1.9 mM) compared to K_D,Na_^app^ due to optimized kinetics. In contrast, K_M_ and K_D_^app^ values for 3-OMG and D-xylose are of similar magnitude. Based on our findings we propose an 11-state kinetic model, introducing a random binding order and intermediate states corresponding to the electrogenic transitions detected *via* SSME upon substrate binding.

## 1 Introduction

The sodium glucose cotransporter SGLT1 represents an extensively studied model transporter within the Solute Sodium Symporter (SSS) family. The SSS family belongs to the Amino Acid-Polyamine-Organocation (APC) superfamily of transporters which is the second largest superfamily of secondary active transporters. Transporters of the SSS family facilitate the transport of diverse solutes against their concentration gradients *via* coupling to an electrochemical sodium gradient. SGLT1 adopts the inverted repeat fold of the LeuT structural family (Lolkema and Slotboom, 2008) and is widely expressed in the small intestine and the distal segment of the proximal tubule, where it plays an important role in the adsorption of glucose (Ghezzi et al., 2018). Due to its central role in energy metabolism, SGLTs have been described as potential targets for the treatment of *diabetes mellitus* and cancer (Tsujihara et al., 1996; Koepsell, 2017).

Secondary active transport is often enabled by an alternating access mechanism (Mitchell, 1957; Jardetzky, 1966). For transporters with the characteristic LeuT fold, a rocking bundle mechanism was proposed (Forrest et al., 2008; Weyand et al., 2008; Forrest and Rudnick, 2009; Kazmier et al., 2017). It considers a rocking motion of concerted helices to be sufficient to allow alternating access to the respective ligand binding site from the extracellular and the intracellular environment. Related theories sought to explain the coupled solute transport with an internal symmetry of the protein (Karpowich and Wang, 2008; Drew and Boudker, 2016). While the rocking bundle mechanism can explain the primary conformational change in SGLT1, it does not distinguish between the binding and release of the driving ions and the substrate, which is mandatory to accurately capture the structural framework of the transport cycle. A kinetic model for SGLT1 includes the opening and closure of an intracellular and an extracellular gate (Sala-Rabanal et al., 2012; Ghezzi et al., 2018), also visible in the recently solved SGLT1 structure (Han et al., 2022). These smaller conformational changes allow for selective ligand binding, which causes subsequent major structural changes.

A commonly proposed molecular transport mechanism starts with sodium binding to an extracellular side of SGLT1 (Parent et al., 1992b; Wright et al., 2011). This triggers the opening of the extracellular gate allowing for subsequent sugar binding which then causes the closure of the extracellular gate and the rocking bundle movement to the inward facing transporter conformation. This is followed by the opening of the intracellular gate and subsequent sodium and sugar release. Since Na^+^/sugar symport transfers a net charge across the plasma membrane, it allows for electrophysiological measurements on SGLT1. Previously performed voltage clamp measurements revealed details about its steady-state kinetics. Voltage steps also revealed pre-steady-state (PSS) currents, which have been attributed to the relocation of the empty carrier from inward to outward facing conformations. Detailed kinetic models have been proposed (Loo et al., 2005; Loo et al., 2006; Loo et al., 2008), including intermediate states within the empty carrier that have been found before (Krofchick et al., 2004).

Despite these prominent details about the transport mechanism, sugar binding to SGLT1 has not yet been measured directly. We recently developed a solid supported membrane-based electrophysiology (SSME) assay for SGLT1, enabling the analysis of both, binding and transport properties at the same time (Bazzone et al., 2022a). In SSME, transporter activity is triggered by substrate concentration jumps at 0 mV with the possibility to apply co-substrate gradients as an additional driving force. In SSME all recorded currents are transient and decay to zero, usually within a second, due to the capacitive read-out (Bazzone et al., 2017a; Bazzone and Barthmes, 2020). Sugar concentration jumps on SGLT1 generate biphasic transient currents comprised of a fast decaying PSS component and a slowly decaying transport component (Bazzone et al., 2022a). While the fast PSS component reflects a sugar binding induced electrogenic transition and can be also observed in the absence of Na^+^, the slowly decaying transport component is only observed when sugar binding is followed by Na^+^ translocation across the membrane.

Here, we present a detailed kinetic analysis of SGLT1 using SSME. All kinetic and thermodynamic parameters presented in the following are described in detail within the glossary. We determined I_max_, K_M_ and K_D_^app^ values for five different sugar substrates and determined rate constants for the sugar-induced electrogenic conformational transitions. We also compared the kinetics of Na^+^-, Li^+^- and H^+^-coupled transport modes and investigated the effects of Cl^−^ on SGLT1 kinetics. In addition, we examined the cross-play between cation and sugar binding and found a high degree of cooperativity for Na^+^/sugar cotransport. Finally, we propose a detailed kinetic model, focusing on the sugar translocation pathway in SGLT1, introducing a random binding order and electrogenic transitions within the substrate-bound carrier, which have not been observed before.

## 2 Materials and methods

### 2.1 Sample preparation

For all experiments, purified membrane vesicles from CHO cells overexpressing SGLT1 were used. Membrane purification was performed *via* ultracentrifugation with a sucrose gradient as described previously (Bazzone et al., 2022a). Vesicles of total protein concentrations between 2 and 5 mg/mL were stored in buffer containing 30 mM HEPES, 130 mM NMDG-Cl, 10 mM KCl, 5 mM MgCl_2_ at −80°C. Prior to sensor preparation, vesicles were usually diluted 1:10 in resting solution (R), the composition of which is defined by the type of experiment performed.

### 2.2 Sensor preparation and electrophysiological measurements

SSME was performed using the SURFE^2^R N1 device (Nanion Technologies GmbH) and sensor preparation followed the standard protocols as described in detail previously (Bazzone et al., 2017a; Bazzone and Barthmes, 2020). A summary is provided in Supplementary Methods S1.1.

If not stated otherwise, all current traces shown in the same graph were recorded on the same sensor. For analysis, only the currents during the activating solution (A) flow were used. The time resolution of the solution exchange depends on the sensor surface area, with about 3–6 ms for 1 mm sensors and 20–40 ms for 3 mm sensors (Bazzone et al., 2017a) and may limit the recording of fast PSS currents.

The buffer used to prepare the measurement solutions (R, A and non-activating solution, NA) contained 30 mM Tris/HCl, 3 mM EDTA, 1 mM EGTA and 120 mM NMDG/SO_4_ at pH 7.4. Each figure includes a scheme describing the additional components of NA, A and R solutions used for the respective experiment. Details about the preparation of measurement solutions can be found in Supplementary Methods S1.2.

### 2.3 Data recording, analysis and kinetic simulations

Current traces were recorded using the SURFE^2^R N1 Control 1.7.0 software (Nanion Technologies GmbH) and exported for further analysis using OriginPro 2022 (OriginLabs). Details about data processing including normalization and fitting procedures are provided in Supplementary Methods S1.3 to Supplementary Methods S1.6.

Reconstruction of transporter currents was accomplished using MathCAD 15 (Parametric Technology Corporation) as described previously (Tadini-Buoninsegni and Fendler, 2015). Kinetic simulations were performed with Berkeley Madonna 8.0.0 (Berkeley Madonna Inc.) using the Rosenbrock algorithm and a time filter of 2 ms, reflecting the time resolution threshold for solution exchange on 1 mm sensors.

## 3 Results

Recently we showed that SSME is able to detect both, sugar binding and Na^+^/sugar cotransport in SGLT1 (Bazzone et al., 2022a). Here, we expand our study to investigate the kinetic properties of SGLT1 regarding substrate binding and transport.

### 3.1 Concentration dependent currents under transport and PSS conditions

From concentration dependent current traces, we could determine EC_50_ values. The EC_50_ represents an apparent constant describing the half-saturation of the assessed parameter. Recently we identified that the measured current is composed of a fast decaying PSS component (*τ* = 3–10 ms) reflecting a sugar-induced electrogenic conformational transition within SGLT1 and a transport component which displays higher decay time constants (∼150 ms) (Bazzone et al., 2022a). When the detected current is dominated by transport, we assume the determined EC_50_ value to reflect the Michaelis Menten constant for Na^+^/sugar cotransport under steady-state conditions (K_M_). On the other hand, the PSS peak current is a direct consequence of sugar binding (Bazzone et al., 2022a); hence, when PSS currents are analyzed, we assume the EC_50_ matches the apparent equilibrium constant (K_D_) for sugar binding. However, the determined parameters are a result of the experimental conditions and analysis procedures, and should be interpreted as apparent constants. To distinguish experimentally determined apparent constants from real K_D_ values, we write K_D_ values derived from SGLT1 PSS currents as K_D_^app^ (apparent K_D_).

#### 3.1.1 Methods to derive K_M_ and K_D_^app^ values for D-glucose

**FIGURE 1:**
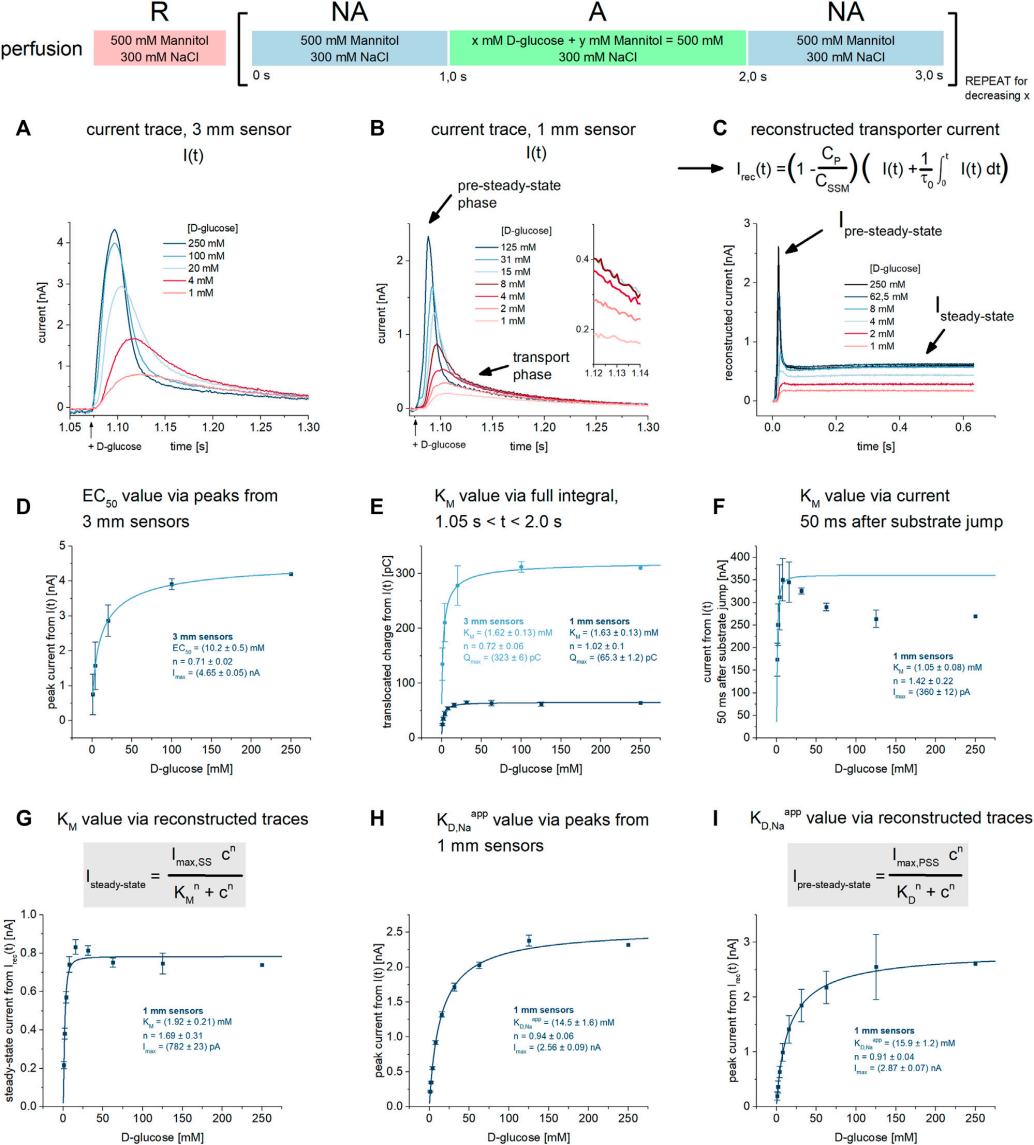
K_M_ and K_D,Na_^app^ values for D-glucose under Na^+^/D-glucose cotransport conditions. All current traces were recorded on 3 mm or 1 mm sensors as indicated, equilibrated at pH 7.4 in the presence of 300 mM Na^+^ in all measurement solutions and upon D-glucose concentration jumps between 1 mM and 250 mM. Averaged data of *n* = 3 different sensors is shown. **(A)** Representative current traces recorded on one 3 mm sensor. The large sensor surface and high signal-to-noise comes at the cost of time resolution: peak currents are defined by both PSS and transport components. **(B)** Representative current traces recorded on one 1 mm sensor. Compared to 3 mm sensors, time resolution is higher and a fast decaying PSS phase can be distinguished from the slow decaying transport phase. The inset shows the current at *t* = 1.13 s used for the analysis in (F). **(C)** *Via* circuit analysis, transporter currents were reconstructed from the current traces shown in (B) (Tadini-Buoninsegni and Fendler, 2015). Steady-state currents are revealed from the transport phase of the original current. I_pre-steady-state_ and I_steady-state_ is used for the analysis of K_M_ and K_D_ values in (G) and (I), respectively. **(D)** Peak currents from 3 mm sensors are fitted using a hyperbolic equation to derive an EC_50_ of 10.2 ± 0.5 mM for D-glucose. It represents a value which is between K_M_ and K_D,Na_^app^, since the peak is affected by both D-glucose induced PSS and transport phases. **(E)** Integrals from 3 mm to 1 mm sensors are fitted using a hyperbolic equation to derive K_M_ values of 1.62 ± 0.13 mM and 1.63 ± 0.13 mM for D-glucose, respectively. Integrals reflect the overall charge translocation which is dominated by transport; PSS charge translocation is small in comparison and may be neglected, hence the resulting EC_50_ reflects the real K_M_. **(F)** Currents 50 ms after the sugar concentration jump are fitted using a hyperbolic equation to derive a K_M_ of 1.05 ± 0.08 mM for D-glucose. At this time point, PSS currents are already zero, allowing for the read-out of the transport phase directly. The resulting EC_50_ reflects a K_M_. A decrease in current amplitude at high sugar concentrations was observed. The K_M_ was determined using the D-glucose concentrations between 1 mM and 16 mM. **(G)** Steady-state currents from the reconstructed transporter currents are fitted using a hyperbolic equation to derive a K_M_ value of 1.92 ± 0.21 mM for D-glucose. **(H)** Peak currents from 1 mm sensors are fitted using a hyperbolic equation to derive a K_D,Na_^app^ of 14.5 ± 1.6 mM for D-glucose. In contrast to 3 mm sensors and due to higher time resolution, peaks are dominated by fast PSS currents triggered by D-glucose binding to SGLT1. Hence, the resulting EC_50_ may be close to the real K_D_. **(I)** Peak currents from the reconstructed transporter currents are fitted using a hyperbolic equation to derive a K_D,Na_^app^ of 16.3 ± 1.2 mM for D-glucose.

To determine the sugar K_M_ and K_D_^app^ under Na^+^/D-glucose cotransport conditions, we performed different sugar concentration jumps using the same sensor in the presence of 300 mM NaCl in all measurement solutions. We took advantage of different sensor sizes with different time resolutions: currents recorded using 3 mm sensors exhibit a broader peak shape. Here, PSS and transport phases contribute to the peak currents (Figure 1A). Due to the higher time resolution when 1 mm sensors are used, the peaks are mainly comprised of the fast PSS current phase (Figure 1B) (Bazzone et al., 2017a). A common mathematical approach is to reconstruct the transporter current *via* circuit analysis from the raw current trace obtained by SSME (Tadini-Buoninsegni and Fendler, 2015). Through this procedure the slowly decaying current component is used to find the steady-state current amplitude, while the PSS current is unaltered (Figure 1C). This procedure helps to separate PSS and transport current phases and allows for individual analysis.

In SSME, concentration dependent peak currents are typically used to derive K_M_ values. When a transporter shows substrate-induced PSS currents, they overlay with transport currents. Consequently, the concentration dependence of the peak current may be affected by both K_M_ and K_D_^app^. Hence, using the peak currents to derive K_M_ values might not be viable, depending on the ratio of transport and PSS current amplitudes. In SGLT1, the PSS current amplitude is pronounced, dominating the peak current when 1 mm sensors with higher time resolution are used. In contrast, when using 3 mm sensors, PSS and transport current amplitudes are of similar magnitude. Hence, when 3 mm sensors are used, an EC_50_ will be derived reflecting an intermediate value between K_M_ and K_D_^app^ (Figure 1D). Here we wanted to evaluate different analysis procedures to derive K_M_ and K_D_^app^ values individually.

To derive K_M_ values from recordings on 3 mm sensors, we used the total charge translocation calculated by integrating the current time course: the overall charge translocation is dominated by transport. While PSS currents significantly affect the peak of the measured current, they are already at zero about 50 ms after solution exchange. Therefore, the impact of PSS currents on the total charge is negligible. The current integral represents an alternative read-out in SSME due to different advantages over the peak currents (Pintschovius et al., 1999; Bazzone et al., 2017b; Gerbeth-Kreul et al., 2021; Thomas et al., 2021). When plotting the concentration dependence of the translocated charge, we obtain a K_M_ of 1.62 ± 0.13 mM for SGLT1 mediated D-glucose transport (Figure 1E). Even though the PSS current amplitude is more pronounced on 1 mm sensors, the integral analysis on 1 mm sensors yields essentially the same K_M_ of 1.63 ± 0.13 mM (Figure 1E).

Another method to derive K_M_ values is to read-out the current amplitude at a time point when the PSS current has already decreased to zero. This time point is estimated from a mono-exponential fit of the fast current decay reflecting the PSS current phase. For this analysis, 1 mm sensors are preferred due to the faster decay of the PSS current phase. This allows the selection of the current at an earlier time point after the concentration jump. We plotted the current about 50 ms after the substrate jump against the sugar concentration (compare inset of Figure 1B). From this analysis we obtained a K_M_ of 1.05 ± 0.08 mM (Figure 1F). A third alternative to derive K_M_ is to use the reconstructed steady-state current. For this analysis, the dataset obtained from 1 mm sensors is used. Here, we obtain a K_M_ value of 1.92 ± 0.21 mM (Figure 1G), close to the values obtained from the other two approaches.

To distinguish K_D_^app^ values determined in the presence and absence of Na^+^, we define K_D,Na_^app^ as the apparent equilibrium constant in the presence of 300 mM Na^+^ and K_D,K_^app^ as the constant when Na^+^ is replaced by 300 mM K^+^. To obtain K_D,Na_^app^ values from PSS currents, we used 1 mm sensors. We have analyzed the peak currents from the raw traces, obtaining a K_D,Na_^app^ value of 14.5 ± 1.6 mM (Figure 1H). The analysis of the peak currents from the reconstructed transporter current yield a similar K_D,Na_^app^ value of 15.9 ± 1.2 mM (Figure 1I). These values might be a lower limit for the real K_D,Na_ due to the remaining influence of the transport phase and the fact that K_M_ < K_D_^app^. However, the Hill coefficient of all fits are close to 1, indicating sufficient analysis for the binding of one sugar molecule to SGLT1.

Since SGLT1 is predominantly in a right-side-out orientation (Bazzone et al., 2022a), we were able to distinguish K_M_ values for influx and efflux by reversing the assay conditions. We found a K_M_ for D-glucose in efflux mode of 2.2 ± 1.6 mM (Supplementary Figure S1), not significantly increased compared to the K_M_ in influx mode (Supplementary Results S2.1).

#### 3.1.2 Comparison of K_M_ values for D-glucose with the literature

The above determined K_M_ values are in agreement with values from the literature, which range from 0.2 mM to 5.4 mM, depending on the sample and assay conditions (Barfuss and Schafer, 1981; Panayotova-Heiermann et al., 1996; Díez-Sampedro et al., 2001; Hummel et al., 2011; Wright et al., 2011; Arthur et al., 2014). The broad range of values found in the literature is a result of different experimental conditions. Literature values were often obtained using conventional electrophysiology—patch clamp or TEVC—in the presence of 140 mM Na^+^ and a negative membrane potential in the physiological range. Under these conditions most authors find K_M_ values in the range of 0.5 mM (Wright et al., 2011). In contrast, the K_M_ values close to 2 mM obtained here are recorded at 0 mV and in the presence of 300 mM Na^+^. It was shown previously that the apparent affinity for MDG increases 5-fold when voltage is shifted from −10 mV to −150 mV (Panayotova-Heiermann et al., 1995; Hirayama et al., 1996). The absence of membrane potential in a typical SSME experiment explains the somewhat higher K_M_ value. We have already demonstrated that the K_M_ value decreases when a negative membrane potential is applied in an SSME assay on SGLT1 (Bazzone et al., 2022a).

#### 3.1.3 Sugar binding in the absence of Na^+^

**FIGURE 2:**
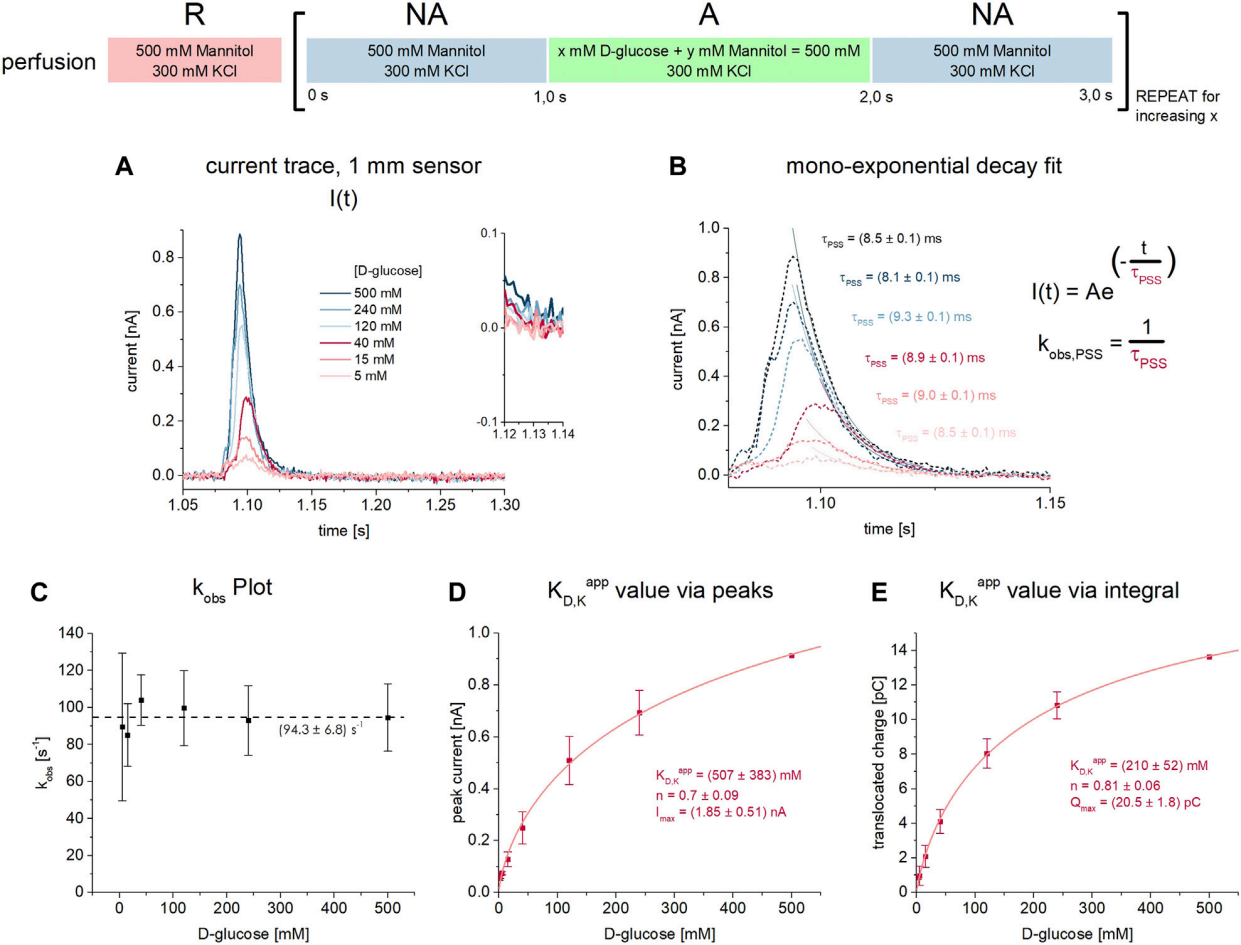
D-glucose binding to SGLT1 in absence of co-substrate. All current traces were recorded on 1 mm sensors at pH 7.4 in absence of Na^+^ upon D-glucose concentration jumps between 5 mM and 500 mM. Na^+^ was replaced by 300 mM K^+^ in all measurement solutions. Averaged data of *n* = 6 different sensors is shown. **(A)** Representative current traces recorded on one 3 mm sensor. The inset shows the current at *t* = 1.13 s which already reached the baseline, indicating that only PSS currents upon D-glucose binding are measured. **(B)** The decay time constant τ is determined from mono-exponential fits of the current decay. Rate constants k_obs_ are derived for each sugar concentration. **(C)** Rate constants k_obs_ = 1/τ for the sugar-induced PSS reaction are independent of the D-glucose concentration within the tested concentration range. **(D)** Peak currents are fitted using a hyperbolic equation to derive a K_D,K_^app^ of 507 ± 383 mM for D-glucose binding to the empty carrier. **(E)** Integrals are fitted using a hyperbolic equation to derive a K_D,K_^app^ value of 210 ± 52 mM for D-glucose binding to the empty carrier.

Since sugar binding to SGLT1 occurs in the absence of Na^+^, detected by a smaller PSS current (Bazzone et al., 2022a), we determined the K_D,K_^app^ for D-glucose. Here, 300 mM NaCl is replaced by 300 mM KCl in all measurement solutions (Figure 2). Under these conditions, we do not detect transport currents, but only PSS currents (Figure 2A).

Indicative for the absence of a transport phase is the mono-exponential current decay. 50 ms after substrate jump–the time point used to read out the transport currents in the presence of Na^+^ (Figures 1B,F)—the current already reached the baseline (Figure 2A inset). From the time constant of the exponential current decay, the rate constant of the sugar-induced electrogenic reaction (k_obs_ = 1/τ) can be derived (Figure 2B). Interestingly, k_obs_ is independent of sugar concentration for all concentrations tested (5 mM–500 mM) (Figure 2C). The average k_obs_ is 94.3 ± 6.8 s^−1^.

Since only PSS currents are detected, both peak and integral analysis may be used to derive K_D,K_^app^ values. Integrals are often used when analyzing PSS currents, since the charge translocation contains thermodynamic information about the underlying electrogenic reaction, while the peak current is a consequence of the transporter kinetics and the time resolution of the instrument, hence containing less information about the transporter. However, we obtained very similar K_D,K_^app^ values of 507 ± 383 mM (Figure 2D) and 210 ± 52 mM (Figure 2E), when using peak and integral analysis, respectively.

Summarizing the results so far, we observe an apparent affinity (K_M_) for D-glucose transport of ≈1.5 mM, while sugar binding to the Na^+^-bound carrier occurs with much lower affinity (K_D,Na_^app^ ≈15 mM); the empty carrier shows a reduced sugar affinity (K_D,K_^app^ ≈200 mM).

#### 3.1.4 Half saturation constants for Na^+^

**FIGURE 3:**
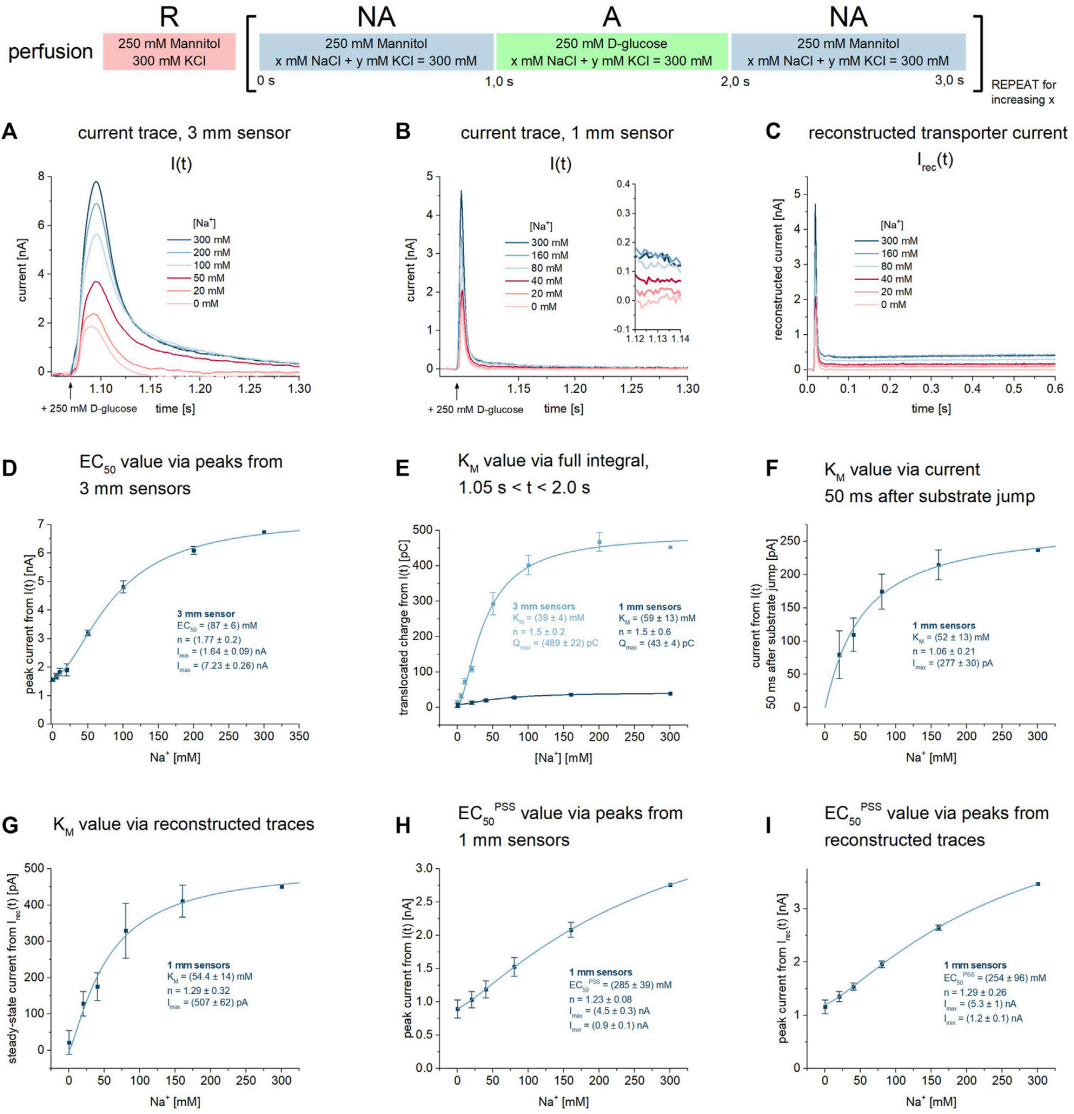
K_M_ and EC_50_^PSS^ values for Na^+^ during Na^+^/D-glucose cotransport. All current traces were recorded on 3 mm or 1 mm sensors as indicated, equilibrated at pH 7.4 in the presence of 0–300 mM Na^+^ in all measurement solutions and upon 250 mM D-glucose concentration jumps. Averaged data of *n* = 6 different sensors is shown. Further details are found in the description of Figure 1. **(A)** Representative current traces recorded on one 3 mm sensor. **(B)** Representative current traces recorded on one 1 mm sensor. The inset shows the current at *t* = 1.13 s used for the analysis in (F). **(C)** *Via* circuit analysis, transporter currents were reconstructed from the current traces shown in (B) (Tadini-Buoninsegni and Fendler, 2015). **(D)** Peak currents from 3 mm sensors are fitted using a hyperbolic equation to derive an EC_50_ of 87 ± 6 mM for Na^+^. It represents a value which is between K_M_ and the EC_50_ of the D-glucose induced PSS current, since the peak is affected by both D-glucose induced PSS and transport phases. **(E)** Integrals from 3 mm to 1 mm sensors are fitted using a hyperbolic equation to derive K_M_ values of 39 ± 4 mM and 59 ± 13 mM for Na^+^, respectively. **(F)** Currents at t = 1.13 s are fitted using a hyperbolic equation to derive a K_M_ of 52 ± 13 mM for Na^+^. **(G)** Steady-state currents from the reconstructed transporter currents are fitted using a hyperbolic equation to derive a K_M_ value of 54.4 ± 14 mM for Na^+^. **(H)** Peak currents from 1 mm sensors are fitted using a hyperbolic equation to derive an EC_50_^PSS^ of 285 ± 39 mM for Na^+^. Since peaks are dominated by D-glucose binding induced PSS currents, the determined EC_50_ may not represent a K_D_ for Na^+^ but is rather limited by sugar binding kinetics. **(I)** Peak currents from the reconstructed transporter currents are fitted using a hyperbolic equation to derive an EC_50_^PSS^ of 254 ± 96 mM for Na^+^.

To determine the K_M_ value for Na^+^, we performed 250 mM D-glucose concentration jumps using different Na^+^ concentrations in all measurement solutions (Figure 3). We performed the measurements using 3 mm sensors (Figure 3A) and 1 mm sensors (Figure 3B) and reconstructed the transporter currents (Figure 3C). The peak currents recorded on 3 mm sensors again yield an EC_50_ reflecting a mix of EC_50_ values from transport and PSS current phases (Figure 3D). Hence, we analyzed the results using the same three approaches as discussed for D-glucose above. We obtained K_M_ values of 39 ± 4 mM, 59 ± 13 mM (both Figure 3E), 52 ± 13 mM (Figure 3F) and 54 ± 14 mM (Figure 3G) using the full integrals from 3 mm to 1 mm sensors, the currents 50 ms after substrate jump and the reconstructed steady-state currents, respectively.

Altogether, the K_M_ values we obtained do match with those found in the literature. Literature values vary between 0.8 mM and 73 mM, depending on the experimental conditions. Values at the lower end of the spectrum were determined at highly negative membrane voltages (Hirayama et al., 1996; Panayotova-Heiermann et al., 1996; Quick et al., 2001; Loo et al., 2006). Between 0 and -60 mV, K_M_ values are between 35 mM and 73 mM (Hirayama et al., 1996; Loo et al., 2000; Eskandari et al., 2005; Hummel et al., 2011), which agree with our results. Wright et al. found K_M_ values of 60 mM at 0 mV and 0.8 mM at −150 mV (Wright et al., 2011).

We then used the Na^+^ dependence of the sugar-induced PSS current to derive an EC_50_^PSS^ value that might correlate with a K_D_^app^ for Na^+^. We have used the peak current amplitudes recorded on 1 mm sensors, either from the raw current traces or from the reconstructed transporter currents. Both approaches yield very similar EC_50_^PSS^ values of 285 ± 39 mM (Figure 3H) and 254 ± 96 mM (Figure 3I), respectively. This is almost one order of magnitude higher compared to the Na^+^ K_M_, similar to the K_D,Na_^app^/K_M_ ratio for D-glucose (15 vs. 2 mM). A K_D_ for Na^+^ of 20 mM was reported in the literature (Loo et al., 2013). Hence, we suspect that the Na^+^ EC_50_^PSS^ is limited by sugar binding kinetics and does not represent a real K_D_ for Na^+^. Real K_D_ values for Na^+^ may be determined *via* Na^+^ concentration jumps using different Na^+^ concentrations in the presence of sugar. However, Na^+^-induced PSS currents are smaller, more affected by the overlay of transport currents and therefore, difficult to analyze (Bazzone et al., 2022a). K_D_ values for Na^+^ in the absence of sugar cannot be determined, since Na^+^ binding in the absence of sugar does not trigger PSS currents in SSME (Bazzone et al., 2022a).

Since sugar binding induces PSS currents in the absence of Na^+^ (Figure 2), we obtain a Na^+^ concentration dependence of the PSS starting from I_min_ (Figures 3H,I). The hyperbolic fit thus also reveals the ratio of PSS electrogenicity when sugar binds to the Na^+^-bound carrier vs. the empty carrier, which is about I_max_/I_min_ = 4.5. Na^+^ binding has a large effect on the electrogenicity of the sugar-induced conformational transition.

#### 3.1.5 Rate constants for the sugar-induced PSS

**FIGURE 4:**
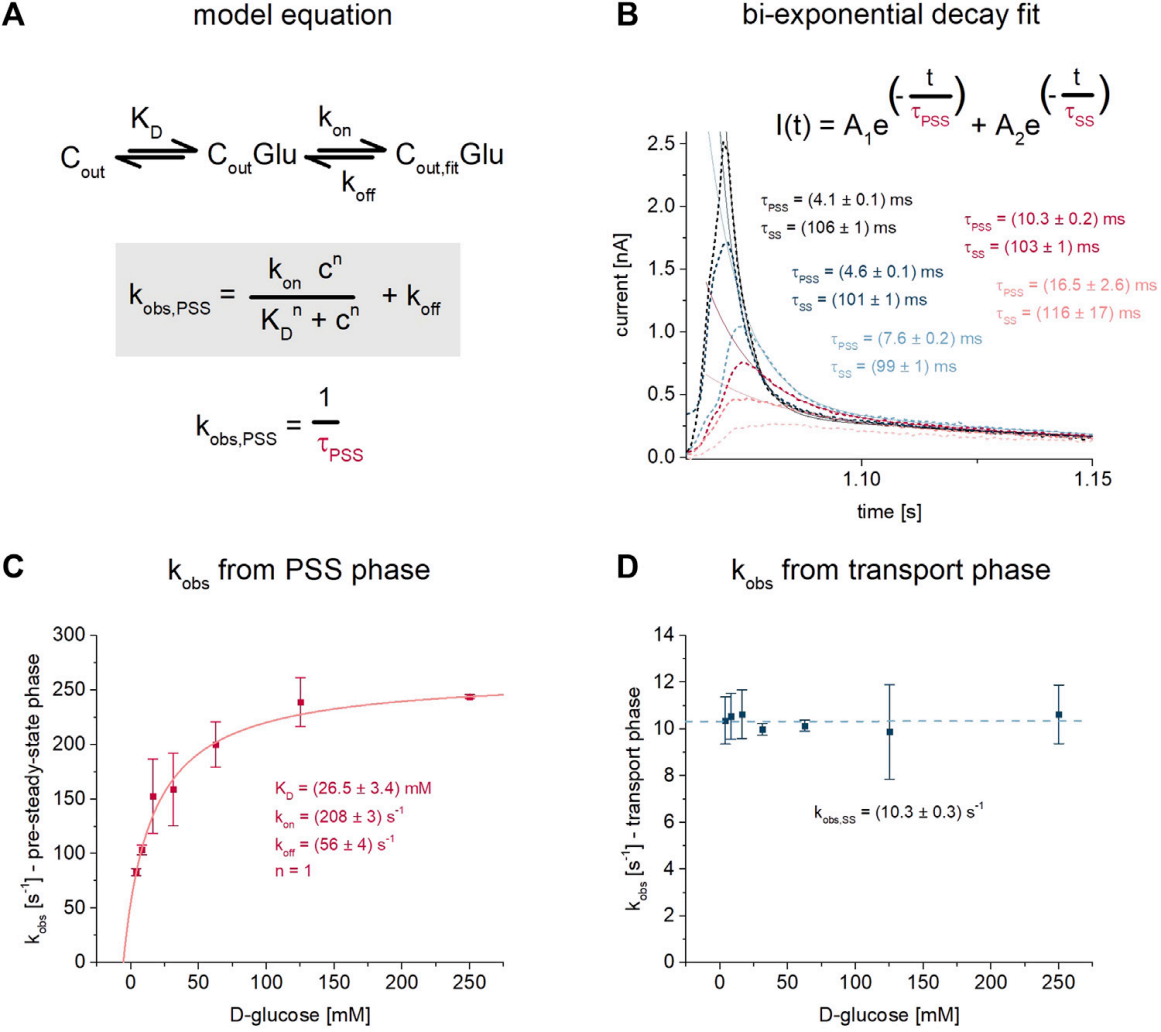
D-glucose binding to SGLT1 in the presence of Na^+^. For this analysis the dataset recorded on 1 mm sensors shown in Figure 1 was used. **(A)** Kinetic model and model equation for D-glucose (Glu) binding to SGLT1 in the outward facing conformation (C_out_) followed by an electrogenic conformational transition from C_out_Glu to C_out, fit_Glu–likely representing an induced fit mechanism. Sugar binding is defined *via* the K_D_, while the conformational transition is defined *via* the rate constants k_on_ and k_off_. The observed rate constant k_obs,PSS_ = 1/τ_PSS_ can be written as a function of K_D_, k_on_, k_off_ and the sugar concentration c. The time constant τ is the decay time from an exponential fit of the sugar-induced PSS current. **(B)** D-glucose concentration dependent traces are taken from Figure 1B. Two decay time constants are derived from bi-exponential fits of the current decay: τ_PSS_ for the fast-decaying PSS current and τ_SS_ for the slow-decaying transport phase. τ_PSS_ depends on the sugar concentration according to the model equation. τ_SS_ is a result of the vesicles being charged upon transport, leading to decreasing transport rates over time; it is virtually independent of sugar concentration. **(C)** Concentration dependent values for k_obs,PSS_ were determined from n = 5 different sensors and averaged, then fitted using the model equation shown in (A). For the fit, the Hill coefficient n was fixed to 1 representing one sugar molecule binding to SGLT1, while values for K_D_ = 26.5 ± 3.4 mM, k_on_ = 208 ± 3 s^−1^ and k_off_ = 56 ± 4 s^−1^ were determined. The great fit (*R* ^2^ > 0.999) validates the use of the model equation for our data. The K_D_ is slightly higher compared to the K_D_^app^ values obtained from the analysis procedures given in Figure 1. **(D)** Values for k_obs,SS_ were averaged from the same n = 5 sensors as used for the k_obs,PSS_ analysis shown in (C). Averaged values were plotted to show that the decay of the transport phase is independent of sugar concentration. k_obs_ of 10.3 ± 0.3 s^−1^ represents the average for all sugar concentrations.

The existence of substrate-induced PSS currents enables kinetic analysis of the underlying reaction. We propose a simple kinetic model as shown in Figure 4A: sugar binding with the affinity K_D_ is followed by an electrogenic transition with rate constants k_on_ and k_off_ which is detected *via* SSME. Note that k_on_ and k_off_ do not represent rates of sugar binding, but rates of a subsequent conformational transition.

The rate constants k_on_ and k_off_ may be derived from the concentration dependent decay time constant τ_PSS_ = 1/k_obs_ of the sugar-induced PSS currents, as demonstrated earlier for sugar-induced PSS currents of H^+^-coupled sugar transporters recorded *via* SSME (Garcia-Celma et al., 2010; Bazzone et al., 2022b). τ_PSS_ is accessible *via* exponential fit of the current decay. Surprisingly, and as mentioned above, τ_PSS_ in the absence of Na^+^—reflecting sugar binding to the empty carrier—did not depend on sugar concentration (Figure 2C). In contrast, in the presence of Na^+^, τ_PSS_ does depend on sugar concentration. τ_PSS_ in the presence of Na^+^ was determined using a bi-exponential fit of the current decay (Figure 4B). The decay time constant of the fast current reflects τ_PSS_. It increases when sugar concentrations are reduced. This correlates with a slower reaction rate k_obs_ = 1/τ_PSS_. The highest concentration of 250 mM D-glucose corresponds with the lowest decay time constant of 4.10 ± 0.03 ms (k_obs_ = 244 ± 2 s^−1^), which is close to the time resolution of the measurement for 1 mm sensors (Bazzone et al., 2017a). Hence the maximum k_obs_ may be considered a lower limit. The highest PSS decay time constant of 12.05 ± 0.47 ms (k_obs_ = 83 ± 3 s^−1^) was determined for 4 mM D-glucose. Based on the k_obs_ values and using the model equation provided in Figure 4A, we found k_on_ = 208 ± 3 s^−1^ and k_off_ = 56 ± 4 s^−1^ and a K_D,Na_^app^ of 26.5 ± 3.4 mM (Figure 4C). The K_D,Na_^app^ determined with the model equation is slightly higher compared to the K_D,Na_^app^ determined from the peak currents (14.5 ± 1.6 mM, Figure 1H). As mentioned above, using the peak currents might underestimate the K_D_, due to the overlay with transport current phases with lower EC_50_ values.

In contrast to τ_PSS_, the slow decay time constant τ_SS_ representing the transport current phase does not depend on sugar concentration (Figure 4D). τ_SS_ is a result of the increasing membrane potential - due to Na^+^/sugar cotransport - that acts as a counter force for the sugar concentration gradient. This leads to decreasing transport rates over the course of the real-time measurement until a new equilibrium is reached at which no net transport occurs. For our analysis, τ_SS_ contains no interesting information about the transporter, but needs to be considered to achieve a proper fit of the bi-exponential current decay, when transport and PSS current phases are detected.

#### 3.1.6 Cooperativity of Na^+^ and sugar binding

**FIGURE 5:**
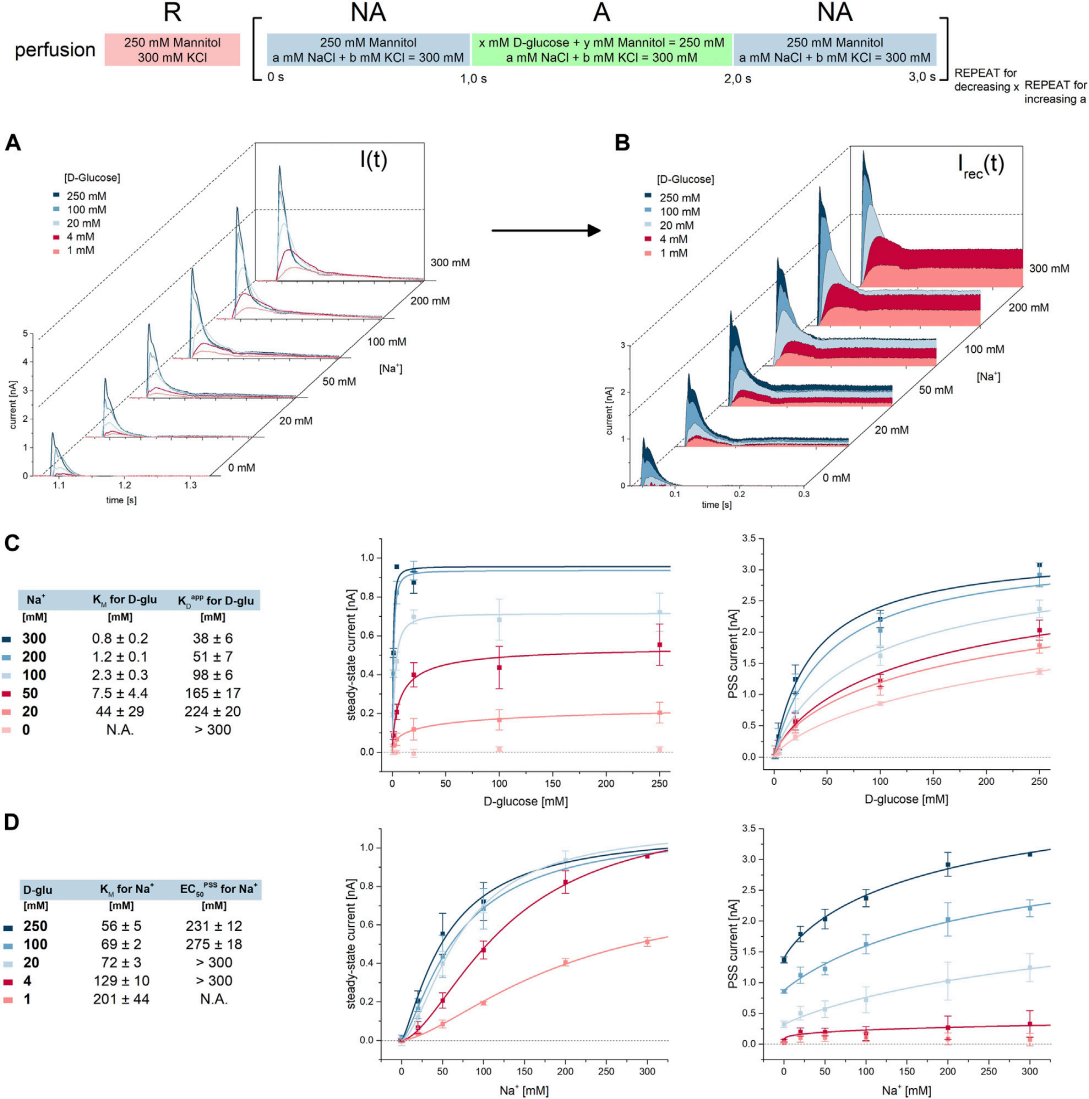
Cooperativity between D-glucose and Na^+^ during Na^+^/D-glucose cotransport. All current traces were recorded on 1 mm sensors at pH 7.4 in the presence of 0 mM–300 mM Na^+^ in all measurement solutions and upon D-glucose concentration jumps between 1 mM and 250 mM. All 30 combinations of Na^+^ and D-glucose concentrations were measured on the same sensor, for *n* = 5 sensors in total. **(A)** Representative current traces recorded on one sensor. D-glucose concentrations are color-coded according to the figure legend. One graph shows all traces for one given Na^+^ concentration, which is indicated to the right of the graph. **(B)** *Via* circuit analysis, transporter currents were reconstructed from the current traces shown in (A) (Tadini-Buoninsegni and Fendler, 2015). Steady-state currents are used to derive K_M_ values for D-glucose and Na^+^ in the presence of different concentrations of co-substrate. PSS peak currents are used to derive K_D_^app^ values for D-glucose and EC_50_^PSS^ values for Na^+^, depending on co-substrate concentration. **(C)** Hyperbolic fits of I_SS_ and I_PSS_ for different D-glucose concentrations to derive K_M_ and K_D_^app^ values for D-glucose in the presence of different Na^+^ concentrations. **(D)** Hyperbolic fits of I_SS_ and I_PSS_ for different Na^+^ concentrations to derive K_M_ and EC_50_^PSS^ values for Na^+^ in the presence of different D-glucose concentrations.

Na^+^ binding triggers the opening of an extracellular gate within SGLT1, allowing glucose to bind in the central cavity (Peerce and Wright, 1984; Hirayama et al., 2007; Sala-Rabanal et al., 2012; Loo et al., 2013; Adelman et al., 2016; Gorraitz et al., 2017). Seemingly contradictory to this, we previously found that glucose binds to SGLT1, even in the absence of Na^+^ (Bazzone et al., 2022a). In the results described above we showed that Na^+^ availability increases the affinity for D-glucose from 200 mM (Figure 2E) to 15 mM (Figure 1H). In order to investigate the extent of cooperativity between sodium and sugar, we used five glucose concentrations (1 mM, 4 mM, 20 mM, 100 mM, 250 mM) to activate SGLT1 in the presence of six different sodium concentrations (0 mM, 20 mM, 50 mM, 100 mM, 200 mM, 300 mM), making up for 30 different conditions; All 30 experiments were performed sequentially on a single 1 mm sensor (Figure 5A). We have repeated this set of experiments with 5 sensors in total.

To determine K_M_ and K_D_^app^ values for D-glucose and K_M_ and EC_50_^PSS^ values for Na^+^ we reconstructed the transporter currents, allowing read-out of steady-state and PSS currents individually (Figure 5B). We then plotted the concentration dependent steady-state and PSS currents to determine K_M_ and K_D_^app^ or EC_50_^PSS^ values, respectively.

For D-glucose we obtained K_M_ and K_D_^app^ values in the presence of six sodium concentrations (Figure 5C): when the Na^+^ concentration is reduced from 300 mM to 20 mM, K_M_ for the sugar increases 55-fold from 0.8 mM to 44 mM, while K_D_^app^ for the sugar increases 6-fold from 38 mM to 224 mM. Compared to the analysis of the dataset shown in Figure 1, the determined K_D_^app^ value is somewhat higher, and K_M_ is somewhat lower. This is probably due to the lower amount of data points per fit. In the literature we found that K_M_ for MDG increases 2.3-fold at −150 mV and 5.8-fold at −70 mV, when the Na^+^ concentration decreases from 100 mM to 25 mM (Wright et al., 2011). If the impact of Na^+^ concentration on the K_M_ for MDG is higher when voltage is further increased, the 55-fold increase in K_M_ we observed at 0 mV conditions seems plausible.

For Na^+^ we obtained K_M_ and EC_50_^PSS^ values in the presence of five glucose concentrations (Figure 5D): reduction of D-glucose concentration from 250 mM to 1 mM leads to a 3.6-fold increase in K_M_ from 56 mM to 201 mM. The EC_50_^PSS^ increases from 231 mM to >300 mM. These values are consistent with the results from the analysis of the dataset shown in Figure 3. Comparing with the literature, Wright et al. found the K_M_ for Na^+^ increased 2.7-fold at −150 mV and 2.1-fold at −70 mV, when MDG concentration decreases from 10 mM to 1 mM (Wright et al., 2011). Since the change in Na^+^ K_M_ with sugar concentration is less affected by membrane voltage, it correlates well with the 3.6-fold increase we found at 0 mV.

The correlation between K_M_ and co-substrate concentrations for both substrates support a random binding order. We also fitted the cooperativity data using model equations for sequential substrate binding, assuming that either Na^+^ or the sugar binds first. Both model fits approximate the respective datasets (Supplementary Results S2.2; Supplementary Figure S2).

### 3.2 Binding and transport of different sugar species

**FIGURE 6:**
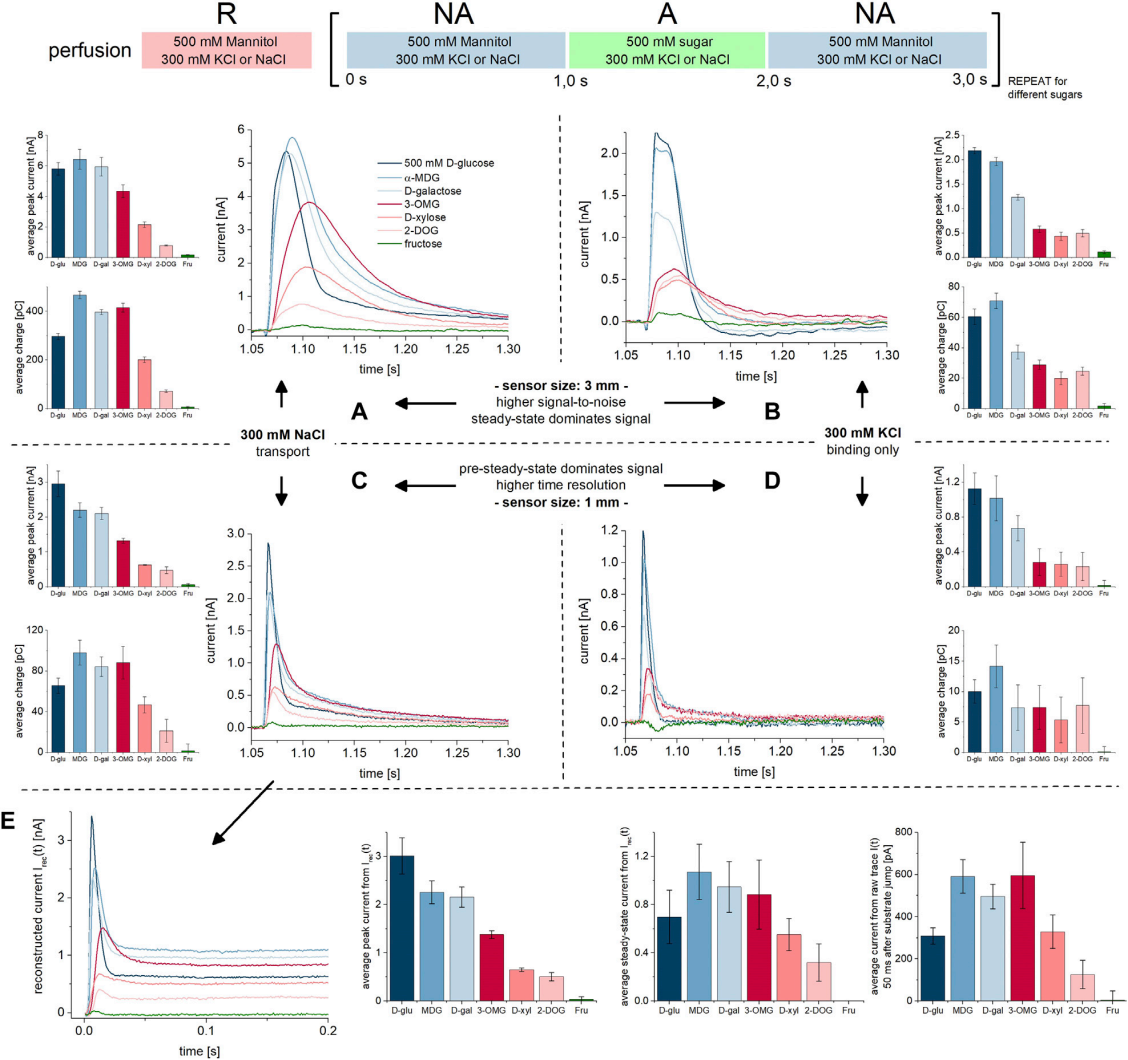
Na^+^/sugar transport and PSS current traces for different sugar substrates. All current traces were recorded at pH 7.4, either in the presence of 300 mM Na^+^ (left) or 300 mM K^+^ (right) in all measurement solutions and upon 500 mM concentration jumps of different sugars. We have compared the current traces recorded on 3 mm sensors (top) and 1 mm sensors (bottom). At given conditions, all sugar-induced traces were recorded on the same sensor. As a negative control D-fructose was used (green traces), which is not a substrate of SGLT1. The bar plots in **(A–D)** show averaged peak currents and charges (integrals) for all substrates under the given conditions and recorded from a total set of *n* = 3 sensors each. Standard deviations are a result of deviations across different sensors (no normalization was applied); the relative currents and charges between substrates are more accurate. The current traces shown are representative currents recorded from the same sensor. **(A)** Current traces recorded on 3 mm sensors in the presence of 300 mM Na^+^. The currents include transport and PSS current phases. From the right-shift of the peak current for OMG, D-xylose and DOG (red traces) we conclude that peaks are dominated by the transport current phase for the minor substrates. For D-glucose, MDG and D-galactose (blue traces) the fast PSS current phase dominates transport. **(B)** Current traces recorded on 3 mm sensors in the absence of Na^+^, which is replaced by 300 mM K^+^. The currents consist only of the PSS current phase resulting from sugar binding to the empty carrier. PSS current amplitudes are higher for the major substrates D-glucose, MDG and D-galactose, possibly due to a higher Q_max_, faster kinetics and/or higher affinity. **(C)** Same experiment as shown in (A) but recorded on 1 mm sensors. In contrast to 3 mm sensors, the PSS component is pronounced due to the higher time resolution of the sensors. **(D)** Same experiment as shown in (B) but recorded on 1 mm sensors. The higher time resolution allows for conclusions about PSS kinetics. **(E)** Transporter currents were reconstructed from currents shown in (C) *via* circuit analysis (Tadini-Buoninsegni and Fendler, 2015). The first two bar plots show average peak currents and steady-state currents from the reconstructed transporter currents. Peak currents approximate the PSS currents. The third bar plot shows average currents 50 ms after substrate jump from the raw current traces shown in (C), which was used as a potential read-out for the transport current and mostly matches with the relative change of the steady-state current from I_rec_(t).

We performed concentration jumps using different sugar species to determine substrate specificity. We have tested D-glucose, α-methyl-D-glucose (MDG), D-galactose, 3-O-methyl-D-glucose (OMG), D-xylose and 2-desoxy-D-glucose (DOG) in the presence and absence of Na^+^ using both 1 mm and 3 mm sensors (Figure 6). All sugar substrates were applied on the same sensor to achieve direct comparison of relative signal amplitudes and we compared both peak currents and the overall charge translocation (current integrals), for all sugar substrates.

We used 500 mM sugar in order to detect binding and transport, also for sugars with very low apparent affinities such as DOG [K_M_>100 mM (Díez-Sampedro et al., 2001; Tyagi et al., 2007)] and D-xylose [K_M_≈50–100 mM (Hager et al., 1995; Díez-Sampedro et al., 2001)]. Tyagi et al. could not detect transport for DOG using radiolabeled substrates in the presence of a Na^+^ gradient (Tyagi et al., 2007). However, we measured transport currents (Figure 6), showing that SSME provides a higher resolution combined with a shorter measurement time compared to radiolabeled assays.

As a negative control we also included D-fructose which is not a substrate of SGLT1 (Wright et al., 2011; Kamitori et al., 2022): accordingly, D-fructose does not generate any significant currents when applied, independent of the availability of Na^+^ or sensor type used (Figure 6, green traces). All other tested sugars generate currents in the presence (Figures 6A,C) and absence (Figures 6B,D) of Na^+^, reflecting a combination of PSS and transport currents, and PSS currents alone, respectively.

In the presence of Na^+^, and using saturating concentrations of D-glucose, MDG and D-galactose (major substrates), PSS currents have a major impact on the detected peak currents, while OMG, D-xylose and DOG (minor substrates) -induced peak currents are dominated by transport. This is concluded from three different observations: (1) When PSS currents dominate the signal, peak currents are high, but the translocated charge is low–as observed for D-glucose (Figure 6C, dark blue bars). When transport dominates the signal, translocated charge is high, but not necessarily the peak current, as clearly visible for OMG (Figure 6C, dark red bars). (2) The currents induced by the major substrates show a faster decay and a left shift of the peak compared to the currents induced by minor substrates, best visible when 3 mm sensors are used (Figure 6A). A steep current rise indicates an early electrogenic step in the transport cycle, potentially generating a PSS current. In addition, the currents induced by major substrates show a bi-exponential instead of mono-exponential decay, which is best resolved on 1 mm sensors (Figure 6C). Bi-exponential currents indicate the presence of a PSS current in addition to the slow transport current. (3) Our data recorded in the absence of Na^+^ show that the major substrates (Figures 6B,D, blue traces) indeed induce larger PSS currents compared to the minor substrates (Figures 6B,D, red traces).

Following this analysis, we performed concentration-dependent measurements of all sugars in the presence and absence of Na^+^ and derived relative I_max_, K_M_ and K_D_^app^ values, and rate constants (k_obs_, k_on_ and k_off_) for the electrogenic PSS reaction. We applied the same assays and analysis methods as discussed for D-glucose and as shown in Figures 1, 2, 4. The results are presented in the following paragraphs. All traces and fits are shown in Supplementary Figures S3–S5. An overview of all kinetic and thermodynamic parameters for the different sugar species is shown in Table 1.

**TABLE 1:** Kinetic parameters obtained from transport and PSS currents using different sugar substrates in the presence and absence of Na^+^.

		Label	Parameter	D-glucose	α-MDG	D-galactose	3-OMG	D-xylose
Sugar-induced currents in the presence of 300 mM Na^+^	Transport Supplementary Figure S3	A	I_max,rec_ [pA]	698 ± 20	1,074 ± 19	961 ± 35	1,338 ± 47	902 ± 215
		B	I_max,t=50ms_ [pA]	308 ± 10	592 ± 8	508 ± 9	669 ± 31	436 ± 65
		C	Q_max,0-1s_ [pC]	65.7 ± 1.2	101 ± 2	97 ± 2	110 ± 6	129 ± 74
		D	K_M_ (I_rec_) [mM]	1.92 ± 0.21	4.1 ± 0.3	10.7 ± 1.1	241 ± 18	305 ± 167
		E	k_obs_ [s^−1^]	10.3 ± 0.3	10 ± 0.5	11.6 ± 0.7	8 ± 1.3	7.4 ± 0.8
	Pre-steady-state Supplementary Figure S4	F	l_max_ [nA]	3.06 ± 0.1	2.39 ± 0.07	2.39 ± 0.11	2.47 ± 0.28	> 0.7
		G	Q_max,0-0.03s_ [pC]	34 ± 0.1	40.3 ± 0.6	39.2 ± 1	45.6 ± 9	> 15
		H	K_D,Na_^app^ (I_peak_) [mM]	14.4 ± 1.6	32 ± 3	49 ± 7	337 ± 105	>> 500
		I	k_on_ [s^−1^]	208 ± 3	187 ± 17	145 ± 17	95 ± 5	N.A.
			k_off_ [s^−1^]	56 ± 4	≈0	≈0	2 ± 1	N.A.
Sugar-induced currents in the absence of Na^+^	Pre-steady-state Supplementary Figure S5	J	I_max_ [nA]	2.27 ± 0.62	1.51 ± 0.24	>> 0.7	>> 0.25	>> 0.25
		K	Q_max_ [pC]	15 ± 1.4	21.3 ± 2.4	18.3 ± 2.4	19.2 ± 9.7	> 6
		L	k_obs_ [s^−1^]	94 ± 7	74 ± 5	84 ± 4	36 ± 1	37 ± 8
		M	K_D,K_^app^ (Q) [mM]	210 ± 52	234 ± 64	851 ± 262	734 ± 534	>> 500
Derived constants		N	I_max,rec_/K_M_ [pA/mM]	364	262	90	5.6	3.0
		O	I_max,rec_/K_D,Na_ [pA/mM]	48.5	33.6	19.6	4.0	< 1.8
		P	K_D,K_/K_D,Na_	14.6	7.3	17.4	2.2	N.A.
		Q	K_D,Na_/K_M_	7.5	7.8	4.6	1.4	> 1.6
		R	K_D,K_/K_M_	109	57	80	3.0	> 1.6

Current traces and fits are shown in Supplementary Figures S3, S4, S5. A complete overview comparing obtained parameters by different analysis methodologies as discussed for D-glucose is provided in Supplementary Table S1. One concentration sequence for a given sugar species was measured on the same sensor, with different sugars measured on different sensors. All I_max_ and Q_max_ values for each sugar species were obtained *via* normalization using the datasets shown in Figure 6. Here, all sugar species were recorded on the same sensor. As a result, I_max_ and Q_max_ data represent proper relative values across different sugar substrates. **(A)** I_max,rec_ is obtained from a hyperbolic fit of the steady-state current as shown in Figure 1G for D-glucose. The steady-state current was calculated *via* reconstruction as shown in Figure 1C. **(B)** I_max,t=50ms_ is obtained from a hyperbolic fit of the current 50 ms after sugar jump as shown in Figure 1F for D-glucose. At this time point, PSS currents decayed to zero and the remaining transport current can be determined. It is lower compared to I_max,rec_, due to the membrane voltage which is generated upon Na^+^/sugar cotransport. **(C)** Q_max,0–1.0s_ is obtained from a hyperbolic fit of the current integral as shown in Figure 1E for D-glucose and mainly affects the charge translocation upon Na^+^/sugar cotransport. The integration was performed from the time point of substrate addition (t = 0 s) until the wash out of substrate starts (t = 1 s). The transient current decayed to zero within this time window for all sugar substrates. **(D)** K_M_ (I_rec_) was obtained from the same data fit as I_max,rec_ (A). **(E)** k_obs_ represents the slow decay time constant obtained from the bi-exponential fit of the current decay as shown in Figure 4B for D-glucose. It is a consequence of steady-state charge translocation, that decelerates transport due to the generated membrane voltage acting as a counterforce for the substrate gradient. **(F)** I_max_ is obtained from a hyperbolic fit of the peak current as shown in Figure 1H for D-glucose. For the main substrates, it is mainly affected by the PSS component, hence K_D_^app^ values may be obtained. **(G)** Q_max,0–0.03s_ is obtained from a hyperbolic fit of the current integral within the first 30 ms upon substrate jump. It is mainly affected by the PSS charge translocation. It provides indication if different sugar species induce different conformational states within SGLT1 upon binding. For minor substrates this value may be error-prone due to the low PSS to steady-state ratio. In any case, the value reflects an upper limit for PSS Q_max_. **(H)** K_D,Na_^app^ (I_peak_) was obtained from the same data fit as I_max_ (E). **(I)** k_on_ and k_off_ values were determined using the model equation shown in Figure 4. From the fit we determined negative k_off_ values for MDG and D-galactose. Hence, we fixed the fitting parameter to 0 s^−1^. Fitting the k_obs_ datasets for MDG, D-galactose and OMG required to fix the respective K_D_^app^ to the value determined from the PSS peak currents. This procedure was used before (Garcia-Celma et al., 2010) and seems valid since K_D_^app^ values derived from k_obs_ (Figure 4) and peak currents (Figure 1H/I) for D-glucose are similar. In the case of D-glucose, we could use a free K_D_ for k_on_ and k_off_ determination, due to the pronounced PSS component that made the k_obs_ accessible for a broader concentration range. **(J)** I_max_ for the sugar-induced PSS current in absence of Na^+^ is obtained from a hyperbolic fit of the peak current as shown in Figure 2C for D-glucose. **(K)** Q_max_ for the sugar-induced PSS current in absence of Na^+^ is obtained from a hyperbolic fit of the current integral as shown in Figure 2D for D-glucose. **(L)** k_obs_ is not sugar concentration dependent within the tested range of sugar concentrations (10–500 mM), hence no k_on_ and k_off_ values could be derived. An average k_obs_ is provided instead, as shown in Figure 2B for D-glucose, obtained from mono-exponential fits of the current decay. **(M)** K_D,K_^app^ (Q) was obtained from the same data fit as Q_max_ (J). **(N–R)** Given ratios reflect the sugar specificities at different levels, each calculated based on the results provided in this table. I_max,rec_/K_M_ reflects the major sugar specificity value based on the apparent affinity under steady-state conditions and the maximum transport rate. I_max,rec_/K_D,Na_ represents a similar value, but takes into account the affinity in the presence of co-substrate, instead of the K_M_. K_D,K_/K_D,Na_ represents the factor of affinity increase due to cooperativity when sugar binding to the empty carrier and the Na^+^-bound carrier is compared. K_D,Na_/K_M_ represents the factor the apparent affinity is increased during transport compared to the binding affinity for the Na^+^-bound carrier. K_D,K_/K_M_ shows the total increase in apparent affinity comparing binding to the empty carrier and the apparent affinity under steady-state conditions.

#### 3.2.1 Substrate specificity is determined by increased apparent affinity

We determined relative I_max_ and K_M_ values for all sugar species using sugar concentration jumps in the presence of 300 mM Na^+^ on 1 mm sensors. All concentration jumps of one given sugar species were performed on the same sensor. We reconstructed the transporter currents *via* circuit analysis (Tadini-Buoninsegni and Fendler, 2015) and used the steady-state currents to derive K_M_ and I_max_ values. I_max_ values are a consequence of steady-state Na^+^ translocation coupled to the respective sugar species and driven by the sugar gradient alone. SSME does not allow for conclusions about absolute turnover rates (V_max_ in molecules per second) since the total amount of transporters on the sensor surface is unknown. But relative I_max_ corresponds to the relative translocation rates for different sugar species. To determine relative I_max_ values for different sugars, we normalized all datasets to the relative steady-state current obtained from 500 mM sugar jumps on the same sensor (Figure 6E). This way we removed the current deviation obtained when measurements on different sensors are compared.

Sugar substrates can be ordered by their relative I_max_ values (Supplementary Figure S3; Table 1): OMG (1.34 nA) > MDG (1.08 nA) > D-galactose (0.96 nA) > D-xylose (0.9 nA) > D-glucose (0.7 nA). Interestingly, the sugars with higher I_max_ mostly show lower apparent affinity (higher K_M_ values), indicating a relation between the affinity and the maximum transport rate, V_max_. The order of substrates from low to high K_M_ is: D-glucose (1.9 mM) < MDG (4.1 mM) < D-galactose (10.7 mM) < OMG (241 mM) < D-xylose (305 mM). Substrate specificity can be represented as V_max_/K_M_. While I_max_ only varies by a factor of 1.9 between tested substrates, K_M_ values differ by more than 2 orders of magnitude, hence defining the substrate specificity in the order: D-glucose (364 pA/mM) > MDG (262 pA/mM) > D-galactose (90 pA/mM) > OMG (5.6 pA/mM) > D-xylose (3.0 pA/mM).

#### 3.2.2 Comparison of relative I_max_ and K_M_ values with the literature

Differences in K_M_ values between D-glucose, MDG and D-galactose seem to be only minor or not detectable in many published studies (Panayotova-Heiermann et al., 1995; Díez-Sampedro et al., 2000; Díez-Sampedro et al., 2001; Tyagi et al., 2007). One study found K_M_ values of 1.8 mM, 3.8 mM and 6.1 mM for the three substrates at −60 mV and 37°C (Hummel et al., 2011) that do match with our results at 0 mV and room temperature (1.9 ± 0.2 mM, 4.1 ± 0.3 mM and 10.7 ± 1.1 mM, respectively).

One discrepancy is the high K_M_ value obtained for OMG of 241 ± 18 mM. In the literature we found values between 3.3 mM and 22 mM (Hirayama et al., 1996; Díez-Sampedro et al., 2001; Yoo and Lee, 2006; Tyagi et al., 2007). On the other hand, the K_M_ value of 305 ± 167 mM we determined for D-xylose is closer to the values of 50–100 mM reported in the literature (Hager et al., 1995; Díez-Sampedro et al., 2001).

Information about relative I_max_ values for different sugar species is limited. However there are indications that I_max_ is very similar across different sugar species (Birnir et al., 1991; Hirayama et al., 1997; Díez-Sampedro et al., 2001). Using SSME we found small, but significant, differences in I_max_ between substrates, which were not resolved before.

#### 3.2.3 Binding cooperativity and improved kinetics are mechanisms to enhance substrate specificity

When sugar substrates are ordered by their K_D,Na_^app^ values, it is the same sequence as for the K_M_ (Supplementary Figure S4; Table 1). However, K_D,Na_^app^ values are dramatically increased compared to K_M_ values, especially for the major substrates: D-glucose (14.4 mM) < MDG (32 mM) < D-galactose (49 mM) < OMG (337 mM) < D-xylose (>500 mM). Interestingly, the ratio between K_D,Na_^app^ and K_M_ is only >>1 for the major substrates D-glucose (7.5), MDG (7.8) and D-galactose (4.6), but not for OMG (1.4) and D-xylose (>1.6). This may indicate that evolution optimized steady-state transport kinetics around the major substrates to reduce K_M_ for D-glucose transport.

In the absence of Na^+^, no transport currents are observed. To determine K_D,K_^app^ values we have used integral analysis (Supplementary Figure S5; Table 1). When sugars are ordered by their K_D,K_^app^ values, the sequence matches with those sequences stated above for K_D,Na_^app^ and K_M_ values, although differences across substrates are not as strong. The affinity is massively decreased for almost all sugar substrates, when Na^+^ is not available: D-glucose (210 ± 52 mM) ≈ MDG (234 ± 64 mM) < D-galactose (851 ± 262 mM) ≈ D-xylose (734 ± 534 mM) < OMG (>>500 mM). Sugars may be ordered by K_D,K_/K_D,Na_ ratio reflecting the degree of cooperativity between Na^+^ and sugar binding. The highest K_D,K_/K_D,Na_ ratio is observed for D-galactose (17.4), followed by D-glucose (14.6) and MDG (7.3), while the apparent affinity for OMG (2.2) is not as much affected by Na^+^ binding. This shows that binding cooperativity is optimized for the major substrates as well. The K_D,K_/K_D,Na_ ratio for D-xylose could not be determined due to the high K_D_^app^ in both the presence and absence of Na^+^.

In summary, both major and minor substrates have poor affinities for SGLT1 in the empty carrier state, i.e., a high K_D,K_^app^. Substrate specificity for the main substrates is a result of optimized cooperative binding with Na^+^ as well as improved transport kinetics to further enhance the K_M_ below the K_D,Na_^app^.

#### 3.2.4 Sugar binding is fast for all sugars

We determined k_obs_ values from the PSS current decay in the presence and absence of Na^+^ for all sugar species and found that the sugar-induced conformational transition is faster than the estimated transport rate of 28 s^−1^ found in literature (Loo et al., 2005). Thus, the sugar-induced conformational transition unlikely represents the rate limiting step in Na^+^/D-glucose co-transport. However, rates differ for each sugar species (Supplementary Figures S4, S5; Table 1).

In the presence of Na^+^, the sugar species may be ordered *via* k_on_ rates of the conformational transition: D-glucose (208 s^−1^) > MDG (187 s^−1^) > D-galactose (145 s^−1^) > OMG (95 s^−1^); the k_on_ rate for D-xylose could not be determined due to the low ratio of PSS to steady-state currents. The respective k_off_ rates are close to 0 s^−1^ for all sugars, but D-glucose (56 s^−1^). Given errors of data fitting (Figure 4C), k_off_ values not distinguishable from 0 s^−1^ may represent values of up to 5 s^−1^. The low k_off_ rates for all tested sugars but D-glucose suggest that most sugars enable a higher stability of sugar-bound SGLT1, once the conformational transition after sugar binding is completed. Interestingly, this contrasts with the observation that all tested sugars show a lower affinity (higher K_D_) compared to D-glucose (Section 3.2.3).

As for D-glucose binding to the empty carrier (Figure 2), we could not determine k_on_ and k_off_ values for the electrogenic conformational transition induced by other sugar species, when Na^+^ is absent. k_obs_ is independent of sugar concentrations within the tested concentration ranges, but it depends on the sugar species: D-glucose (94 s^−1^) > D-galactose (84 s^−1^) > MDG (74 s^−1^) > D-xylose (37 s^−1^) ≈ OMG (36 s^−1^). This demonstrates again that the sugar-induced conformational transition is faster for D-glucose compared to the minor substrates. Since the transport I_max_ of OMG is 2.3 times larger compared to D-glucose (Table 1) and OMG triggers a slower electrogenic conformational transition within the loaded carrier at the same time, this transition may become rate limiting during Na^+^/sugar cotransport of minor substrates.

#### 3.2.5 Pre-steady-state charge translocation induced by different sugar substrates

Beside affinities and rate constants, PSS currents also reveal information about how the PSS charge translocations (Q_max_) compare across different sugar species (Table 1). Variations in Q_max_ indicate different conformational states after binding of different sugar species, as discussed for the H^+^/D-xylose cotransporter XylE (Bazzone et al., 2022b).

Q_max_ for the PSS current is determined *via* hyperbolic fit of the current integral. In the absence of Na^+^, the total current integral is used, since only PSS currents are recorded. In the presence of Na^+^, we integrated the first 30 ms of the current to approximate for the PSS charge translocation. However due to overlap with the transport current phase, the resulting values are only rough estimates.

In the absence of Na^+^, D-glucose (15 pC), D-galactose (18.3 pC), OMG (19.3 pC) and MDG (21.3 pC) induce similar charge translocations under saturating conditions. Q_max_ for D-xylose (>6 pC) was only estimated due to the high K_D,K_^app^. In the presence of Na^+^, Q_max_ is larger, but again similar for D-glucose (34 pC), D-galactose (39.2 pC), MDG (40.3 pC), OMG (45.6 pC) and D-xylose (>15 pC). These results indicate that the bound sugar species does not determine the conformational state of SGLT1.

### 3.3 Sugar translocation coupled to H^+^ and Li^+^ and the role of Cl^−^

It is known that SGLT1 is able to catalyze sugar symport coupled to Li^+^ and H^+^, but not to K^+^, Rb^+^, Cs^+^ or choline^+^ (Wright et al., 2011). We previously showed that in the presence of K^+^, Rb^+^ and choline^+^ no transport currents can be detected using SSME (Bazzone et al., 2022a). Here, we used Li^+^ and measurements at acidic pH to investigate sugar binding and transport coupled to Li^+^ and H^+^. Results are summarized in Table 2.

**TABLE 2:** Kinetic parameters obtained from transport and PSS currents using different cations and anions upon MDG concentration jumps.

	Cation		α-MDG				
	A	B	C	D	E	F	G
	K_M_	EC_50_^PSS^	K_M_	K_D_^app^	Transport I_max,t=50ms_	Transport I_max,rec_	PSS I_max,PSS_
Na^+^ + gluconate^-^	88 ± 8 mM Supplementary Figure S7	> 300 mM Supplementary Figure S7	6.8 ± 0.4 mM Supplementary Figure S7	189 ± 22 mM Supplementary Figure S7	448 ± 7 pA Supplementary Figure S7	500 ± 8 pA	2.97 ± 0.11 nA Supplementary Figure S7
Na^+^ + Cl^-^	49.5 ± 4 mM Figure 7/Supplementary Figure S7 (39–56 mM for D-glu Figure 3)	252 ± 48 mM Figure 7/Supplementary Figure S7 (231–285 mM for D-glu Figures 3, 5)	2.9 ± 0.2 mM Supplementary Figures S6, S7 (4.1 ± 0.3 mM Supplementary Figure S3)	96 ± 10 mM Supplementary Figures S6, S7 (32 ± 3 mM Supplementary Figure S4)	950 ± 20 pA Supplementary Figures S6, S7	1.53 ± 0.32 nA	5.32 ± 0.17 nA Supplementary Figures S6, S7
Li^+^ + Cl^-^	N.A.	> 300 mM Figure 7	137 ± 97 mM Supplementary Figure S6	191 ± 17 mM Supplementary Figure S6	97 ± 26 pA Supplementary Figure S6	N.A.	4.04 ± 0.13 nA Supplementary Figure S6
H^+^ + Cl^-^	0.32 µM (K^+^) 1.2 µM (Na^+^) Figure 7	0.5 μM (K^+^) 2 μm. (Na^+^) Figure 7	266 ± 99 mM Supplementary Figure S6	238 ± 32 mM Supplementary Figure S6	123 ± 22 pA (K^+^) Supplementary Figure S6	145 ± 17 pA (K^+^)	1.57 ± 0.08 nA (K^+^) Supplementary Figure S6
K^+^ + Cl^-^	—	—	—	262 ± 44 mM Supplementary Figure S6 (507 ± 383 mM for D-glu Figure 2)	25 pA* Supplementary Figure S6	—	2.53 ± 0.15 nA Supplementary Figure S6

All measurements were performed on 1 mm sensors. The different parameters shown (A-G) were determined in the presence of Na-gluconate, NaCl, LiCl, KCl at pH 5.4 (labeled H^+^), and KCl at pH 7.4 (labeled K^+^) to observe the effect of different cations and Cl^−^ on SGLT1 transport and binding kinetics. Current traces and fits are shown in figures of the main manuscript or supplementary figures as indicated. **(A)** K_M_ values for the cation were determined using hyperbolic fits of the currents 50 ms after MDG concentration jump as shown in Figure 3F for Na^+^ upon Na^+^/D-glucose cotransport. **(B)** EC_50_^PSS^ values for the cation were determined using hyperbolic fits of the PSS peak currents as shown in Figure 3H for Na^+^ upon D-glucose binding. **(C)** K_M_ values for MDG in the presence of the respective ions were determined using hyperbolic fits of the currents 50 ms after the MDG concentration jump as shown in Figure 1F for D-glucose in the presence of Na^+^. **(D)** K_D_^app^ values for MDG were determined using hyperbolic fits of the PSS peak currents as shown in Figure 1H for D-glucose binding to the Na^+^-bound carrier and Figure 2D for D-glucose binding to the empty carrier. Here, K_D_^app^ reflects the apparent affinity of Na^+^-, Li^+^-, and H^+^-bound SGLT1 and the empty carrier for MDG. **(E)** I_max,t=50ms_ is obtained from the same hyperbolic fit as the K_M_ in (C). It represents the maximum transport current obtained at saturating MDG concentrations. **(F)** I_max,rec_ was determined using hyperbolic fits of the steady-state current obtained from current reconstructions *via* circuit analysis as shown in Figure 1C for D-glucose. For LiCl no steady-state current is revealed *via* reconstruction due to the low amplitude of the transport current phase. **(G)** I_max,PSS_ is obtained from the same hyperbolic fit as the K_D_^app^ in (D). It represents the maximum PSS current obtained at saturating MDG concentrations. * In the presence of KCl at pH 7.4, no hyperbolic fit was possible due to the missing transport current phase. Instead of I_max_ the current at 500 mM MDG concentration is given, which is close to the noise level.

#### 3.3.1 Na^+^/sugar cotransport using α-methyl-D-glucose

For characterization of Li^+^ and H^+^/sugar cotransport, we used MDG instead of D-glucose as we anticipated reduced transport currents for Li^+^- and H^+^-coupled sugar translocation compared to Na^+^/sugar cotransport. MDG concentration jumps achieve higher transport I_max_ values (Table 1), therefore improving the signal-to-noise ratio for the transport current.

**FIGURE 7:**
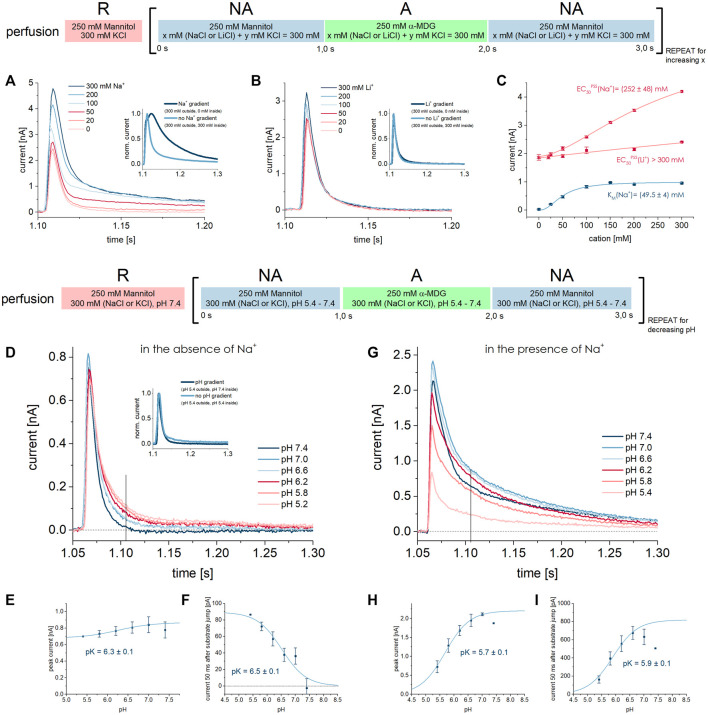
Effect of Na^+^, Li^+^, and H^+^ on PSS and transport currents. All current traces were recorded in the presence of different Na^+^, Li^+^ or H^+^ concentrations in all measurement solutions upon 250 mM MDG concentration jumps. Averaged data of *n* = 5 different sensors is shown. **(A)** Current traces were recorded at pH 7.4 in the presence of different Na^+^ concentrations in all measurement solutions. The inset compares the current trace where Na^+^ is only available in the external NA and A solutions and absent in the internal R solution (dark blue trace), leading to an inward directed Na^+^ gradient and consequently to an increased transport phase. **(B)** Same as (A) but with different Li^+^ concentrations as indicated. The inset shows that a Li^+^ gradient does not affect the current trace. **(C)** Li^+^ and Na^+^ dependent peak currents and currents 50 ms after substrate jump are fitted using a hyperbolic equation to derive EC_50_^PSS^ or K_M_ values, respectively. The PSS peak current observed in the Li^+^ dataset at 0 mM Li^+^ was normalized to the PSS peak current observed in the Na^+^ dataset at 0 mM Na^+^. The EC_50_^PSS^ for Li^+^ could only be estimated to be > 300 mM. **(D)** Current traces were recorded in the absence of Na^+^ at different pH values as indicated. The inset shows that a H^+^ gradient does not affect the current trace. The vertical line indicates the time point at which the transport current is measured. **(E)** PSS peak currents are fitted using the titration equation I(t) = I_max_/(1 + 10^^(pK-pH)^) to determine an apparent pK of 6.3 ± 0.1 for MDG binding in absence of Na^+^. **(F)** Currents 50 ms after substrate jump are fitted using the titration equation I(t) = I_max_/(1 + 10^^(pH-pK)^) to determine an apparent pK of 6.5 ± 0.1 for H^+^/MDG cotransport in absence of Na^+^. **(G)** Current traces were recorded in the presence of 300 mM Na^+^ at different pH values as indicated. The vertical line indicates the time point at which the transport current is measured. **(H)** PSS peak currents are fitted using the titration equation I(t) = I_max_/(1 + 10^^(pK-pH)^) to determine an apparent pK of 5.7 ± 0.1 for MDG binding in the presence of Na^+^. **(I)** Currents 50 ms after the substrate jump are fitted using the titration equation I(t) = I_max_/(1 + 10^^(pK-pH)^) to determine an apparent pK of 5.9 ± 0.1 for H^+^/MDG cotransport in the presence of Na^+^.

The Na^+^ concentration dependence upon 250 mM MDG jumps (Figure 7A) is similar compared to D-glucose (Figure 3B), but with a more pronounced transport phase, as expected. The EC_50_^PSS^ for Na^+^ in the presence of MDG is 252 ± 48 mM (Figure 7C), in good agreement with the value in the presence of D-glucose (285 ± 39 mM, Figure 3H). The K_M_ value for Na^+^ in the presence of MDG is 50 ± 4 mM (Figure 7C) and agrees with the K_M_ in the presence of D-glucose (52 ± 13 mM, Figure 3F).

We also applied a Na^+^ gradient by reducing the internal Na^+^ concentration to zero before activating SGLT1 in the presence of 300 mM Na^+^ (inset of Figure 7A). As shown before, the transport current phase increased 3-fold (Bazzone et al., 2022a). This confirms that the transport rate depends on the Na^+^ release step, as previously reported (Parent et al., 1992b; Loo et al., 2006).

#### 3.3.2 Li^+^/sugar cotransport is 10 times slower

When repeating the assay discussed in Section 3.3.1, but replacing Na^+^ with Li^+^, we observed current signals that behaved drastically different (Figure 7B): (1) there is no clearly visible transport current phase and no Li^+^ dependence of the slow current component; (2) the sugar-induced PSS peak current increases with increasing Li^+^ concentrations, but to a much lower extent than with Na^+^; (3) the EC_50_^PSS^ value for Li^+^ is >300 mM (Figure 7C) and likely much higher than for Na^+^ (252 ± 48 mM).

To potentially enhance a transport current below resolution, we applied a Li^+^ gradient by reducing the internal Li^+^ concentration to zero (inset of Figure 7B). An inward directed Li^+^ gradient did not increase the slow current component significantly. This indicates that cation release is not the rate limiting step in Li^+^/sugar cotransport and/or that Li^+^/sugar cotransport is below the resolution limit, hence at least 10 times slower than Na^+^/sugar cotransport. Hirayama et al. found that the K_M_ for Li^+^ is three times higher than for Na^+^ and that I_max_ for Li^+^/sugar cotransport is reduced to 80% compared to Na^+^/sugar cotransport (Hirayama et al., 1997). In addition, apparent affinity for Li^+^ was shown to be much more sensitive to membrane voltage, leading the authors to conclude that Li^+^-coupled transport might not be detectable at 0 mV (Hirayama et al., 1997).

#### 3.3.3 H^+^ and Na^+^ compete for the same binding site

It is long known that protons can rescue electrogenic sugar transport in SGLT1 when Na^+^ is not available (Hoshi et al., 1986; Hirayama et al., 1994). We have measured sugar-induced currents in SGLT1 in absence of Na^+^ at different pH values. While the peak current reflecting the PSS current component slightly decreases with acidification (Figures 7D,E), the slowly decaying transport current component increases when more protons are available (Figures 7D,F).

At acidic pH, when H^+^/sugar transport is saturated, transport I_max_ is about 8 times lower compared to Na^+^/MDG cotransport at saturating Na^+^ concentrations (Table 2). Hence, in our system H^+^-coupled transport appears slower than Na^+^-coupled transport. The corresponding relative transport rate observed in TEVC experiments is 1.7 (Longpré and Lapointe, 2011), and significantly lower compared to our result. Other authors described a relative I_max_ of 0.5 (Hirayama et al., 1994; Wright et al., 1994; Hirayama et al., 1997); they found that H^+^-coupled transport appeared faster than Na^+^-coupled transport, even at a low membrane voltage of −10 mV. Quick et al. found that replacing Na^+^ with H^+^ has no impact on I_max_ at −110 mV (Quick et al., 2001).

We used a titration equation to derive an apparent pK of proton binding to SGLT1 of 6.5 ± 0.1 from the pH dependence of the transport current 50 ms after the sugar jump (Figure 7F). We will refer to this value as pK^SS^ since the pK is derived from the (steady-state) transport current. It corresponds to an apparent affinity for H^+^ of 0.3 µM. In the literature we found pK values in the absence of Na^+^, which range from 5.2 (7 µM) (Hirayama et al., 1997; Quick et al., 2001) and 5.5 (3 µM) (Hirayama et al., 1994) to 5.7–6.0 (1–2 µM) (Wright et al., 1994), which is close to our result. When the PSS peak currents are used for analysis, we derive an apparent pK^PSS^ of 6.3 ± 0.1 (Figure 7E). Interestingly, the pH dependence of both PSS and transport currents yields similar pK values; hence, the H^+^ K_M_ for H^+^/sugar transport and the H^+^ EC_50_^PSS^ are essentially the same, in contrast to the Na^+^ K_M_ and Na^+^ EC_50_^PSS^ that differ by a factor of 5 (Figure 3).

Like a Li^+^ gradient, a pH gradient does not affect transport I_max_, indicating that H^+^ release is not rate limiting in H^+^/sugar cotransport (Figure 7D inset). Since the external H^+^ concentration clearly affects transport I_max_, the H^+^ binding step affects the rate limiting reaction under the given conditions.

In the presence of Na^+^, the pH dependence is more complex due to competition between Na^+^ and H^+^ for the same binding site. At alkaline pH Na^+^-bound transporters dominate. Here, the PSS peak current induced by 250 mM MDG is maximized and SGLT1 performs Na^+^/sugar cotransport, which is observed by the increased transport phase (Figure 7G). With acidification, the dominated transporter state is H^+^-bound. In the H^+^-bound state, 250 mM MDG generates lower PSS peak currents and lower transport currents at the same time. Lower transport currents may be explained by reduced I_max_ and/or increased sugar K_M_ in H^+^/sugar cotransport compared to Na^+^/sugar cotransport. Lower PSS peak currents may be a consequence of higher K_D_^app^ for MDG binding to the H^+^-bound state and/or a reduced PSS charge translocation upon MDG binding to H^+^-bound SGLT1. Both will be addressed within the next two sections, when the MDG concentration dependence during H^+^/sugar cotransport is investigated.

In the presence of Na^+^, the pK^PSS^ (5.7 ± 0.1) (Figure 7H) and pK^SS^ (5.9 ± 0.1) (Figure 7I) are both decreased compared to measurements in the absence of Na^+^, because more protons are required to saturate the common binding site due to competition with Na^+^.

#### 3.3.4 Cation dependent K_M_ and transport I_max_ for α-methyl-D-glucose

To understand why transport and PSS peak currents are affected when Na^+^ is replaced by Li^+^ or H^+^, we determined K_M_, K_D_^app^ and relative I_max_ values for transport and PSS currents (Supplementary Figure S6). We used MDG concentrations from 1 to 512 mM to activate SGLT1 in the presence of Na^+^, Li^+^, H^+^ and K^+^. To compare K_M_ and I_max_ values, we have analyzed the MDG-dependent currents about 50 ms after the sugar jump.

Transport currents are best resolved in the presence of Na^+^ with an I_max_ value of 950 pA. When Na^+^ is replaced by K^+^, no transport phase is observed (I ≈ 25 pA). In the presence of Li^+^ and H^+^ I_max_ values for the transport current are 10 and 8 times lower compared to Na^+^/MDG cotransport, 97 pA and 123 pA, respectively. This is close to the resolution limit of the technique.

The K_M_ value for MDG transport coupled to H^+^ is 266 mM and 92-fold higher compared to Na^+^/MDG cotransport. The cation dependence of sugar K_M_ also strongly depends on membrane voltage: in the H^+^-coupled transport mode, at −150 mV the K_M_ for MDG increases 25-fold (Hirayama et al., 1997); at −110 mV, K_M_ increases about 20-fold (Quick et al., 2001); at −50 mV it increases 45-fold (Hirayama et al., 1997) to 100-fold (Hirayama et al., 1994). These values correlate well with the 92-fold increase in K_M_ we observed at 0 mV.

For Li^+^/MDG cotransport we determined a K_M_ value for MDG of 137 mM which is 47-fold higher compared to Na^+^/MDG cotransport. This also approximates values found in the literature: the K_M_ for MDG increased 11-fold at −150 mV and 187-fold at −50 mV when Na^+^ is replaced with Li^+^ (Hirayama et al., 1997).

Taken together, the reduced transport current in H^+^- and Li^+^-coupled compared to Na^+^-coupled transport modes is caused by both reduced I_max_ and increased sugar K_M_, in agreement with literature.

#### 3.3.5 Cation-dependent K_D_^app^ and pre-steady-state I_max_ for α-methyl-D-glucose

K_D_^app^ values for MDG are derived from PSS peak currents. Apparent MDG affinity is highest in the presence of Na^+^ and decreases in the order: Na^+^ (96 mM) > Li^+^ (191 mM) > H^+^ (238 mM) > K^+^ (262 mM). The change in K_D_^app^ in the presence of Li^+^, H^+^ and K^+^ is minor. This shows that binding cooperativity between MDG and the cation is only observed in the presence of Na^+^; the affinity for MDG increases in the presence of Na^+^, but not as strikingly in the presence of Li^+^ or H^+^. In this series of experiments, the K_D_^app^ for MDG was higher compared to the analysis of the dataset in Table 1 and Supplementary Figure S4, possibly due to the combined effects of changing to a different sample batch, and a different range of concentrations used.

The cation species has a large effect on the PSS charge translocation, indicated by different I_max_ values for the sugar-induced PSS peak current (Table 2). PSS I_max_ values increase in the order: H^+^ (1.57 nA) < K^+^ or empty carrier (2.53 nA) < Li^+^ (4.04 nA) < Na^+^ (5.32 nA). Since Li^+^, H^+^ and Na^+^ are all monovalent cations, the differences within the PSS charge translocations may not be attributed to the charge of the bound cation before the sugar binds. Rather, it is a consequence of the bound cation dictating different conformational states upon sugar binding as also proposed for the H^+^/sugar transporter XylE (Bazzone et al., 2022b).

Taken together, the reduced PSS current in Li^+^ and H^+^/sugar cotransport compared to Na^+^/sugar cotransport is caused by both a higher K_D_^app^ value–due to the lack of cooperativity between Li^+^ or H^+^ and the sugar–and a reduced PSS electrogenicity (lower I_max_) when sugar binds to the Li^+^- or H^+^-bound carriers.

#### 3.3.6 Chloride affects all kinetic parameters during Na^+^/D-glucose cotransport

We performed SSME experiments in the presence and absence of Cl^−^, replacing it with gluconate and found a high impact of Cl^−^ on SGLT1 kinetics as discussed in Supplementary Results S2.3.1 (Table 2; Supplementary Figure S7). In brief, in the presence of Cl^−^ K_D,Na_^app^ and K_M_ values for the sugar are decreased, while the transport rate (I_max_) is increased. In addition, Cl^−^ affects the PSS charge translocation, indicating a large impact on the conformational state of SGLT1 upon sugar binding, similarly as described for the cations.

We also performed SSME experiments using different Cl^−^ concentrations, determining the K_M_ and EC_50_^PSS^ for Cl^−^ being 6.8 ± 2.8 mM and 21 ± 2 mM, respectively (Supplementary Results S2.3.2, Supplementary Figure S8).

### 3.4 The 11-state kinetic model

**FIGURE 8:**
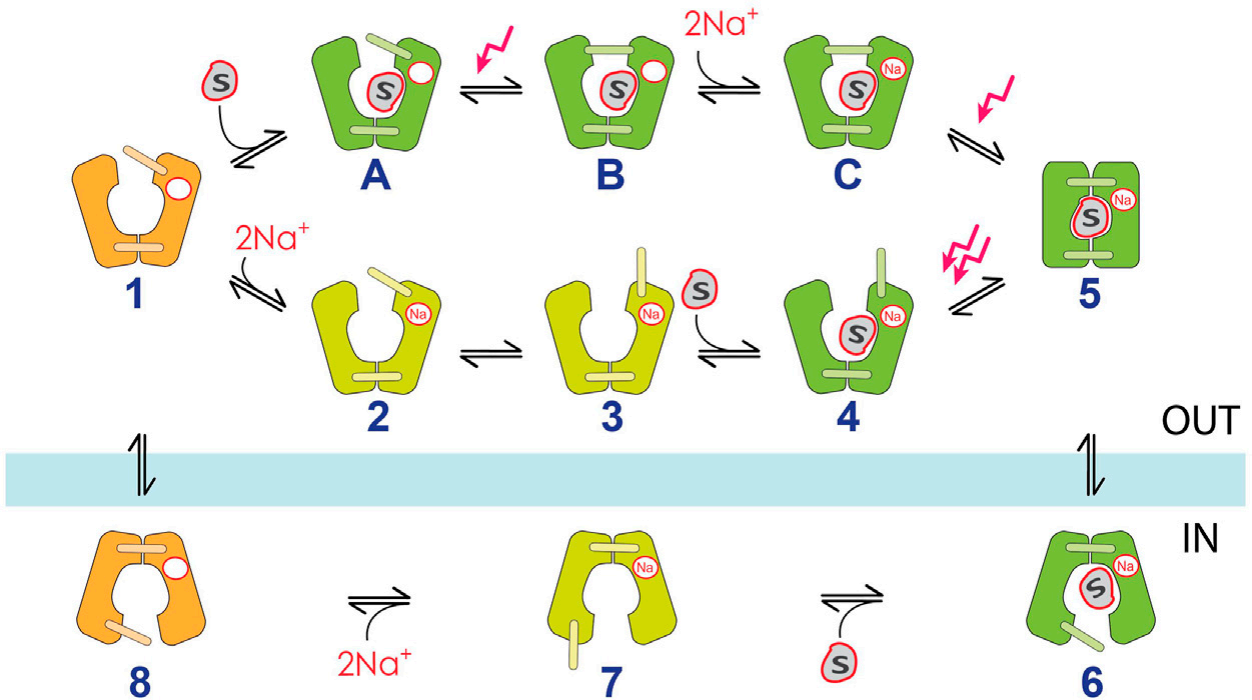
11-state kinetic model for Na^+^/D-glucose cotransport in SGLT1. Empty carrier conformations are shown in orange, SGLT1 conformations only bound to Na^+^ are shown in yellow and sugar-bound conformations are highlighted green. Inward-facing conformations are shown below the blue bar, outward-facing conformations are shown above. Rate and equilibrium constants used for the simulation of the model are provided in Supplementary Table S3. They were derived based on experimental data, calculations or literature. The states within the main transport cycle are labelled numerically from 1 to 8 and includes the ordered binding model when Na^+^ binds before the sugar (states 2–4, “Na^+^ first”). When sugar binds before Na^+^, an alternative pathway is followed, involving the states A–C (“sugar first”). Electrogenic steps are indicated as red arrows: the only required electrogenic reactions to simulate PSS and steady-state currents observed in SSME are related to substrate binding to the outward facing carrier. Within the “Na^+^ first” pathway, only the transition upon sugar binding to the Na^+^-bound carrier (4→5) is electrogenic. We therefore assume the total charge displacement for this transition being 2 elementary charges. Within the “sugar first” pathway, the transition upon sugar binding to the empty carrier (A→B) translocates 1.2 of an elementary charge, while the transition upon Na^+^ binding to the sugar-bound carrier (C→5) translocates 0.8 of an elementary charge as found previously (Bazzone et al., 2022a). The total transporter current is calculated by I = 1.2*([A]*kAB—[B]*kBA) + 0.8*([C]*kC5—[5]*k5C) + 2.0*([4]*k45—[5]*k54). Please note that the different conformational states within the substrate-bound carrier are illustrations that not necessarily represent the occurring conformational changes. For visualization purposes, we pictured the conformational states using different positions of the extracellular gate (states B and 3) and a major conformational transition to a fully occluded conformation (state 5). Electrogenic conformational transitions do represent local conformational transitions, which may rather follow an induced fit mechanism upon substrate binding, rearranging the local structure of the binding pockets, as proposed previously for H^+^-coupled sugar transporters (Bazzone et al., 2022b).

Based on the experimental results we developed an 11-state kinetic model (Figure 8). The model differs from previously described models (Loo et al., 2006; Longpré et al., 2012), mainly because these models are based on data derived from conventional electrophysiology which includes voltage triggered PSS currents that are attributed to steps within the empty carrier translocation. We discussed previously that we do not detect these PSS currents in SSME (Bazzone et al., 2022a), because SSME is based on substrate jumps. Hence, we simplified the empty carrier translocation into one single step (8→1).

The model considers a random binding order of Na^+^ and the sugar in the outward open carrier. This is required to explain the ability of the sugar to bind to the empty carrier and the different apparent affinities of the sugar to SGLT1 in the absence (Figure 2) and presence of Na^+^ (Figure 1/Figure 4). The outward open carrier (state 1) can either bind Na^+^ first (1→2), following sugar binding and alternating access–as assumed in previously proposed kinetic models–or it binds the sugar first (1→A), following Na^+^ binding and alternating access. The respective pathways will be referred to as “Na^+^ first” and “sugar first”.

The sugar-induced PSS current observed in SSME is attributed to an electrogenic reaction following sugar binding to the empty (1→A→B) or Na^+^-bound (3→4→5) carrier (Bazzone et al., 2022a). We also showed that Na^+^ binding triggers a PSS current when sugar was bound before (B→C→5), but not when Na^+^ binds to the empty carrier (1→2→3) (Bazzone et al., 2022a). Hence, we introduce 4 intermediate states within the transport cycle following the binding of each substrate within the outward facing carrier, accounting for one non-electrogenic and three electrogenic reactions. These reactions represent substrate-induced local conformational transitions within the carrier, displayed as different movements of the extracellular gate (Figure 8). However, they may be also attributed to substrate occlusion or transitions within the binding pocket potentially representing an induced fit mechanism. While the exact origin of the substrate-induced electrogenic events remains unclear, we do not observe electrogenic binding, as sugar binding is described to happen with a rate of 111.000 M^−1^s^−1^ (Longpré et al., 2012) or 45.000 M^−1^s^−1^ (Loo et al., 2008). Electrogenic binding is not consistent with our data, since (1) k_obs,PSS_ would depend on sugar concentration in a linear manner–not hyperbolic (Figure 4C)—and (2) k_obs_ would be 450 s^−1^ at 10 mM sugar and beyond the time resolution of the measurement (300 s^−1^), instead of 100 s^−1^ and even lower for other sugars (Supplementary Figure S4).

Upon substrate binding, the outward-facing substrate occluded state then undergoes alternating access to the inward-facing conformation (5→6). Since all experiments were carried out *via* substrate jumps from the extracellular side, only PSS currents representing transitions in the outward-facing carrier are observed. To simplify the model, we neglected similar electrogenic transitions and the random order substrate release in the inward facing carrier (6→7→8).

Many parameters within the model were established during SSME measurements. Other parameters were set based on existing models by Loo et al. (2006) and Longpré et al. (2012) and a few parameters were required to be set to a specific range for the simulation to match the experimental data for steady-state and/or PSS currents or to fulfill the law of detailed balance (Alberty, 2004). A comparison between experimental data and model output can be found in Supplementary Table S2, which is also discussed in Supplementary Results S2.4. An overview of all model parameters and their reasoning is shown in Supplementary Table S3.

## 4 Discussion

SGLT1 is a well-characterized model for Na^+^/sugar cotransport (Wright et al., 2011). However, information about substrate/transporter interactions are scarce due to the lack of technologies to measure sugar binding and the following conformational transitions directly. Sugar specificity for SGLT1 was analyzed using substrate jumps in TEVC experiments, but without visible sugar-induced PSS components, possibly due to low time resolution of the solution exchange (Kamitori et al., 2022). We recently showed that SSME opens a new perspective, being able to detect sugar binding and transport triggered by sugar concentration jumps in real-time and at high time resolution in one single assay (Bazzone et al., 2022a).

Based on this finding, we characterized the kinetics of the sugar translocation pathway for various sugar substrates and cations in SGLT1, concluding information about cooperativity, K_M_ and K_D_^app^ values and rate constants for the sugar-induced conformational transitions. We investigated the effects of sugar and cation species on the PSS charge translocation and relative sugar translocation rates. The data was used to develop a kinetic model to describe PSS and steady-state kinetics observed in SSME. The discussion will guide the reader through the different steps of the proposed kinetic model, presenting experimental evidence for the specific reactions.

### 4.1 Order of substrate binding and cooperativity between cation and sugar

It has long been known, that binding of external Na^+^ changes the conformation of the sugar-binding domain so that residues involved in sugar recognition are exposed to the external medium (Peerce and Wright, 1984; Hirayama et al., 2007). Na^+^ binding increases the open probability of the extracellular gate, enabling glucose to enter its binding site (Sala-Rabanal et al., 2012; Loo et al., 2013; Adelman et al., 2016; Gorraitz et al., 2017). Sugar binding then triggers closing of the extracellular gate to occlude the substrate from the external solution. The conclusion was a strict order of substrate binding, with Na^+^ binding first (Wright et al., 2011).

#### 4.1.1 Sugar binding occurs in the absence of Na^+^

While Na^+^ binding is not observed in SSME experiments (Bazzone et al., 2022a), sugar binding triggers an electrogenic conformational transition, allowing its detection. Interestingly, we detected sugar binding in the absence of Na^+^, clearly indicating that sugar can bind to the empty carrier and that Na^+^ binding is not a prerequisite for sugar binding (Figure 2). However, K_D_^app^ for the sugar is massively increased in the absence of Na^+^ (Figures 2D,E), consistent with previously described observations.

#### 4.1.2 K_M_ for Na^+^ depends on sugar concentration

Following the investigation of sugar binding in the absence of Na^+^, we studied cooperativity between Na^+^ and sugar binding. It was previously shown that K_M_ values for the sugar depend on external Na^+^ concentration and *vice versa* (Parent et al., 1992a; Wright et al., 2011). We confirmed that the K_M_ for the sugar decreases with increasing Na^+^ concentration, in agreement with Na^+^ binding before the sugar (Figure 5C). However, we see a similar effect in the opposite direction: the K_M_ for Na^+^ also decreases when higher sugar concentrations are used (Figure 5D). This clearly indicates that under transport conditions, a fraction of the transporter population binds sugar before Na^+^, supporting a random binding order. The K_M_ for Na^+^ decreases most for sugar concentrations between 1 and 20 mM (Figure 5D) and sugar concentrations in the small intestine may reach up to 50 mM (Ferraris et al., 1990). Hence, sugar binding to the empty carrier is relevant under physiological conditions. Using physiological Na^+^ and sugar concentrations, the 11-state model predicts that 4.1% of SGLT1 molecules use the ‘sugar first’ pathway.

#### 4.1.3 Cooperativity between Na^+^ and sugar has two root causes

Like K_M_ values, we found that K_D_^app^ values for Na^+^ and the sugar depend on the concentration of the respective co-substrate (Figure 5), indicating an allosteric binding mechanism. However, the decrease of K_D_^app^ is lower compared to the decrease of K_M_ when co-substrate concentrations increase. K_D_^app^ values for D-glucose increase 5.9-fold when the Na^+^ concentration is decreased from 300 mM to 20 mM. The increase of the respective K_M_ value is 55-fold (Figure 5C). We conclude that cooperativity between Na^+^ and the sugar is not only achieved by enhancing the sugar affinity, but also because Na^+^ binding improves the energy landscape for the conformational transitions leading to transport, adjusting the rate constants and consequently lowering the K_M_ below the K_D,Na_.

#### 4.1.4 Cooperativity is not observed when Na^+^ is replaced by H^+^ or Li^+^

For MDG we determined K_M_ values between 2.9 mM (Supplementary Figure S6) and 4.1 mM (Supplementary Figure S3) and K_D,Na_^app^ values between 32 mM (Supplementary Figure S4; Table 1) and 96 mM (Supplementary Figure S6; Table 2). Since the K_M_ is strikingly decreased compared to K_D,Na_^app^, MDG transport coupled to Na^+^ is kinetically driven - similar to D-glucose. In contrast, K_M_ values for MDG in the presence of H^+^ (266 mM) and Li^+^ (137 mM) are not decreased compared to the respective K_D_^app^ values (238 mM and 191 mM, respectively) and are also identical to the K_D,K_^app^ for MDG binding to the empty carrier (262 mM) (Table 2). Hence, Na^+^ binding—but not binding of H^+^ or Li^+^—causes a conformational state of SGLT1 that is required for efficient sugar binding and translocation. Since V_max_ is also dramatically reduced in Li^+^- and H^+^-coupled cotransport (about 90%, Figure 7 and Table 2), low K_M_ values and a high transport capacity are only achieved with Na^+^ as the co-ion. In addition, the missing binding cooperativity between H^+^ or Li^+^ and the sugar indicates a higher probability for the “sugar first” pathway in H^+^- or Li^+^-coupled sugar cotransport.

### 4.2 The road to real sugar affinities

In contrast to the vast amount of available steady-state data for SGLT1, experimental K_D_ values are scarce. Binding is more difficult to assess compared to substrate transport. A number of equilibrium techniques are available to assess binding of a ligand to a receptor (Ma et al., 2018), but they are not suitable for transporters because substrates are translocated. K_D_ values for transporter substrates may be derived from PSS measurements: substrate binding induces conformational transitions within the protein, which can be detected, e.g., using optical assays. But depending on the number of reaction steps between binding and the detected transition and depending on the rate constants of the individual reaction steps, the determined K_D_ might be an apparent K_D_ and significantly different from the real K_D_ (Johnson, 2019; Jarmoskaite et al., 2020).

#### 4.2.1 Sugar-induced pre-steady-state reveals K_D_ for sugar binding

The sugar-induced PSS current recorded in SSME is a direct real-time response to sugar binding. We proposed before it may represent an electrogenic induced fit of SGLT1 following sugar binding (Bazzone et al., 2022a). A similar model was applied for H^+^/sugar transporters (Bazzone et al., 2022b). We concluded that the EC_50_ value for the PSS current is very close to the real K_D_, hence defining it as K_D_^app^.

To establish how close the real sugar K_D_ and the experimentally determined K_D_^app^ are, we simulated PSS currents using the 11-state model. For the simulations we used the experimentally determined K_D_^app^ values as K_D_. From the simulated PSS currents we found EC_50_ values very close to the defined K_D_ values (Supplementary Table S2): (1) for sugar binding to the Na^+^-bound carrier we defined K_D_ = 26 mM as experimentally obtained *via* the analysis of concentration dependent k_obs_ values in the presence of Na^+^ (Figure 4C). We found an EC_50_ from the simulated PSS currents of 27 mM using the same analysis procedure. When peak currents are analyzed from the simulated PSS currents an EC_50_ of 20 mM is obtained, close to the experimentally determined value of 15.9 ± 1.2 mM (Figure 1I). (2) For sugar binding to the empty carrier we defined the K_D_ = 200 mM as experimentally observed (Figure 2). From the simulated PSS currents we obtained an EC_50_ of 183 mM. Both clearly demonstrate that the EC_50_ of the PSS current in fact may be used as a measure for K_D_.

#### 4.2.2 Comparison of K_D_ values with the literature

Experimentally determined K_M_ values are often equated with K_D_ values in order to perform kinetic simulations on SGLT1 (Loo et al., 2006; Loo et al., 2008). For vSGLT, sugar binding and transport were measured using radiolabeled galactose. Here, the determined K_D_^app^ value of 180 µM (Li et al., 2015) indeed equals the K_M_ for steady-state transport (158 µM) (Turk et al., 2000). In contrast, using SSME we found that for a given SGLT1 substrate, the K_D_^app^ value is always higher than the K_M_ value (Table 1).

Potential discrepancies between K_D_^app^ values determined by different techniques may originate from different read-outs. For DNA polymerase it was shown that initial weak binding of a nucleotide to an open state is followed by a conformational transition to a closed state, which yields an apparent K_D_ three orders of magnitude lower than the K_D_ of nucleotide binding (Johnson, 2019). SGLT1 and other transporters might be very similar: while the real K_D_ is hardly accessible during an experiment, the apparent K_D_ (or associated rate constants) determined using most techniques is a consequence of substrate occlusion or major conformational transitions upon alternating access. The detection is often limited to the equilibrium between the substrate-free, open; and the substrate-bound, closed transporter state, with optimum interaction between the substrate and the transporter leading to high apparent affinities or low K_D_^app^ values.

As for DNA polymerase, the real K_D_ for sugar binding to SGLT1 might be several orders of magnitude higher than the apparent K_D_ derived from equilibrium techniques. The real K_D_ may only be identified from kinetic measurements. In SSME, a fast reaction is measured in real-time (250 s^−1^, Figure 4), likely representing an induced fit mechanism - a fast substrate-induced, local conformational transition. This is in contrast to the formation of an occluded state which is believed to represent a high energy intermediate on the road to alternating access (Forrest et al., 2011) that was considered to be slow, with a rate of 50 s^−1^ (Loo et al., 2006) or 100 s^−1^ (Longpré et al., 2012).

#### 4.2.3 K_D_ values for cations cannot be determined, but EC_50_^PSS^ was calculated

Since Na^+^ binding to the empty carrier does not induce PSS currents in SSME, K_D_ values for Na^+^ are not accessible (Bazzone et al., 2022a). When examining the cation concentration dependence of the sugar-induced PSS current, we estimate an EC_50_^PSS^ for the cation instead. Within the 11-state model, the sugar-induced PSS reaction is separated by two steps from the Na^+^ binding. Hence, the sugar binding kinetics likely affects the cation dependence of the sugar-induced PSS current and a real Na^+^ K_D_ may not be derived. However, for Na^+^ a K_D_ value of 20 mM was determined experimentally using a thermodynamic approach (Loo et al., 2013) that was used for our model simulations.

### 4.3 The electrogenic induced fit detected *via* solid supported membrane-based electrophysiology upon sugar binding

Beside sugar affinity, the sugar-induced PSS current contains two more key information about the mechanism of sugar translocation in SGLT1. First, the impact of different sugar and cation substrates on the PSS charge translocation reveals the existence of different conformational states (Bazzone et al., 2022b). Second, rate constants for the underlying conformational transition can be derived from the current decay.

#### 4.3.1 The molecular origin of the sugar-induced electrogenic conformational transition

Sugar-induced PSS currents have been detected in SSME recordings of other sugar transporters, such as the Na^+^/melibiose transporter MelB (Ganea et al., 2011) and the H^+^/sugar transporters LacY (Garcia-Celma et al., 2010), FucP (Bazzone et al., 2016) and XylE (Bazzone et al., 2016; Bazzone et al., 2022b). We have shown previously that the sugar-induced PSS charge translocation within the H^+^-coupled sugar transporter XylE reflects an electrogenic conformational transition of the sugar-bound carrier, potentially an induced fit of SGLT1 upon sugar binding (Bazzone et al., 2022b). Since sugars carry no charge, different charge translocations must result from the movement of charged protein residues. This indicates that different charge translocations correlate with different conformational transporter states upon sugar binding, resulting from different degrees of movement of charged protein residues along the membrane axis. Interestingly, this charge movement does not seem to correlate with the movement of a single charged amino acid within the transporter, but rather represents a change in the surface potential of the transporter upon substrate binding. This is indicated by mutagenesis studies on the H^+^/sugar cotransporter LacY: fast electrogenic PSS currents have been observed in SSME recordings for all tested mutants unless the mutation completely blocks sugar binding (Gaiko et al., 2013). Similarly, voltage steps triggered PSS currents recorded on SGLT1 have not been attributed to the movement of single amino acids during empty carrier translocation, but to the movement of protein dipoles (Wright et al., 2011).

#### 4.3.2 The cation affects the conformational state of SGLT1 after sugar binding

A substantial PSS charge translocation upon binding of the major sugar substrates potentially reduces the energy barrier of the following conformational transition leading to alternating access of the binding sites, since a lower amount of charge needs to be transferred across the membrane. The alternating access was described to be rate limiting within the substrate translocation pathway for H^+^- (Madej et al., 2014) and Na^+^-coupled cotransporters (Forrest et al., 2011). Binding of the major substrates already transfers a fraction of charge across the membrane, possibly leading to a reduced energy barrier and thus faster translocation rates. In fact, binding of the major sugar substrate D-xylose generates the greatest PSS charge translocation in XylE (Bazzone et al., 2022b).

In contrast to the H^+^/xylose transporter XylE, we could not find major differences in Q_max_, induced by different sugar substrates in SGLT1 (Table 1, Supplementary Figures S4, S5). When Q_max_ does not depend on the sugar species, similar conformations may result upon binding of different sugar substrates. This is in agreement with the observation that different sugar substrates in SGLT1 show also very similar transport I_max_ values, since substrate specificity in SGLT1 is defined by K_M_ (Table 1).

On the other hand, the PSS charge translocation changes dramatically depending on the bound cation. The greatest PSS peak currents are observed for the Na^+^-bound carrier (100%), followed by the Li^+^-bound carrier (76%), the empty carrier (48%) and the H^+^-bound carrier (30%) (Table 2). The PSS charge translocation does not correlate with the charge of the cation, which is the same for Na^+^, Li^+,^ and H^+^. PSS charge translocation rather correlates with different local conformational transitions around the cation binding site, induced by the sugar. Upon sugar binding, cations and charged protein residues are shielded from the water environment to a different extent. This leads to different transporter states, depending on the type of cation, potentially comprising different energy levels which enhance or inhibit the following transition leading to cation/sugar cotransport. It might be energetically beneficial for cation/sugar cotransport, when the extent of cation shielding upon sugar binding is high (as for Na^+^), since the charge required to translocate the cation across the membrane barrier is reduced, leading to higher rates for cation/sugar translocation. This is in agreement with the drastically reduced V_max_ in Li^+^- and H^+^-coupled sugar cotransport (Table 2; Figure 7). Our results also match with previous conclusions that the type of cation determines the conformation of SGLT1 upon sugar binding and that this conformation mainly affects translocation rates (Hirayama et al., 1997). As a side note, the PSS charge translocation is also affected by chloride: In the presence of Na^+^ the PSS peak current is reduced to 56%, when chloride is removed (Table 2; Supplementary Figure S7), indicating that Cl^−^—in addition to the bound cation—plays a crucial role for achieving a favorable conformational state upon sugar binding.

#### 4.3.3 Rate constants for the electrogenic induced fit of SGLT1 upon sugar binding

From the decay time of the PSS currents, we have determined rate constants k_obs_ for the underlying electrogenic reaction (Figures 2B, 4B). In the presence of Na^+^, k_obs_ is sugar concentration dependent, allowing the derivation of k_on_ and k_off_ values using a model equation that was used for similar PSS currents previously (Garcia-Celma et al., 2010; Bazzone et al., 2022b). For D-glucose we determined forward and reverse rates for the electrogenic induced fit of 208 s^−1^ and 56 s^−1^, respectively (Figure 4C). In the absence of Na^+^ we found k_obs_ ≈ 90 s^−1^, independent of sugar concentration between 5 mM and 500 mM (Figure 2). The concentration dependence of both types of sugar-induced PSS currents is described by the 11-state kinetic model (Supplementary Results S2.4, Supplementary Table S2).

k_obs_ values in the presence of Na^+^ are similar for different sugar substrates (Supplementary Figure S4). In the absence of Na^+^ k_obs_ is between 35 s^−1^ and 95 s^−1^ (Table 1; Supplementary Figure S5). Moreover, k_obs_ values are not noticeably affected when Na^+^ is replaced by Li^+^ or H^+^ (Figure 7). Altogether, the kinetics of the sugar-induced conformational transition seems to vary in a very limited frame and seems to be faster than the transport rate of SGLT1 under all conditions tested, hence likely not rate limiting for steady-state transport.

### 4.4 Evidence for rate limiting steps during sugar translocation

Steady-state transport rates for SGLT1-mediated Na^+^/D-glucose translocation were independently reported to be 28 s^−1^ (Loo et al., 2006) and 35 s^−1^ (Longpré et al., 2012). For natural substrates and at −150 mV two different kinetic models assume the rate limiting steps to be either the voltage independent internal Na^+^ release with 5 s^−1^ (Loo et al., 2006) or the voltage independent empty carrier translocation with 59 s^−1^ (Longpré et al., 2012).

#### 4.4.1 Empty carrier translocation is the slowest step during Na^+^/D-glucose cotransport

To estimate the rate limiting step at 0 mV, we tested the transport rate in the presence and absence of 300 mM internal Na^+^. When internal Na^+^ is replaced by K^+^, the slowly decaying transport current—corresponding to the transport rate—increased by a factor of 3.5 (inset of Figure 7A), in agreement with our previous study (Bazzone et al., 2022a). This may indicate that Na^+^ release is the rate limiting step. However, the internal Na^+^ concentration also affects the equilibrium of the Na^+^ release step. At high Na^+^ concentrations, the concentration of the inward facing empty carrier and therefore the rate of the empty carrier translocation will be reduced. Thus, the alternating access of the empty carrier may be rate limiting as well. In our model we assumed the slowest rate constant being the empty carrier translocation with k_81_ = 15 s^−1^ as used by Loo et al. (2006), but a fast Na^+^ release step with k_78_ = 500 s^−1^.

This observation is only valid for Na^+^-coupled sugar transport, but not when Na^+^ is replaced by Li^+^ or H^+^. In Li^+^- and H^+^-coupled transport modes, V_max_ is decreased 10-fold compared to Na^+^/sugar cotransport (Figure 7; Table 2), and independent of the cation gradient (insets of Figures 7B,D). Hence, cation binding or the alternating access of the substrate-bound carrier becomes the rate limiting step in Li^+^- and H^+^-coupled transport modes at 0 mV.

#### 4.4.2 Induced fit of SGLT1 upon sugar binding might be rate limiting for minor substrates

For natural substrates the rate constant for the sugar translocation step or alternating access of the substrate-bound carrier was assumed to be 50 s^−1^ (Loo et al., 2006) and 100 s^−1^ (Longpré et al., 2012) and slightly faster than V_max_, hence not rate limiting. However, it was already shown that sugar translocation becomes rate limiting when substrates with lower turnover rates are used, as demonstrated for indican (Loo et al., 2008).

We found an indication of a rate limiting step in the sugar translocation pathway for transport of D-glucose and other natural sugars: Using SSME, all tested sugar substrates show higher transport rates than D-glucose (Table 1; Supplementary Figure S3), indicating that the rate for D-glucose translocation is limited compared to other substrates.

For the major substrates (D-glucose, MDG, D-galactose), as discussed before, the sugar-induced transition detected as a fast PSS current in SSME is unlikely to represent the rate limiting step during sugar translocation. First, k_obs_ for D-glucose under saturating conditions [≈250 s^−1^, Figure 4C and Table 1) is faster than the reported V_max_ values (28–35 s^−1^, (Loo et al., 2006; Longpré et al., 2012)]. Second, slower k_obs_ for the sugar-induced PSS reaction do not correlate with a reduced transport rate: the minor substrates (OMG, D-xylose) k_obs_ values are reduced 2-3-fold, while the transport I_max_ is increased up to 2-fold compared to the major substrates (Table 1, Supplementary Figures S3, S4). On the other hand, the induced fit of SGLT1 might become rate limiting for the minor substrates, because transport I_max_ is increased and k_obs_ for the induced fit is decreased.

#### 4.4.3 Substrate occlusion or alternating access is the slowest step in the sugar translocation pathway

For the major substrates with low transport I_max_ and high k_obs_, the rate limiting step within the sugar translocation pathway must be after the fast sugar-induced transition, but still within the sugar translocation pathway, since the transport rate–proportional to I_max_–depends on the sugar species (Table 1). We propose that the rate limiting reaction directly follows the sugar-induced electrogenic conformational transition we observe in SSME, and likely represents the sugar occlusion as previously proposed for the H^+^-coupled sugar transporter XylE (Bazzone et al., 2022b) or the alternating access of the substrate-bound carrier as illustrated in the 11-state model (Figure 8). In fact, alternating access of the substrate-bound carrier is believed to occur with a slow rate of 100 s^−1^ (Longpré et al., 2012) or 50 s^−1^ (Loo et al., 2006), which we adopted to use in our 11-state model (Figure 8, 5→6), thus defining it as the rate limiting step within the sugar translocation pathway for the major substrates.

We recently showed that substrates being transported with higher I_max_ exhibit lower apparent affinities (Bazzone et al., 2022b). In SGLT1 a similar observation was made: D-glucose holds the lowest K_D_^app^ and I_max_ values across all tested substrates, while D-xylose and OMG show high K_D_^app^ values and high transport rates. This observation can be explained by a simple energy landscape: when the energy level of the substrate-bound transporter is low (low K_D_, high affinity), the energy barrier for the subsequent conformational transition—which we assume to be the rate limiting step—is increased (lower V_max_). A transporter can increase transport capacity either by increasing V_max_ for substrate translocation (increasing the energy level of the substrate-bound outward-facing carrier, hence reducing the energy barrier for the subsequent transition) or by increasing substrate affinity (decreasing its energy level), not both. Similar observations were made for indican: compared to MDG a five times higher affinity and a 100-fold decreased rate for the alternating access was described (Loo et al., 2008). We therefore suppose the rate of the alternating access of the substrate-bound carrier is increased for the minor substrates (OMG, D-xylose) compared to D-glucose due to their higher K_D_^app^ values.

### 4.5 Substrate specificity determinants

Substrate specificity is usually defined by the ratio V_max_/K_M_ or k_cat_/K_M_ as commonly used for enzymes (Johnson, 2019). However, there is substrate specificity on the level of substrate recognition and binding that may be different compared to steady-state conditions, because co-substrates might affect substrate affinity and energy barriers for conformational transitions during steady-state to different extents. In order to understand the molecular mechanisms behind substrate specificity, parameters beyond K_M_ values may be considered.

#### 4.5.1 K_M_ defines substrate specificity over V_max_

The most frequently used parameters for assessing substrate specificity are K_M_ and V_max_ values. Transporters may increase their transport capacity for one substrate by either increasing the transport rate (V_max_) or increasing apparent affinity for transport (decreasing K_M_), as long as the physiological substrate concentration is not saturating. Hence, substrate specificity is indicated by an increased V_max_/K_M_ ratio.

V_max_/K_M_ is highest for D-glucose, but most interestingly D-glucose shows the lowest steady-state current (lowest V_max_), but highest apparent affinity (lowest K_M_) across all tested substrates, indicating that the determinant of sugar specificity in SGLT1 is given by low K_M_ values; Overall, I_max_ is less affected than K_M_ when different sugars are compared. I_max_ differs by a factor of 1.9, while K_M_ values differ 160-fold across all tested sugars. We conclude that evolution did not optimize the transport capacity of SGLT1 by increasing I_max_, but by increasing the apparent affinity for D-glucose.

#### 4.5.2 Substrate specificity is not a result of sugar affinity to the empty carrier, but of cooperativity with Na^+^

Interestingly, compared to the 160-fold difference in K_M_ between sugars with highest and lowest specificities, K_D,K_^app^ values among different sugar substrates—representing the affinity to the empty carrier—seems to differ by a much lower factor (Table 1). The exact factor could not be determined, since K_D,K_^app^ for D-xylose was only estimated to be > 500 mM. However, the factor across the remaining four substrates is only 4-fold. Thus, the affinity of the empty carrier for the sugar is not a crucial factor for substrate specificity.

K_D,Na_^app^ values—representing the affinity of the Na^+^-bound carrier for sugar—show larger differences when compared across sugar substrates; they differ by a factor of 23 (Table 1). The different sugar affinities in the presence and absence of Na^+^ for one specific sugar substrate is a result of different degrees of cooperativity (K_D,K_/K_D,Na_) between Na^+^ and sugar binding. The degree of cooperativity is maximized for the major substrates, indicating that the binding of Na^+^ to SGLT1 adjusts the sugar binding site in a way that favors binding to the major substrates.

As mentioned before, Na^+^ binding was shown to trigger the opening of the extracellular gate, leading to increased accessibility for the sugar and higher apparent affinities (Sala-Rabanal et al., 2012; Loo et al., 2013; Adelman et al., 2016; Gorraitz et al., 2017). However, if cooperativity is solely explained by the opening of the extracellular gate, it is unclear why the extent of cooperativity differs for different sugars. We propose that the observed cooperativity is also a result of Na^+^-induced local conformational transitions within the sugar binding pocket—rather than the opening of the extracellular gate only—leading to more favored binding interactions with D-glucose, but not with minor substrates.

#### 4.5.3 The effect of steady-state kinetics on substrate specificity

While cooperativity between Na^+^ and the sugar enhances the specificity for D-glucose on the level of binding affinity (Section 4.5.2), specificity for D-glucose is further enhanced on the level of apparent affinity as observed during steady-state transport. K_M_ values are decreased compared to K_D,Na_ values, specifically for D-glucose and the major substrates, not for minor substrates (Table 1). Reducing K_M_ below K_D,Na_ is achieved by an energetically optimized pathway for the sugar dependent conformational transitions. The highest K_D,Na_/K_M_ ratios are observed for the major substrates, indicating that the conformational transitions leading to sugar translocation are optimized around the interactions between SGLT1 and D-glucose across the translocation pathway. The energy landscape and thus the rate constants for the sugar dependent transitions were optimized for D-glucose, since the K_M_ is a consequence of the specific combination of rate constants within the transport cycle (Johnson, 2019).

#### 4.5.4 There are four substrate specificity determinants

In summary, there are four major mechanisms to adjust substrate specificity and enhance transport capacity: 1) increasing the sugar affinity for the empty carrier (K_D,K_), 2) increasing I_max_ values at the cost of lower sugar affinity, 3) enhancing sugar affinity to the Na^+^-bound carrier by increasing the degree of cooperativity (K_D,K_/K_D,Na_), and 4) enhancing the apparent sugar affinity by optimizing the kinetics of the sugar translocation pathway (K_M_). Improving the K_D,K_/K_M_ ratio throughout evolution reflects the major mechanism to generate substrate specificity in SGLT1 for D-glucose. The minor sugar substrates show virtually no improvement of K_M_ compared to K_D,K_. Transport of MDG and D-galactose is still possible with low K_M_ due to their structural similarities with D-glucose, allowing for effective binding cooperativity and an energetically optimized pathway for the sugar dependent conformational transitions, optimizing K_M_.

## 5 Conclusion

The sugar-induced PSS charge translocation opens a new perspective to analyze sugar binding and determine kinetic and thermodynamic parameters such as rate constants and real affinity values (K_D_). Estimating K_D_ values is important to understand mechanisms such as binding cooperativity, since K_M_ values do not relate in any way to the real binding affinity when complex transport mechanisms are investigated (Johnson, 2019).

Summarizing, the sugar-induced PSS charge translocation in SSME is a good read-out for sugar binding, also observed in other sugar transporters (Ganea et al., 2011; Bazzone et al., 2016; Bazzone et al., 2022b). The capability of detecting substrate binding in a label-free, real-time assay enhances the possibilities for functional transporter studies and fills the gap of knowledge about substrate binding kinetics.

Recently we described partially uncoupled modes in the H^+^/glucose transporter GlcP (Bazzone et al., 2017b). For SGLT1 we found a random binding order and several electrogenic conformational transitions within the sugar translocation pathway which were not reported before. These findings once again show that reality is more complex than expected and that simple kinetic models may never account for every possible event.

## Data Availability

The original contributions presented in the study are included in the article/Supplementary Material, further inquiries can be directed to the corresponding author.

## Glossary

### Abbreviations

A: activating solution
NA: nonactivating solution
R: resting solution
SSME: solid supported membrane-based electrophysiology
PSS: pre-steady-state
SS: steady-state
Glu: D-glucose
Gal: D-galactose
MDG: α-methyl-D-glucose
OMG: 3-O-methyl-D-glucose
Xyl: D-xylose
DOG: 2-desoxy-D-glucose
Fru: D-fructose

### Half saturation and equilibrium constants

K_M_: Michaelis Menten constant derived from transport related currents or charge translocation under steady-state or close-to steady-state conditions.
K_D_: dissociation constant, i.e., as used for model simulations.
K_D_^app^: apparent dissociation constant for the sugar, derived from sugar-induced PSS currents.
K_D,Na_^app^: **K_D_^app^** in the presence of 300 mM Na^+^.
K_D,K_^app^: **K_D_^app^** in the absence of Na^+^ (Na^+^ is replaced by 300 mM K^+^).
EC_50_: the empirical half saturation constant is used when values did not relate to either substrate transport (K_M_) or substrate binding induced transitions (K_D_^app^).
**EC_50_** ^PSS^: half saturation constant for the PSS current, as determined for the cation and Cl^−^ concentration dependence of the sugar-induced PSS current.

### Rate and time constants

τ_PSS_: decay time constant of PSS current phase from exponential fit.
τ_SS_: decay time constant of transport current phase from exponential fit.
k_obs_: observed rate constant associated with a current decay (SS or PSS phases) for a given sugar concentration. k_obs_ = 1/τ.
**k_on_** and **k_off_**: on- and off-rate constants for the sugar-induced electrogenic conformational transition in the presence of Na^+^, derived *via* model equation fit of concentration dependent k_obs_.

### Current and charge

I(t): time resolved current trace as recorded in SSME.
I_rec_(t): reconstructed transporter current using I(t), based on circuit analysis (Tadini-Buoninsegni and Fendler, 2015).
I_SS_: steady-state current obtained from I_rec_(t).
I_PSS_: PSS current. Either the peak current recorded from 1 mm sensors with high time resolution or the peak current from I_rec_(t).
I_max_: maximum current under saturating substrate concentration derived from a Hill fit of a concentration dependence. May be either of the PSS or the transport current component. I_max_ derived from the transport current correlates with the transport rate V_max_.
I_min_: minimum current derived from a modified Hill fit of the cation concentration dependent sugar-induced PSS current. For sugar concentration dependencies, I_min_ was always set to 0.
Q_max_: as I_max_, but here the translocated charge (peak current integral) was used for analysis.

## References

[B1] Adelman J. L. Ghezzi C. Bisignano P. Loo D. D. F. Choe S. Abramson J. (2016). Stochastic steps in secondary active sugar transport. Proc. Natl. Acad. Sci. U. S. A. 113, E3960–E3966. 10.1073/pnas.1525378113 27325773 PMC4941443

[B2] Alberty R. A. (2004). Principle of detailed balance in kinetics. J. Chem. Educ. 81, 1206. 10.1021/ed081p1206

[B3] Arthur S. Coon S. Kekuda R. Sundaram U. (2014). Regulation of sodium glucose co-transporter SGLT1 through altered glycosylation in the intestinal epithelial cells. Biochimica biophysica acta 1838, 1208–1214. 10.1016/j.bbamem.2014.01.002 24412219

[B4] Barfuss D. W. Schafer J. A. (1981). Differences in active and passive glucose transport along the proximal nephron. Am. J. physiology 241, F322–F332. 10.1152/ajprenal.1981.241.3.F322 7282931

[B5] Bazzone A. Barthmes M. Fendler K. (2017a). SSM-based electrophysiology for transporter research. Methods Enzym. 594, 31–83. 10.1016/bs.mie.2017.05.008 28779843

[B6] Bazzone A. Barthmes M. (2020). Functional characterization of SLC transporters using solid supported membranes. Methods Mol. Biol. Clift. N.J.) 2168, 73–103. 10.1007/978-1-0716-0724-4_4 33582988

[B7] Bazzone A. Körner A. Meincke M. Bhatt M. Dondapati S. Barthmes M. (2022a). SSM-based electrophysiology, a label-free real-time method reveals sugar binding & transport events in SGLT1. Biosens. Bioelectron. 197, 113763. 10.1016/j.bios.2021.113763 34768066

[B8] Bazzone A. Madej M. G. Kaback H. R. Fendler K. (2016). pH regulation of electrogenic sugar/H+ symport in MFS sugar permeases. PloS one 11, e0156392. 10.1371/journal.pone.0156392 27227677 PMC4882079

[B9] Bazzone A. Tesmer L. Kurt D. Kaback H. R. Fendler K. Madej M. G. (2022b). Investigation of sugar binding kinetics of the *E. coli* sugar/H+ symporter XylE using solid-supported membrane-based electrophysiology. J. Biol. Chem. 298, 101505. 10.1016/j.jbc.2021.101505 34929170 PMC8784342

[B10] Bazzone A. Zabadne A. J. Saliso wski A. Madej M. G. Fendler K. (2017b). A loose relationship: Incomplete H+/Sugar coupling in the MFS sugar transporter GlcP. Biophysical J. 113, 2736–2749. 10.1016/j.bpj.2017.09.038 PMC5770559 29262366

[B11] Birnir B. Loo D. D. Wright E. M. (1991). Voltage-clamp studies of the Na+/glucose cotransporter cloned from rabbit small intestine. Pflugers Archiv Eur. J. physiology 418, 79–85. 10.1007/BF00370455 2041729

[B12] Díez-Sampedro A. Lostao M. P. Wright E. M. Hirayama B. A. Diez-Sampedro A. (2000). Glycoside binding and translocation in Na(+)-dependent glucose cotransporters: Comparison of SGLT1 and SGLT3. J. Membr. Biol. 176, 111–117. 10.1007/s00232001081 10926676

[B13] Díez-Sampedro A. Wright E. M. Hirayama B. A. Diez-Sampedro A. (2001). Residue 457 controls sugar binding and transport in the Na(+)/glucose cotransporter. J. Biol. Chem. 276, 49188–49194. 10.1074/jbc.M108286200 11602601

[B14] Drew D. Boudker O. (2016). Shared molecular mechanisms of membrane transporters. Annu. Rev. Biochem. 85, 543–572. 10.1146/annurev-biochem-060815-014520 27023848

[B15] Eskandari S. Wright E. M. Loo D. D. F. (2005). Kinetics of the reverse mode of the Na+/glucose cotransporter. J. Membr. Biol. 204, 23–32. 10.1007/s00232-005-0743-x 16007500 PMC3000923

[B16] Ferraris R. P. Yasharpour S. Lloyd K. C. Mirzayan R. Diamond J. M. (1990). Luminal glucose concentrations in the gut under normal conditions. Am. J. physiology 259, G822–G837. 10.1152/ajpgi.1990.259.5.G822 2240224

[B17] Forrest L. R. Krämer R. Ziegler C. (2011). The structural basis of secondary active transport mechanisms. Biochimica biophysica acta 1807, 167–188. 10.1016/j.bbabio.2010.10.014 21029721

[B18] Forrest L. R. Rudnick G. (2009). The rocking bundle: A mechanism for ion-coupled solute flux by symmetrical transporters. Physiology 24, 377–386. 10.1152/physiol.00030.2009 19996368 PMC3012352

[B19] Forrest L. R. Zhang Y.-W. Jacobs M. T. Gesmonde J. Xie L. Honig B. H. (2008). Mechanism for alternating access in neurotransmitter transporters. Proc. Natl. Acad. Sci. U. S. A. 105, 10338–10343. 10.1073/pnas.0804659105 18647834 PMC2480614

[B20] Gaiko O. Bazzone A. Fendler K. Kaback H. R. (2013). Electrophysiological characterization of uncoupled mutants of LacY. Biochemistry 52, 8261–8266. 10.1021/bi4013269 24152072

[B21] Ganea C. Meyer-Lipp K. Lemonnier R. Krah A. Leblanc G. Fendler K. (2011). G117C MelB, a mutant melibiose permease with a changed conformational equilibrium. Biochimica biophysica acta 1808, 2508–2516. 10.1016/j.bbamem.2011.07.017 21801712

[B22] Garcia-Celma J. J. Ploch J. Smirnova I. Kaback H. R. Fendler K. (2010). Delineating electrogenic reactions during lactose/H+ symport. Biochemistry 49, 6115–6121. 10.1021/bi100492p 20568736 PMC2907097

[B23] Gerbeth-Kreul C. Pommereau A. Ruf S. Kane J. L. Kuntzweiler T. Hessler G. (2021). A solid supported membrane-based Technology for electrophysical screening of B0AT1-modulating compounds. SLAS Discov. Adv. life Sci. R D 26, 783–797. 10.1177/24725552211011180 33955247

[B24] Ghezzi C. Loo D. D. F. Wright E. M. (2018). Physiology of renal glucose handling via SGLT1, SGLT2 and GLUT2. Diabetologia 61, 2087–2097. –2097. 10.1007/s00125-018-4656-5 30132032 PMC6133168

[B25] Gorraitz E. Hirayama B. A. Paz A. Wright E. M. Loo D. D. F. (2017). Active site voltage clamp fluorometry of the sodium glucose cotransporter hSGLT1. Proc. Natl. Acad. Sci. U. S. A. 114, E9980–E9988. 10.1073/pnas.1713899114 29087341 PMC5699082

[B26] Hager K. Hazama A. Kwon H. M. Loo D. D. Handler J. S. Wright E. M. (1995). Kinetics and specificity of the renal Na+/myo-inositol cotransporter expressed in Xenopus oocytes. J. Membr. Biol. 143, 103–113. 10.1007/BF00234656 7537337

[B27] Han L. Qu Q. Aydin D. Panova O. Robertson M. J. Xu Y. (2022). Structure and mechanism of the SGLT family of glucose transporters. Nature 601, 274–279. 10.1038/s41586-021-04211-w 34880492 PMC9482448

[B28] Hirayama B. A. Loo D. D. F. Díez-Sampedro A. Leung D. W. Meinild A.-K. Lai-Bing M. (2007). Sodium-dependent reorganization of the sugar-binding site of SGLT1. Biochemistry 46, 13391–13406. 10.1021/bi701562k 17960916

[B29] Hirayama B. A. Loo D. D. Wright E. M. (1997). Cation effects on protein conformation and transport in the Na+/glucose cotransporter. J. Biol. Chem. 272, 2110–2115. 10.1074/jbc.272.4.2110 8999910

[B30] Hirayama B. A. Loo D. D. Wright E. M. (1994). Protons drive sugar transport through the Na+/glucose cotransporter (SGLT1). J. Biol. Chem. 269, 21407–21410. 10.1016/s0021-9258(17)31817-3 8063771

[B31] Hirayama B. A. Lostao M. P. Panayotova-Heiermann M. Loo D. D. Turk E. Wright E. M. (1996). Kinetic and specificity differences between rat, human, and rabbit Na+-glucose cotransporters (SGLT-1). Am. J. physiology 270, G919–G926. 10.1152/ajpgi.1996.270.6.G919 8764197

[B32] Hoshi T. Takuwa N. Abe M. Tajima A. (1986). Hydrogen ion-coupled transport of d-glucose by phlorizin-sensitive sugar carrier in intestinal brush-border membranes. Biochimica Biophysica Acta (BBA) - Biomembr. 861, 483–488. 10.1016/0005-2736(86)90458-x 3768358

[B33] Hummel C. S. Lu C. Loo D. D. F. Hirayama B. A. Voss A. A. Wright E. M. (2011). Glucose transport by human renal Na+/D-glucose cotransporters SGLT1 and SGLT2. Am. J. physiology Cell physiology 300, C14–C21. 10.1152/ajpcell.00388.2010 PMC3023189 20980548

[B34] Jardetzky O. (1966). Simple allosteric model for membrane pumps. Nature 211, 969–970. 10.1038/211969a0 5968307

[B35] Jarmoskaite I. AlSadhan I. Vaidyanathan P. P. Herschlag D. (2020). How to measure and evaluate binding affinities. eLife 9, e57264. 10.7554/eLife.57264 32758356 PMC7452723

[B36] Johnson K. A. (2019). New standards for collecting and fitting steady state kinetic data. Beilstein J. Org. Chem. 15, 16–29. 10.3762/bjoc.15.2 30680035 PMC6334795

[B37] Kamitori K. Shirota M. Fujiwara Y. (2022). Structural basis of the selective sugar transport in sodium-glucose cotransporters. J. Mol. Biol. 434, 167464. 10.1016/j.jmb.2022.167464 35077764

[B38] Karpowich N. K. Wang D.-N. (2008). Structural biology. Symmetric transporters for asymmetric transport. Sci. (New York, N.Y.) 321, 781–782. 10.1126/science.1161495 PMC2630483 18687947

[B39] Kazmier K. Claxton D. P. Mchaourab H. S. (2017). Alternating access mechanisms of LeuT-fold transporters: Trailblazing towards the promised energy landscapes. Curr. Opin. Struct. Biol. 45, 100–108. 10.1016/j.sbi.2016.12.006 28040635 PMC5491374

[B40] Koepsell H. (2017). The Na+-D-glucose cotransporters SGLT1 and SGLT2 are targets for the treatment of diabetes and cancer. Pharmacol. Ther. 170, 148–165. 10.1016/j.pharmthera.2016.10.017 27773781

[B41] Krofchick D. Huntley S. A. Silverman M. (2004). Transition states of the high-affinity rabbit Na(+)/glucose cotransporter SGLT1 as determined from measurement and analysis of voltage-dependent charge movements. Am. J. physiology. Cell physiology 287, C46–C54. 10.1152/ajpcell.00008.2004 14973149

[B42] Li Z. Lee A. S. E. Bracher S. Jung H. Paz A. Kumar J. P. (2015). Identification of a second substrate-binding site in solute-sodium symporters. J. Biol. Chem. 290, 127–141. 10.1074/jbc.M114.584383 25398883 PMC4281715

[B43] Lolkema J. S. Slotboom D.-J. (2008). The major amino acid transporter superfamily has a similar core structure as Na+-galactose and Na+-leucine transporters. Mol. Membr. Biol. 25, 567–570. 10.1080/09687680802541177 19031293

[B44] Longpré J.-P. Lapointe J.-Y. (2011). Determination of the Na(+)/glucose cotransporter (SGLT1) turnover rate using the ion-trap technique. Biophysical J. 100, 52–59. 10.1016/j.bpj.2010.11.012 PMC3010014 21190656

[B45] Longpré J.-P. Sasseville L. J. Lapointe J.-Y. (2012). Simulated annealing reveals the kinetic activity of SGLT1, a member of the LeuT structural family. J. general physiology 140, 361–374. 10.1085/jgp.201210822 PMC3457693 23008432

[B46] Loo D. D. Eskandari S. Boorer K. J. Sarkar H. K. Wright E. M. (2000). Role of Cl-in electrogenic Na+-coupled cotransporters GAT1 and SGLT1. J. Biol. Chem. 275, 37414–37422. 10.1074/jbc.M007241200 10973981

[B47] Loo D. D. F. Hirayama B. A. Cha A. Bezanilla F. Wright E. M. (2005). Perturbation analysis of the voltage-sensitive conformational changes of the Na+/glucose cotransporter. J. general physiology 125, 13–36. 10.1085/jgp.200409150 PMC2217483 15596535

[B48] Loo D. D. F. Hirayama B. A. Karakossian M. H. Meinild A.-K. Wright E. M. (2006). Conformational dynamics of hSGLT1 during Na+/glucose cotransport. J. general physiology 128, 701–720. 10.1085/jgp.200609643 PMC2151600 17130520

[B49] Loo D. D. F. Hirayama B. A. Sala-Rabanal M. Wright E. M. (2008). How drugs interact with transporters: SGLT1 as a model. J. Membr. Biol. 223, 87–106. 10.1007/s00232-008-9116-6 18592293

[B50] Loo D. D. F. Jiang X. Gorraitz E. Hirayama B. A. Wright E. M. (2013). Functional identification and characterization of sodium binding sites in Na symporters. Proc. Natl. Acad. Sci. U. S. A. 110, E4557–E4566. 10.1073/pnas.1319218110 24191006 PMC3839715

[B51] Ma W. Yang L. He L. (2018). Overview of the detection methods for equilibrium dissociation constant KD of drug-receptor interaction. J. Pharm. analysis 8, 147–152. 10.1016/j.jpha.2018.05.001 PMC6004624 29922482

[B52] Madej M. G. Sun L. Yan N. Kaback H. R. (2014). Functional architecture of MFS D-glucose transporters. Proc. Natl. Acad. Sci. U. S. A. 111, E719–E727. 10.1073/pnas.1400336111 24550316 PMC3932877

[B53] Mitchell P. (1957). A general theory of membrane transport from studies of bacteria. Nature 180, 134–136. 10.1038/180134a0 13451664

[B54] Panayotova-Heiermann M. Loo D. D. Kong C. T. Lever J. E. Wright E. M. (1996). Sugar binding to Na+/glucose cotransporters is determined by the carboxyl-terminal half of the protein. J. Biol. Chem. 271, 10029–10034. 10.1074/jbc.271.17.10029 8626557

[B55] Panayotova-Heiermann M. Loo D. D. Wright E. M. (1995). Kinetics of steady-state currents and charge movements associated with the rat Na+/glucose cotransporter. J. Biol. Chem. 270, 27099–27105. 10.1074/jbc.270.45.27099 7592962

[B56] Parent L. Supplisson S. Loo D. D. Wright E. M. (1992b). Electrogenic properties of the cloned Na+/glucose cotransporter: II. A transport model under nonrapid equilibrium conditions. J. Membr. Biol. 125, 63–79. 10.1007/BF00235798 1294062

[B57] Parent L. Supplisson S. Loo D. D. Wright E. M. (1992a). Electrogenic properties of the cloned Na+/glucose cotransporter: I. Voltage-Clamp studies. J. Membr. Biol. 125, 49–62. 10.1007/BF00235797 1542106

[B58] Peerce B. E. Wright E. M. (1984). Conformational changes in the intestinal brush border sodium-glucose cotransporter labeled with fluorescein isothiocyanate. Proc. Natl. Acad. Sci. U. S. A. 81, 2223–2226. 10.1073/pnas.81.7.2223 6425830 PMC345470

[B59] Pintschovius J. Fendler K. Bamberg E. (1999). Charge translocation by the Na+/K+-ATPase investigated on solid supported membranes: Cytoplasmic cation binding and release. Biophysical J. 76, 827–836. 10.1016/S0006-3495(99)77246-2 PMC1300084 9929484

[B60] Quick M. Loo D. D. Wright E. M. (2001). Neutralization of a conserved amino acid residue in the human Na+/glucose transporter (hSGLT1) generates a glucose-gated H+ channel. J. Biol. Chem. 276, 1728–1734. 10.1074/jbc.M005521200 11024018

[B61] Sala-Rabanal M. Hirayama B. A. Loo D. D. F. Chaptal V. Abramson J. Wright E. M. (2012). Bridging the gap between structure and kinetics of human SGLT1. Am. J. physiology. Cell physiology 302, C1293–C1305. 10.1152/ajpcell.00397.2011 PMC3361952 22159082

[B62] Tadini-Buoninsegni F. Fendler K. (2015). “Recording of pump and transporter activity using solid-supported membranes (SSM-Based electrophysiology),” in Pumps, channels, and transporters Editors Clarke R. J. Khalid M. A. A. (Hoboken, NJ: John Wiley & Sons), 147–177.

[B63] Thomas N. E. Feng W. Henzler-Wildman K. A. (2021). A solid-supported membrane electrophysiology assay for efficient characterization of ion-coupled transport. J. Biol. Chem. 297, 101220. 10.1016/j.jbc.2021.101220 34562455 PMC8517846

[B64] Tsujihara K. Hongu M. Saito K. Inamasu M. Arakawa K. Oku A. (1996). Na(+)-glucose cotransporter inhibitors as antidiabetics. I. Synthesis and pharmacological properties of 4'-dehydroxyphlorizin derivatives based on a new concept. Chem. Pharm. Bull. 44, 1174–1180. 10.1248/cpb.44.1174 8814948

[B65] Turk E. Kim O. Le Coutre J. Whitelegge J. P. Eskandari S. Lam J. T. (2000). Molecular characterization of Vibrio parahaemolyticus vSGLT: A model for sodium-coupled sugar cotransporters. J. Biol. Chem. 275, 25711–25716. 10.1074/jbc.M003127200 10835424

[B66] Tyagi N. K. Kumar A. Goyal P. Pandey D. Siess W. Kinne R. K. H. (2007). D-Glucose-recognition and phlorizin-binding sites in human sodium/D-glucose cotransporter 1 (hSGLT1): A tryptophan scanning study. Biochemistry 46, 13616–13628. 10.1021/bi701193x 17983207

[B67] Weyand S. Shimamura T. Yajima S. Suzuki S.'i. Mirza O. Krusong K. (2008). Structure and molecular mechanism of a nucleobase-cation-symport-1 family transporter. Sci. (New York, N.Y.) 322, 709–713. 10.1126/science.1164440 PMC2885439 18927357

[B68] Wright E. M. Loo D. D. F. Hirayama B. A. (2011). Biology of human sodium glucose transporters. Physiol. Rev. 91, 733–794. 10.1152/physrev.00055.2009 21527736

[B69] Wright E. M. Loo D. D. Panayotova-Heiermann M. Lostao M. P. Hirayama B. H. Mackenzie B. (1994). Active' sugar transport in eukaryotes. J. Exp. Biol. 196, 197–212. 10.1242/jeb.196.1.197 7823022

[B70] Yoo O. Lee D.-H. (2006). Inhibition of sodium glucose cotransporter-I expressed in *Xenopus laevis* oocytes by 4-acetoxyscirpendiol from Cordyceps takaomantana (anamorph = Paecilomyces tenuipes). Med. Mycol. 44, 79–85. 10.1080/13693780500142379 16805097

