# Supplementary material for: Functional characterization of SGLT1 using SSM-based electrophysiology: Kinetics of sugar binding and translocation
Source: Front Physiol. 2023 Feb 7;14:1058583. doi: 10.3389/fphys.2023.1058583 (PMC9941201; doi:10.3389/fphys.2023.1058583)
Supplement: Supplementary file 1 [file DataSheet1.pdf]

Electrophysiological characterization of SGLT1 using SSM-based electrophysiology: kinetics of sugar binding and translocation

Running Title: Sugar-induced kinetics of SGLT1

Andre Bazzone*[1], Rocco Zerlotti[1,2], Maria Barthmes[1], Niels Fertig[1]

[1] Nanion Technologies GmbH, Ganghoferstr. 70a, 80339, Munich, Germany
[2] University of Regensburg

* andre.bazzone@nanion.de

Keywords: SGLT1, SLC transporter, pre-steady-state (PSS), kinetics, mechanism, membrane transport, binding assay, Solid supported membrane (SSM), SURFE^{2}R

Table of Contents

S1 Supplementary Methods – p. 2

S2 Supplementary Results and Discussion – p. 5

S3 Supplementary Figures – p. 10

S4 Supplementary Tables – p. 18

References – p. 21

S1. Supplementary Methods

S1.1. Sensor preparation and electrophysiological measurements

SSME was performed using the SURFE^{2}R N1 device (Nanion Technologies GmbH) as described in detail previously (Bazzone et al., 2017a; Bazzone and Barthmes, 2020).

SURFE^{2}R N1 sensors of different diameters (1 mm or 3 mm) were coated first with an alkanethiol layer, then with 1,2-diphytanoyl-sn-glycero-3-phosphocholine (DPhPC) to form the SSM in the presence of resting solution (R). The composition of R depends on the assay (see below). Aliquots of frozen membrane vesicles are thawed and diluted 1:10 in R. Following sonication using a tip sonicator (UP 50 H, Dr. Hielscher, equipped with MS 1 tip; 10 bursts, 20% amplitude, 0.5 s cycle time), 10 µl are added to each sensor. The membrane vesicles attach to the SSM on the sensor and form a coupled membrane system. After centrifugation of the sensors (2,000 g, 30 min), they are ready-to-use.

The SURFE^{2}R N1 performs a fast solution exchange providing the substrate to activate the transporters immobilized on the sensors. Measurements take place under continuous flow of solutions. In our standard protocol, three solutions are flushed across the sensor, each for 1 s and at a speed of 200 µl/s: (1) During the flow of non-activating solution (NA) a current baseline is recorded; (2) When switching to the sugar containing activating solution (A), SGLT1 is activated and a capacitive current is recorded. (3) At the end of the experiment, NA flow restores initial conditions. Multiple solution exchange experiments are carried out on the same sensor. The solution exchange protocol is provided in each figure as a perfusion scheme that contains the compositions of R, NA and A. In case multiple conditions are measured using the same sensor, the measurement sequence is indicated within the perfusion scheme. All currents shown are recorded during the flow of activating solution, starting with the solution exchange from NA to A.

S1.2. Solutions for electrophysiological measurements

The main buffer used to prepare the measurement solutions contained 30 mM Tris/HCl, 3 mM EDTA, 1 mM EGTA and 120 mM NMDG/SO_4. If not stated otherwise, the pH was titrated to 7.4 using KOH. When H^+/sugar cotransport was investigated (Figure 7), titration was performed to achieve the acidic pH values as indicated.

Depending on the experimental conditions, different cations and sugars were used in NA and A solutions. In our standard assay 300 mM NaCl is added to both, NA and A solutions. In addition, the NA solution contains 250 mM Mannitol and the A solution contains 250 mM D-glucose, i.e. SGLT1 was always activated via sugar concentration jumps. In sugar specificity experiments, D-glucose was replaced by either MDG, D-galactose, OMG, D-xylose, DOG or fructose. When different sugar concentrations are used, the A solution (250 mM sugar) is diluted using NA solution (250 mM Mannitol) to keep a total osmolarity of ~250 mOsm/L. For the sugar concentration dependence, sensors were prepared in solution containing 250 mM Mannitol and measurements were carried out in sequence from high to low sugar concentrations.

When different Na^+ concentrations are used, Na^+ was supplemented with K^+ to achieve a constant cation concentration of 300 mM. If not stated otherwise, measurement solutions without Na^+ contain 300 mM K^+ instead. In some experiments, Na^+ is replaced by Li^+ to investigate Li^+/sugar cotransport (Figure 7). When Cl^- concentrations are altered, Cl^- was supplemented by gluconate to achieve a constant anion concentration of 300 mM. Measurements in absence of Cl^- contain 300 mM gluconate.

In our standard assays, the R solution used for sample dilution and sensor preparation matches the NA solution, hence the internal composition of the vesicles matches the external composition before SGLT1 is activated via sugar concentration jump. For measurements in the presence of cation gradients (Figure 7), the resting solution contains 300 mM K^+ instead of Na^+ or Li^+. The same is true when a cation concentration sequence is measured. Here, measurements were carried out in sequence from low to high cation concentrations. Before the measurement, the sensor was incubated for 3 minutes at the given cation concentration, assuming that the internal cation concentration equilibrates as found previously (Bazzone et al., 2022a).

S1.3. Data normalization

Since current amplitudes vary between sample batches and sensors, usually every dataset recorded from a single sensor was normalized before averaging across sensors to calculate the mean and standard deviation.

When concentration dependent peak currents or integrals are analyzed, the same normalization procedure was followed. First, all values recorded from one sensor are normalized to the value obtained for the highest substrate concentration. Then all normalized datasets recorded from different sensors are averaged yielding mean values and standard deviations. In addition, the average of the absolute values for the highest substrate concentration was determined. In order to get an estimate of I_{max} and Q_{max}, the normalized averaged dataset was multiplied by this average value. As a consequence of the normalization, standard deviation of the average data point for the highest substrate concentration is zero and all hyperbolic fits were performed without weighting.

In order to compare I_{max} and Q_{max} between different sugar substrates, we have measured 500 mM concentration jumps of all substrates on the same sensor (Figure 6). This yields accurate relative values for the different substrates. Normalized average datasets for each substrate then were normalized to reflect the relative peak currents for the different sugar substrates at 500 mM concentration. Therefore, I_{max} and Q_{max} values provided in Table 1 may be directly compared across substrates.

S1.4. Separation of PSS and transport currents during data processing

In SSME, usually the peak current recorded during the A solution flow is used for analysis. In most cases, it correlates with the steady-state transport rate of the transporter. However, for some transporters also substrate-induced PSS currents were observed, potentially overlaying with the transport current phase (Garcia-Celma et al., 2010; Bazzone et al., 2022b; Mikušević et al., 2019; Bazzone et al., 2016; Bazzone et al., 2017b).

As shown previously, in case of SGLT1 and depending on the experimental conditions, the peak current may be affected by both, transport and PSS current phases (Bazzone et al., 2022a). Hence, we did not simply use the peak currents to analyze the transport properties, but different methods to separate the phases representing PSS current and transport current phases.

To analyze transport currents, we used three different approaches: (1) the total integral during A solution flow reflecting the charge translocation. Usually, the overall charge translocation is dominated by transport since PSS charge translocation often only transfers a fraction of an elementary charge per transporter. The integration method may be used for datasets recorded from 3 mm and 1 mm sensors. (2) the current obtained from 1 mm sensors about 50 ms after sugar addition. At this time point PSS

currents already decayed to zero; (3) the steady-state current which was obtained via reconstruction of the transporter current (Tadini-Buoninsegni and Fendler, 2015).

To analyze PSS currents, peak currents from 1 mm sensors were used. In contrast to 3 mm sensors, time resolution is increased (Bazzone et al., 2017a). Since most of the recorded PSS currents are fast, peak currents are dominated by PSS currents when 1 mm sensors are used. However, there may still be a remaining impact of the transport current phase. Alternatively, we also used the charge translocation within the first 30 ms upon sugar concentration jump, mainly being affected by PSS charge translocation. In absence of Na^+, we used both, peak currents and integrals to analyze PSS currents, since transport is not observed under these conditions.

S1.5. Data processing: EC_{50} values, including K_M and K_D^{app}

Despite different read-outs, the data processing to derive EC_{50} values for PSS and transport currents is the same. Measurements were performed on at least 3 to 5 different sensors. All different concentrations used for the analysis were measured on the same sensor.

Due to variances across sensors, values obtained from the same sensor are normalized to the value obtained for the highest concentration. Datasets then were averaged across sensors to obtain a standard deviation. In order to retain information about relative I_{max} or Q_{max} values the averaged, normalized datasets may be further processed (compare section S1.3).

Average values and standard deviation for each concentration are plotted and fitted using a standard Hill equation $I=I_{max}*c^n/(EC_{50}{}^n+c^n)$ to obtain EC_{50} and I_{max} values. If not stated otherwise, the Hill coefficient n was set free for the fit. In some cases the modified Hill equation $I = I_{max} + (I_{max} - I_{min})*c^n/(EC_{50}{}^n+c^n)$ was used to include I_{min} at 0 mM concentration. This is the case for the cation EC_{50} of the PSS current, since the presence of the cation alters the sugar-induced PSS currents but is not a requirement to detect sugar binding induced currents (Figure 3, Figure 7).

S1.6. Data processing: rate constants

The current decay of the transient currents may be fitted assuming an exponential decay. Depending if only PSS or transport and PSS current phases are visible, the mono-exponential equation $I = A1*exp(-t/\tau_{PSS})$ (Figure 2B) or the bi-exponential equation $I = A1*exp(-t/\tau_{PSS})+ A2*exp(-t/\tau_{SS})$ (Figure 4B) was used. In case of the bi-exponential equation, the slow time constant τ_{SS} was always assigned to the transport current phase and the fast time constant τ_{PSS} to the PSS current phase. The rate constant of the PSS reaction is derived from $k_{obs,PSS} = 1/\tau_{PSS}$.

k_{obs} values were determined from at least 3 to 5 different sensors and averaged to determine standard deviation. We found k_{obs} being sugar concentration dependent in the presence of Na^+ (Figure 4). Assuming that the electrogenic reaction directly follows sugar binding, the concentration dependent k_{obs} can be used to determine k_{on} and k_{off} of the electrogenic reaction and the K_D for sugar binding using a fit with the model equation $k_{obs,PSS} = k_{on}*c^n/(K_D{}^n+c^n) + k_{off}$ (Garcia-Celma et al., 2010). For this fit we fixed n=1, since only one sugar molecule binds to SGLT1.

S2. Supplementary Results and Discussion

S2.1. Symmetry of transport

We showed before that PSS currents dominate only in influx mode, while the peak currents in the efflux mode are dominated by the slow transport component (Bazzone et al., 2022a). Here we wanted to test if the transporter has similar steady-state properties in both transport directions by determining the K_M for D-glucose during Na^+/D-glucose efflux.

To perform efflux experiments with precisely defined internal substrate concentrations, vesicles are preloaded with the substrate via dilution and sonication, before adding them to the sensors (section S1.1). Consequently, for each substrate concentration, a different sensor was used. The standard deviation is increased upon averaging, due to missing normalization. However, we found K_M = 2.2 ± 1.6 mM and I_{max} = 761 ± 117 pA for D-glucose during Na^+/D-glucose efflux, using the current 50 ms after the sugar concentration jump (Figure SI-1A). As a control, we repeated the influx assay with the same substrate concentrations and reproduced K_M = 1.7 ± 0.1 mM and I_{max} = 885 ± 125 pA using the same analysis procedure (Figure SI-1B), indicating that there may be only a slight asymmetry between steady-state influx and efflux properties regarding D-glucose transport in SSME experiments.

For the efflux assay, we used 300 mM Na^+ in both, internal and external solutions at 0 mV and only the sugar concentration gradients as the driving force. Apparent affinities for MDG in Na^+/sugar efflux mode found in literature are usually higher and between 7 and 56 mM (Sauer et al., 2000; Eskandari et al., 2005; Quick et al., 2003), indicating a more prominent asymmetry. Since we found that the K_M for MDG in SSME experiments is higher than for D-glucose (Table 1) our data approaches the lower end of the spectrum of literature values.

S2.2. Ordered binding models

The apparent affinity for the sugar depends on the Na^+ concentration, indicating binding cooperativity. Na^+ enhances the accessibility of the sugar binding site, increasing the sugar affinity and indicating an ordered binding model as proposed in literature (Sala-Rabanal et al., 2012; Loo et al., 2013; Gorraitz et al., 2017; Adelman et al., 2016; Peerce and Wright, 1984; Hirayama et al., 2007). Assuming that Na^+ binds before the sugar, the Na^+ concentration dependent K_M values for D-glucose may be used to derive $K_{M,Na}{}^{\#}(S)$, the K_M value for D-glucose when the Na^+ binding sites of SGLT1 are saturated (Figure SI-2A), as previously shown for H^+/sugar cotransport (Bazzone et al., 2016). The determined $K_{M,Na}{}^{\#}(S)$ for D-glucose is 0.68 ± 0.07 mM and the Hill coefficient for Na^+ binding is n = 2.02 ± 0.02, as expected for the binding of two Na^+ ions. The quality of the model fit ($R^2 > 0.9999$) supports the ordered binding model.

On the other hand, the apparent affinity for Na^+ also depends on the sugar, indicating that binding cooperativity works in both directions. We applied the same model equation assuming that the sugar binds before Na^+. We determined $K_{M,S}{}^{\#}(Na^+)$ = 48 ± 12 mM and a Hill coefficient for sugar binding of n = 0.52 ± 0.13 (Figure SI-2B). The fit is not exceptionally good with R^2 = 0.98. However, the data demonstrates that the sugar is not only able to bind in absence of Na^+ (Figure 2), but also able to bind before Na^+ under transport conditions, which in response increases the apparent affinity for Na^+.

S2.3. The role of chloride in sugar binding and Na^+/sugar cotransport

Chloride is known to bind to SGLT1, increasing the K_M for Na^+, while not being transported (Loo et al., 2000). On the other hand, Loo et al. found that I_{max} for Na^+/sugar transport and K_M for MDG are relatively unaffected by Cl^-. Using SSME, we aimed to expand this knowledge by investigating the effect of Cl^- on sugar binding and on Na^+/sugar translocation triggered by sugar concentration jumps under 0 mV conditions.

S2.3.1 Chloride affects all kinetic parameters during Na^+/D-glucose cotransport

We measured Na^+/MDG cotransport using different MDG and Na^+ concentrations in the presence of ~300 mM Cl^- in both, internal (R) and external (NA,A) solutions. The internal solution is defined during sensor preparation (section S1.1). We then compared the results with the same experiment, but partially replacing Cl^- by gluconate$^-$ (internal solution: 15 mM Cl^- / 285 mM gluconate$^-$; external solution: 0 mM Cl^- / 300 mM gluconate$^-$) (Figure SI-7).

From the MDG concentration sequence we found that chloride affected almost all kinetic parameters: (1) The $K_{D,Na}^{app}$ and K_M for MDG both double when Cl^- concentration is reduced, from 96 mM to 189 mM and from 2.9 mM to 6.8 mM, respectively. Opposingly, the effect of Cl^- on K_M for MDG found by Loo et al. was only ~10% at -50 mV (Loo et al., 2000). Interestingly, the $K_{D,Na}^{app}/K_M$ ratio is almost independent of chloride, being 33 and 28 in this set of experiments and at high and low Cl^- concentrations, respectively. The effect on K_M likely represents a direct consequence of Cl^- affecting $K_{D,Na}^{app}$. (2) The transport I_{max} decreases from 950 pA to 450 pA when Cl^- concentration is reduced. Hence, Cl^- not only enhances sugar affinity, but also the transport rate. This is roughly in agreement with the findings of Loo et al., who observed a decrease in transport current of about 30 % when Cl^- was removed at 0 mV (Loo et al., 2000). (3) Finally, I_{max} of the PSS peak current increases from 3 nA to 5.3 nA at high Cl^- concentrations, indicating a different sugar-induced charge translocation when Cl^- has bound. It is rather unlikely that chloride contributes with its negative charge towards the overall charge displacement measured upon sugar binding, since this would lead to an overall reduced charge translocation at high chloride concentrations – the opposite of what we observed. Chloride rather affects the conformational state of SGLT1 upon sugar binding. This is similar to the observation that the charge of the cation (Na^+, Li^+, H^+) has no direct impact on I_{max} of the PSS current (section 3.3.5 of the main manuscript). Cations and Cl^- define the overall conformational state of SGLT1 after sugar binding.

Following the MDG concentration sequence, we checked the impact of Cl^- on K_M and EC_{50}^{PSS} for Na^+. Similar as for the sugar, the values decrease from EC_{50}^{PSS} > 300 mM and K_M = 87.5 mM at low chloride concentrations to EC_{50}^{PSS} = 252 mM and K_M = 49.5 mM at high Cl^- concentrations. This is also in strong agreement with Loo et al. who observed an increase in K_M for Na^+ from 41 mM at 106 mM Cl^- to 62 mM at 20 mM Cl^- concentration (Loo et al., 2000). Interestingly the PSS peak current increases in the presence of Cl^- by a factor of 1.7 ± 0.1, independent of Na^+ concentration, indicating the lack of binding cooperativity between Na^+ and Cl^-.

S2.3.2 Cl^- only binds to SGLT1 from the intracellular site

To determine K_M and EC_{50}^{PSS} values for Cl^- via a Cl^- concentration sequence, we used different Cl^-/gluconate ratios to vary the Cl^- concentration and activated SGLT1 via 250 mM MDG concentration jumps in the presence of 300 mM Na^+. In SSME, concentration sequences are usually performed sequentially on the same sensor. When attempting the Cl^- sequence, starting with vesicles equilibrated in 15 mM Cl^-, we could not see any effect on the currents upon stepwise Cl^- addition by changing the

Cl^- concentration of the external solution from 0 mM to 300 mM (Figure SI-8A). Cl^- could not affect SGLT1 activity when provided with the external solutions (NA,A). Recently, we showed that the vesicles used in SSME measurements contain SGLT1 in right-site-out orientation (Bazzone et al., 2022a). Hence, the results indicate, that Cl^- only binds to SGLT1 from the intracellular site. However, Loo et al. found effects of Cl^- when removing it from the external solution in TEVC experiments on SGLT1 expressing oocytes (Loo et al., 2000). This conflict might be a result of different Cl^- permeabilities between the oocytes plasma membrane in the presence of membrane voltage and the plasma membrane vesicles derived from CHO cells at 0 mV which were used in our SSME assays.

S2.3.3 K_M and EC_{50}^{PSS} for Cl^-

Given that external Cl^- does not affect SGLT1 activity (section S2.3.2), we preloaded vesicles with different Cl^- concentrations via sonication before sensor preparation and performed only one measurement using a single Cl^- concentration per sensor to determine K_M and EC_{50}^{PSS} values for Cl^-. The PSS and transport current phases change with Cl^- concentration (Figure SI-8B), as expected from MDG and Na^+ dependent measurements at high and low Cl^- (section S2.3.1). From this dataset we determined K_M and EC_{50}^{PSS} values for Cl^-. From the current 50 ms after substrate jumps we determined a K_M value for Cl^- of 6.8 ± 2.8 mM (Figure SI-8C). The EC_{50}^{PSS} value concluded from the PSS peak current is 21 ± 2 mM (Figure SI-8D). Loo et al. found K_M = 21 mM (Loo et al., 2000). The hyperbolic fits of the Cl^- concentration dependent transport and PSS currents suggest that both currents are massively decreased at 0 mM Cl^-, indicating that the residual transport activity and PSS electrogenicity of SGLT1 at 0 mM internal Cl^- is close to zero.

S2.4. Model Simulations

The following paragraphs aim to critically analyze the 11-state model and what aspects of the experimental data it is able to describe. Clearly, it does not describe all kinetic data available on SGLT1, which would require additional intermediate states within the empty carrier along with additional electrogenic steps observed in conventional electrophysiology and as discussed by Loo et al. and Longpré et al. (Loo et al., 2006; Longpré et al., 2012).

S2.4.1 The 11-state kinetic model explains substrate binding and steady-state kinetics found with SSME

For the simulations we aimed to explain substrate binding and steady-state kinetics within an allowed discrepancy of a factor of ~2 between the model simulation and the experimental data. The proposed set of rate constants for the 11-state model (Table SI-3) is able to explain the following experimental data found using SSME (compare also Table SI-2):

1. Steady-state kinetics for Na^+ and sugar
    - **K_M for Na^+** under saturating sugar conditions is between 39 mM and 59 mM in SSME and 31 mM in the model simulations.
    - **K_M for the sugar** under saturating Na^+ conditions is between 0.8 and 1.9 mM in SSME and 2 mM in the model simulations.
    - **Cooperativity between Na^+ and Sugar** represented by relative changes of K_M values with co-substrate concentration is essentially the same within the model and experimental data: our model simulations show that a random binding order using the given rate and equilibrium constants explain the dependence of co-substrate concentrations and K_M values very well (Table SI-2). The relative change of the K_M for D-glucose at 300 mM Na^+ compared to 100 mM, 50 mM

and 20 mM Na^+ is 2.9 ± 0.8, 9.4 ± 6 and 55 ± 39 (Figure 5) and 3.5, 9.9 and 34 for the experimental dataset and the model simulations, respectively. The relative change of the K_M for Na^+ at 250 mM D-glucose compared to 20 mM, 4 mM and 1 mM D-glucose is similarly well represented by the model: In the experiment we obtained ratios of 1.3 ± 0.1, 2.3 ± 0.3 and 3.6 ± 0.9 (Figure 5); the model generates ratios of 1.8, 2.5 and 2.9 respectively.

- **Impact of Na^+ gradients**: At 300 mM external and internal Na^+, removing Na^+ from the internal solution in model simulations increases the steady-state current 2.1-fold. In the experiment, steady-state currents are increased by a factor of 3.48 ± 0.58.
- **Sugar Efflux**: The K_M value for the sugar in efflux mode at saturating Na^+ conditions is slightly increased compared to the influx mode. We observed a K_M of 2.2 ± 1.7 mM in SSME and 5.5 mM in model simulations.

2. EC_{50} of the sugar-induced PSS (referred to as K_D^{app} for the sugar)
   - **$K_{D,Na}^{app}$ for D-glucose**: The sugar EC_{50} of the sugar-induced PSS in the presence of Na^+ is between 14.5 mM and 38 mM in SSME and 20 mM in the model simulations.
   - **$K_{D,K}^{app}$ for D-glucose**: The sugar EC_{50} of the sugar-induced PSS in absence of Na^+ is 210 ± 52 mM in SSME and 182 mM in the model simulations.
   - **EC_{50}^{PSS} for Na^+**: The Na^+ EC_{50} of the sugar-induced PSS is between 231 mM and 285 mM in SSME and 102 mM in the model simulations.
3. Rate constants for PSS currents
   - **Rates in the presence of Na^+**: For D-glucose we determined forward and reverse rates for the electrogenic induced fit of 208 s^{-1} and 56 s^{-1}, respectively (Figure 4C). Accordingly, we defined k_{on} and k_{off} values for the transition following sugar binding to the Na^+ bound carrier in the 11-state model with 200 s^{-1} and 60 s^{-1}, respectively. Simulation of the PSS currents yield rate constants matching with the experimentally observed constants (Table SI-2): At 250 mM and 8 mM D-glucose we measured k_{obs}=244 s^{-1} and 103 s^{-1}, respectively. From the simulations we obtained 191 s^{-1} and 92 s^{-1}.
   - **Rates in absence of Na^+**: k_{obs} of sugar-induced PSS current in absence of Na^+ is independent of sugar concentration within the tested concentration range (5-500 mM). In SSME k_{obs} for 500 mM and 15 mM D-glucose are 95 ± 18 s^{-1} and 85 ± 17 s^{-1}, respectively. Accordingly, we defined k_{on} = 100 s^{-1} for the transition following sugar binding to the empty carrier. We used a k_{off} of 57 s^{-1}, similar to the k_{off} observed in the presence of Na^+. The model correctly simulates that k_{obs} in absence of Na^+ is almost independent of sugar concentration within the tested concentration range, yielding a k_{obs} of 53 s^{-1} at 500 mM D-glucose and a k_{obs} of 44 s^{-1} at 15 mM D-glucose concentration.
   - **Na^+ induced PSS currents**: We do not observe PSS currents upon Na^+ jumps in absence of sugar in both, SSME experiments (Bazzone et al., 2022a) and model simulations. k_{obs} upon 60 mM Na^+ jumps in the presence of 250 mM sugar is 139 ± 20 s^{-1} (Bazzone et al., 2022a) and 167 s^{-1} in model simulations.

S2.4.2 What the model does not consider

Some kinetic properties of SGLT1 found via SSME are not fully described by the proposed model. Further modifications are required to take the following experimental results into account. This may be fine tuning of rate constants or additional intermediate states.

1. PSS peak currents are overestimated compared to the steady-state current. We applied a 2 ms time filter representing the time resolution of the solution exchange to accommodate for this.

However, the PSS to SS current ratio obtained from the model is still increased 3-fold compared to the respective ratio found with SSME, which is 4.38 ± 0.27.

2. Simulations in the presence of a 300 mM Na^+ gradient show that the transport rate increase by a factor of 2.1. Experimentally we observed a factor of 3.5 (inset of Figure 7A). However, the reason for the Na^+ gradient affecting the steady-state transport rate observed in the model is different: the steady-state current decreases when Na^+ concentration increases above 120 mM in both, external and internal solutions.

One major flaw of the model is the missing electrogenicity of the empty carrier translocation. Loo and Longpré et al. showed that the voltage induced PSS currents may be explained by different electrogenic steps within the empty carrier translocation (Longpré et al., 2012; Loo et al., 2006). When charge translocation is added for the empty carrier translocation (Figure 8, 8->1), the ratio between PSS and SS currents in the model can be adjusted to match the ratio observed in SSME experiments: the SS current increases according to the charge translocation set for the empty carrier translocation, while sugar-induced PSS currents are unaffected. However, including electrogenic empty carrier translocation will automatically lead to a PSS response upon Na^+ binding which we clearly lack in SSME experiments (Bazzone et al., 2022a). Therefore, we decided to omit electrogenicity for empty carrier translocation. An alternative solution would involve an additional, electroneutral rate limiting step between electrogenic empty carrier translocation and Na^+ binding. We did not follow up on this for simplicity reasons.

S3. Supplementary Figures

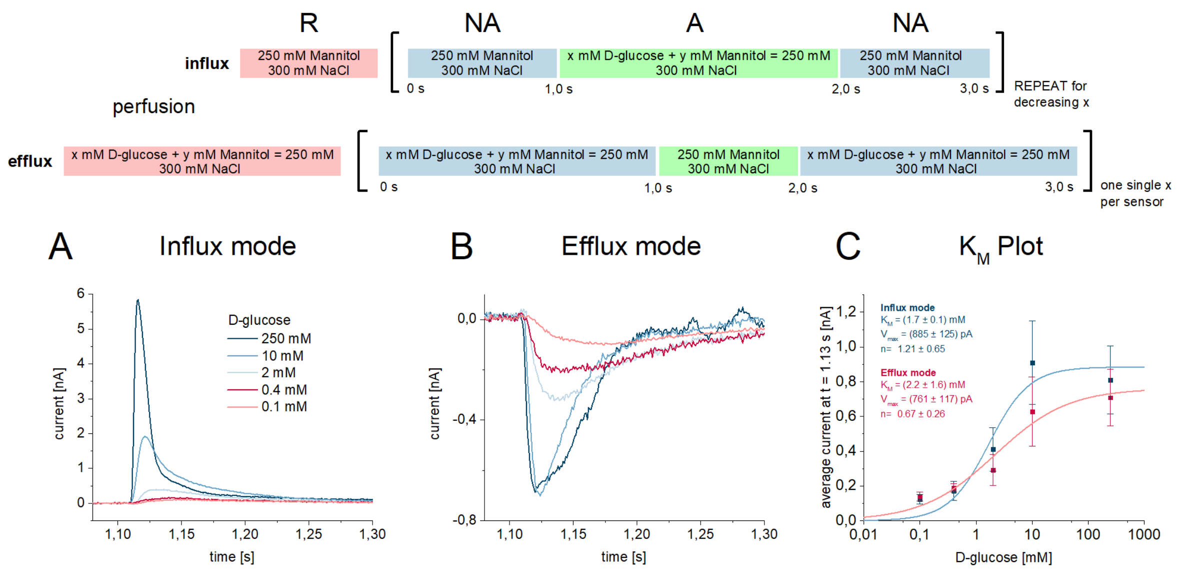

Figure SI-1: Comparison of K_M values for D-glucose during Na^+/D-glucose cotransport in influx and efflux modes. All current traces were recorded at pH 7.4 in the presence of 300 mM Na^+ in all measurement solutions and upon D-glucose concentration jumps between 0.1 mM and 250 mM. For the efflux assay, vesicles were preloaded with the respective concentration of D-glucose, during sensor preparation. Efflux was triggered by removing D-glucose from the sensor, generating an outward directed D-glucose concentration gradient. The influx assay was performed as described in the main manuscript. **A** Representative current traces recorded from one 1 mm sensor in influx mode. At higher D-glucose concentrations, the peak current is dominated by the sugar-induced PSS current. **B** Representative current traces recorded from different 1 mm sensors in efflux mode. The transport current dominates the peak at all concentrations tested. **C** Efflux and influx currents 50 ms after the sugar concentration jump are fitted using a hyperbolic equation to derive K_M values of 2.2 ± 1.6 mM and 1.7 ± 0.1 mM for D-glucose efflux and influx modes, respectively. In efflux mode, all concentration jumps are performed on an individual sensor and averaging occurs without normalization, leading to a somewhat higher standard deviation as shown in the plot. For the influx assay, measurements were performed on three different sensors, using the full concentration sequence each. As for the efflux data, averaging was performed without normalization, leading to higher standard deviation.

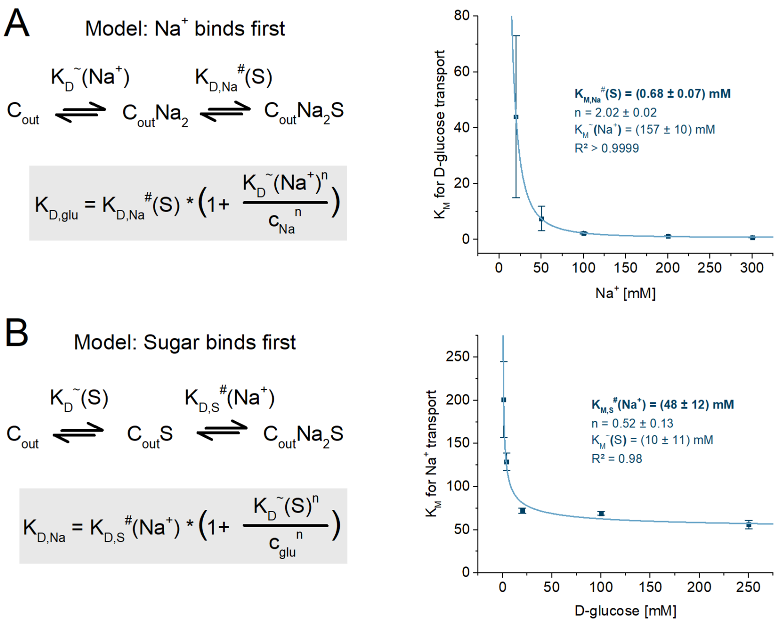

Figure SI-2: Fit of co-substrate concentration dependent K_M values using ordered binding models. For this analysis the dataset shown in Figure 5 was used. An ordered binding model was applied as demonstrated previously (Bazzone et al., 2016). The model equations were derived from the given ordered binding models, either assuming that the cation or the sugar binds first. The model equations consider the K_D values, e.g. real affinities for the substrates. Instead, we used the co-substrate concentration dependent K_M values due to the lack of K_D^{app} data for Na^+. Hence, the model fit rather represents the effect of the ordered binding on K_M, instead of K_D. We replaced K_D by K_M for this analysis since K_D and K_M were found to be proportional to each other, i.e. increasing K_D will also increase K_M (Table 1). $K_M^{\#}(X)$ reflects the K_M value for transport of substrate X, when 100% of the transporter population is bound to co-substrate Y. K_M approaches $K_M^{\#}(X)$ when the co-substrate Y is provided at oversaturating concentration. $K_M^{\sim}(Y)$ reflects the K_M value for transport of substrate Y, assuming it only binds to the empty carrier. **A** Model equation assuming that Na^+ binds before the sugar. Na^+ concentration dependent K_M values for D-glucose used for the fit were taken from Figure 5C. The model equation fits very well ($R^2 > 0.9999$) and yield a $K_M^{\#}$ for D-glucose of 0.68 ± 0.07 mM and a Hill coefficient of n = 2.02 ± 0.02, in agreement with two Na^+ ions binding to SGLT1 before D-glucose binds. **B** Model equation assuming that D-glucose binds before Na^+. The D-glucose concentration dependent K_M values for Na^+ used for the fit were taken from Figure 5D. The fit is not as good as for the model in A ($R^2 = 0.98$) but shows that D-glucose is able to bind before Na^+, at least to some extent and even under physiological concentrations. The fit yields a $K_M^{\#}$ for Na^+ of 48 ± 12 mM and a Hill coefficient of n = 0.52 ± 0.13.

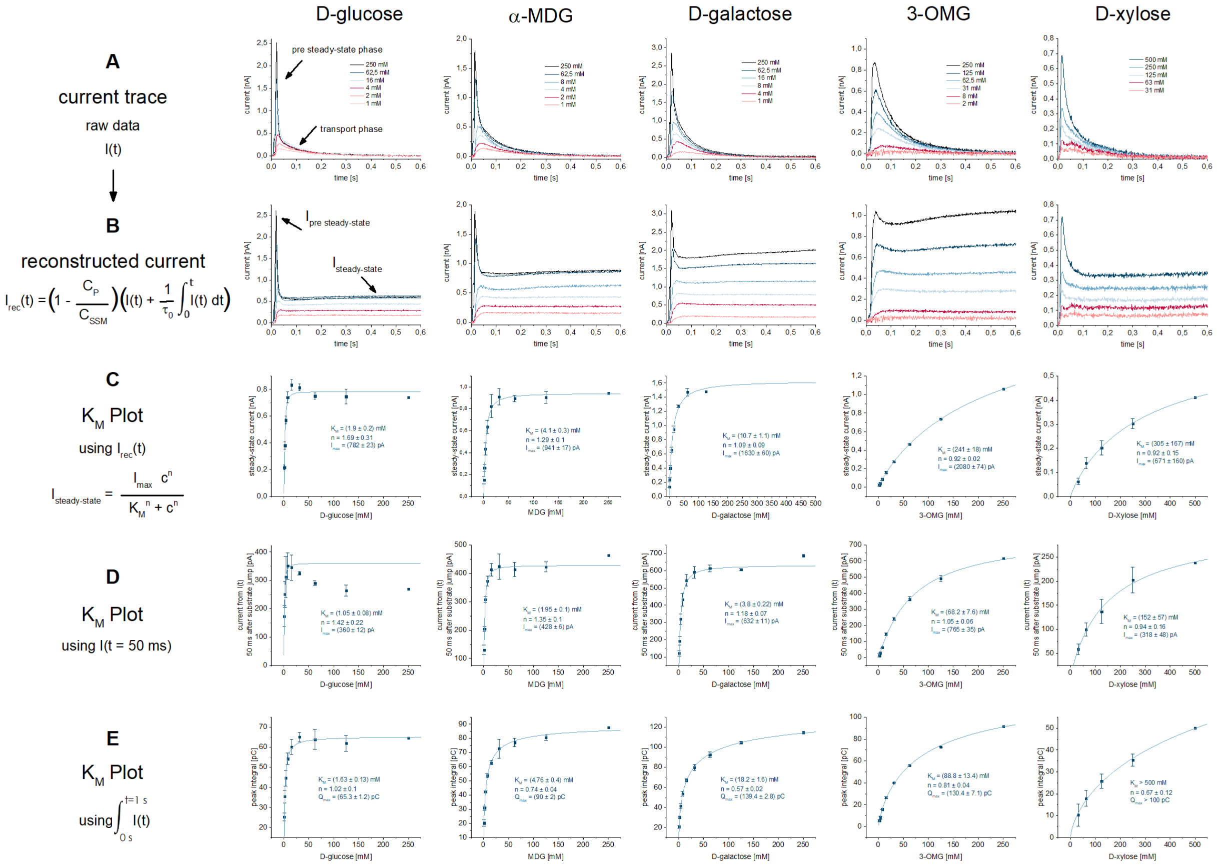

Figure SI-3: Comparison of current traces, reconstructed transporter currents and K_M plots for different sugar species during Na^+/D-glucose cotransport. The data reflects typical influx experiments in the presence of 300 mM Na^+ as shown in Figure 1 for D-glucose. All concentrations of the same sugar were applied to the same sensor. Each concentration dependence was repeated at least 5 times using different sensors. **A** Representative current traces recorded on a 1 mm sensor. **B** Via circuit analysis, transporter currents were reconstructed from the current traces shown in A (Tadini-Buoninsegni and Fendler, 2015). Steady-state currents are revealed from the transport phase of the original current, while the PSS current is essentially unaltered. **C-E** To obtain K_M values, datasets for the same sugar were normalized to the current or charge obtained for the highest sugar concentration, then averaged. Normalized, averaged datasets were then multiplied by the average current amplitude obtained for the highest concentration of the respective sugar within this set of experiments in order to include information about relative I_{max} for the different substrates (section S1.3). Fits using hyperbolic equations to derive K_M and relative I_{max} values for each sugar species were performed by using three different types of data from the same datasets. For the fits the following data was used. **C** Steady-state currents from the reconstructed transporter currents as shown in B; **D** Currents 50 ms after the sugar concentration jump from the current traces as shown in A; **E** The charge translocation (Q) obtained from the full current integral of the current traces shown in A. Details about the different analysis methods are provided in the main text.

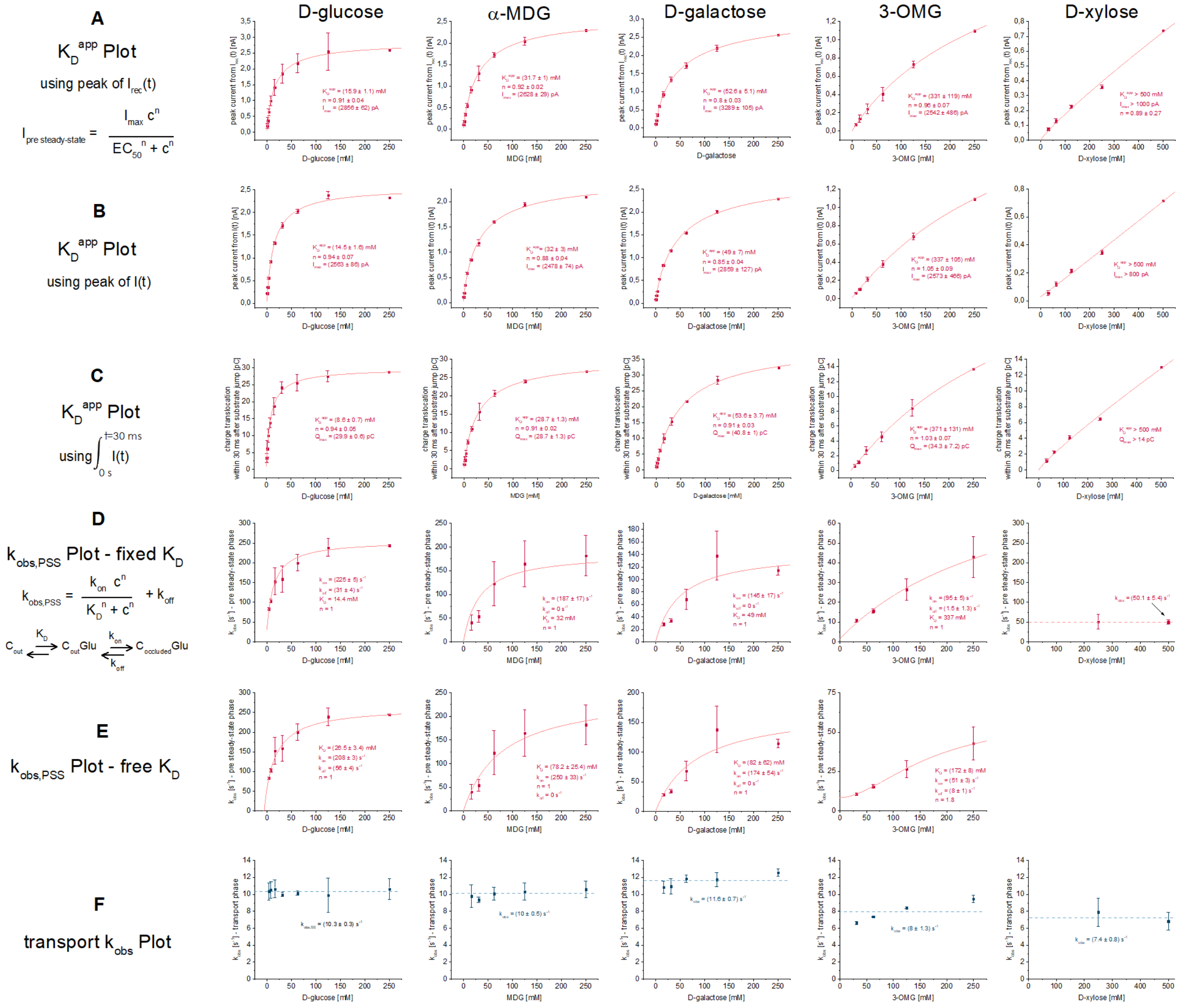

Figure SI-4: Comparison of $K_{D,Na}{}^{app}$ and k_{obs} plots for different sugar species during Na^+/D-glucose cotransport. The plots are based on the same datasets as the K_M plots shown in Figure SI-3 and followed the same averaging and normalization procedure. **A-C** Fits using hyperbolic equations to derive $K_{D,Na}{}^{app}$ and relative PSS I_{max} values for each sugar species were performed by using different types of data from the same datasets. For the fits the following data was used. **A** Peak currents derived from the reconstructed transporter currents as shown in Figure SI-3B; **B** Peak currents from the current traces as shown in Figure SI-3A; **C** The PSS charge translocation from the current integral within the first 30 ms upon sugar concentration jump. **D-E** Fits of the concentration dependent k_{obs} determined from the PSS current phase. The given model equation was used to derive k_{on} and k_{off} values for the electrogenic conformational transition upon sugar binding and the K_D values for the sugar. k_{obs} was determined via bi-exponential fit of the fast current decay as shown in Figure 4B for D-glucose. **D** To decrease the number of variables, the K_D value of the fitting equation was fixed to the one determined using the $K_{D,Na}{}^{app}$ plot shown in B. The Hill coefficient was fixed to n=1 to reflect the binding of one sugar molecule. For MDG and D-galactose a negative k_{off} resulted from the fit, hence k_{off} was fixed to 0 for these sugars. **E** Identical fit using the same dataset as in D, but with the K_D values as additional variables (not fixed). $K_D{}^{app}$ values obtained from the fits are similar to those obtained from the plots shown in A-C. For D-glucose and MDG a better estimate of k_{on} and k_{off} is achieved compared to D. **F** Plots of the concentration dependent k_{obs} determined from the transport current phase. The current decay is a consequence of the membrane being charged upon Na^+/sugar cotransport.

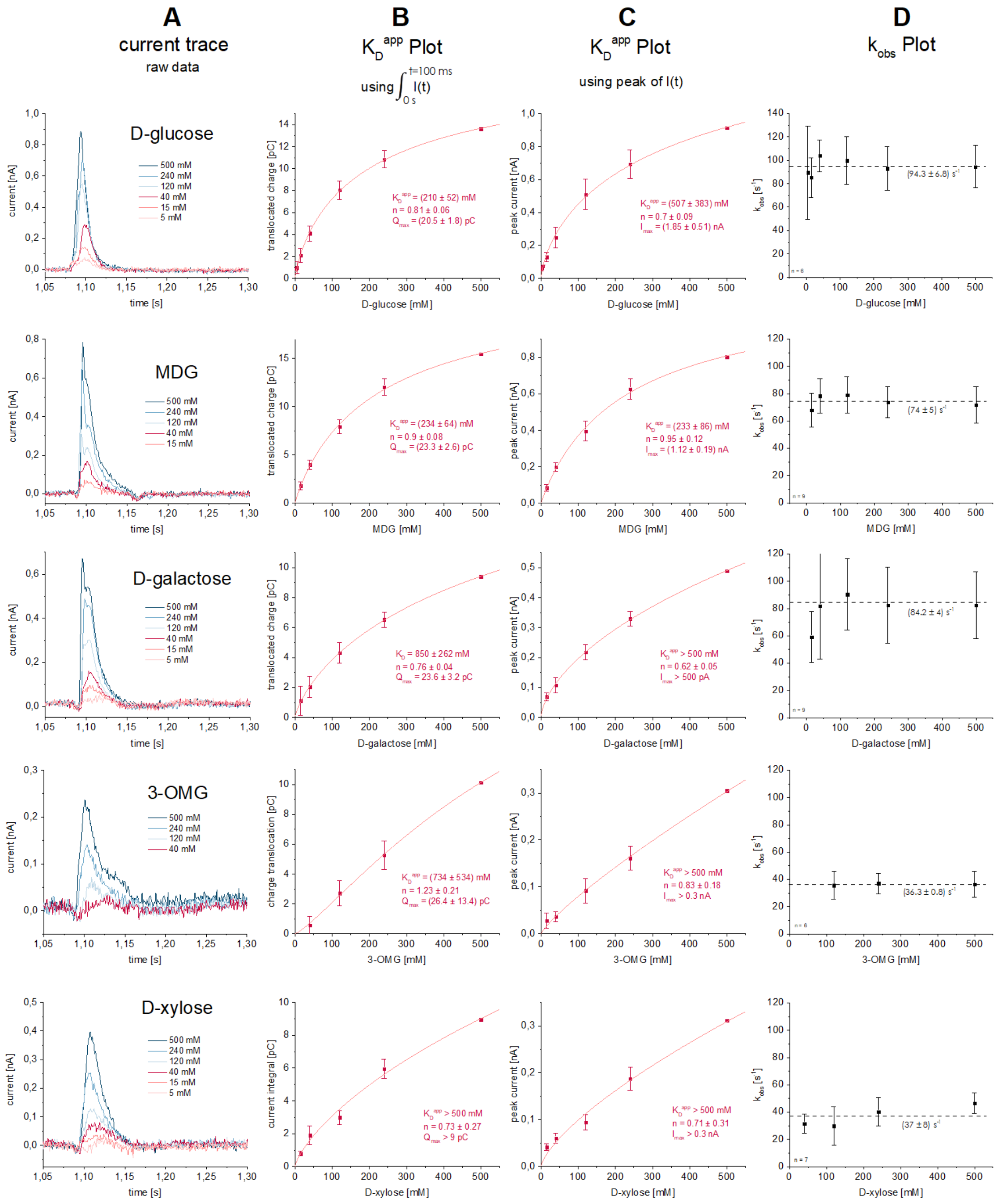

Figure SI-5: Comparison of current traces, K_D^{app} and k_{obs} plots for different sugar species upon sugar binding to the empty carrier. The data reflects typical sugar binding experiments in absence of Na^+ as shown in Figure 2 for D-glucose. All concentrations of the same sugar were applied to the same sensor. Each concentration dependence was repeated at least 5 times using different sensors. **A** Representative current traces recorded on a 1 mm sensor. **B-C** Fits using hyperbolic equations to derive $K_{D,K}^{app}$ and relative I_{max} or Q_{max} values for each sugar species were performed using the peak currents or the charge translocation (current integral), respectively. The data was averaged and normalized as described in Figure SI-3. **D** Plots of the concentration dependent k_{obs} determined via mono-exponential fit of the current decay as shown in Figure 2B for D-glucose. k_{obs} is independent from sugar concentration in the tested concentration range for all sugars.

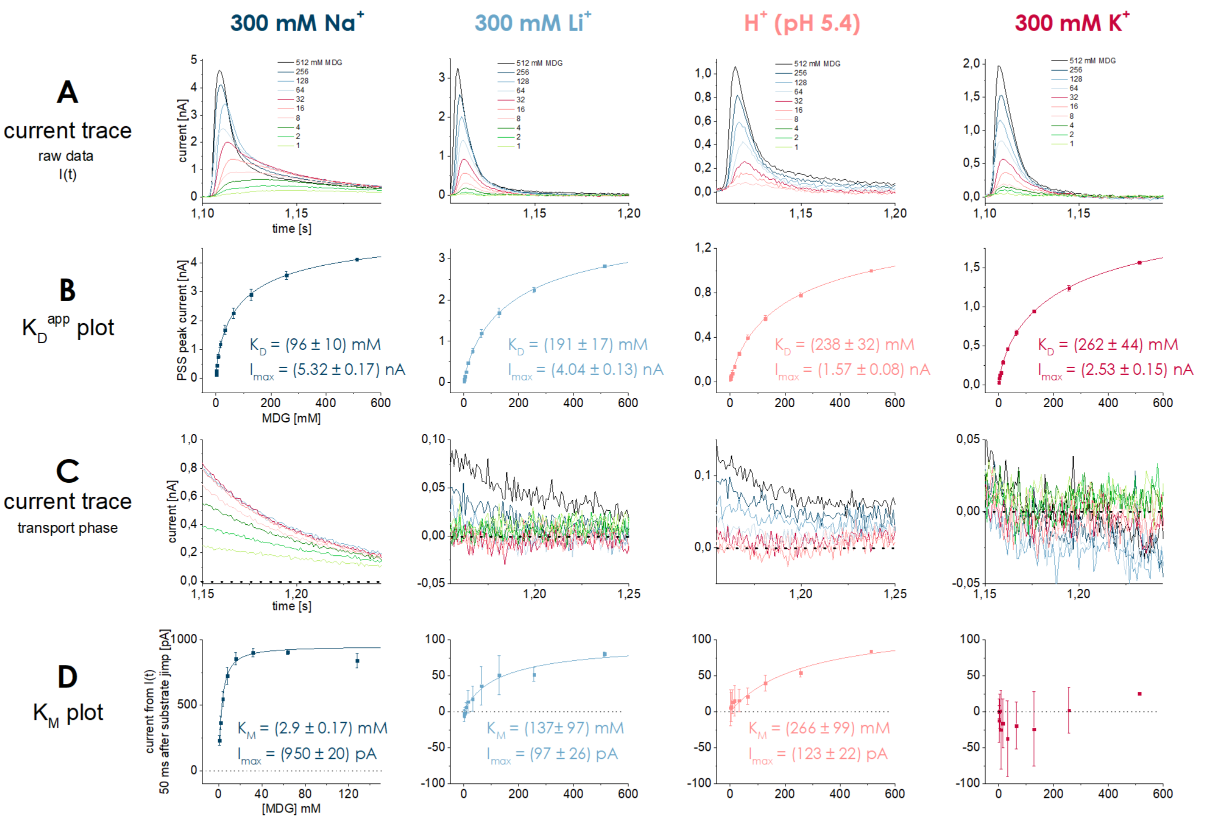

Figure SI-6: Effect of Na^+, Li^+ and H^+ on K_M and K_D^{app} for MDG during cation/MDG cotransport. MDG concentration jumps have been performed in the presence of different cations in all measurement solutions. Either 300 mM NaCl, 300 mM LiCl, 300 mM KCl at pH 7.4 or 300 mM KCl at pH 5.4 (labeled H^+) was used as the supplement in all measurement solutions as indicated. In the presence of Na^+, Li^+ and at acidic pH Na^+-, Li^+- and H^+-coupled MDG cotransport is measured. In the presence of K^+ at alkaline pH, only PSS currents are detected. Sensors were prepared in the presence of the respective cations and all MDG concentrations were applied to the same sensor. Data was averaged and normalized across at least 4 different sensors as described in Figure SI-3. **A** Representative current traces recorded on a 1 mm sensor for different MDG concentrations in the presence of different cations. The MDG concentrations are indicated. **B** Fits of the PSS peak currents using a hyperbolic equation to derive K_D^{app} and relative I_{max} values for MDG in the presence of different cations. **C** Zoom of the current traces shown in A to show the transport current phase. The MDG concentrations are color coded as indicated in A. **D** Fits of the currents 50 ms after substrate jump as shown in C, using a hyperbolic equation to derive K_M and relative transport I_{max} values for MDG in the presence of different cations.

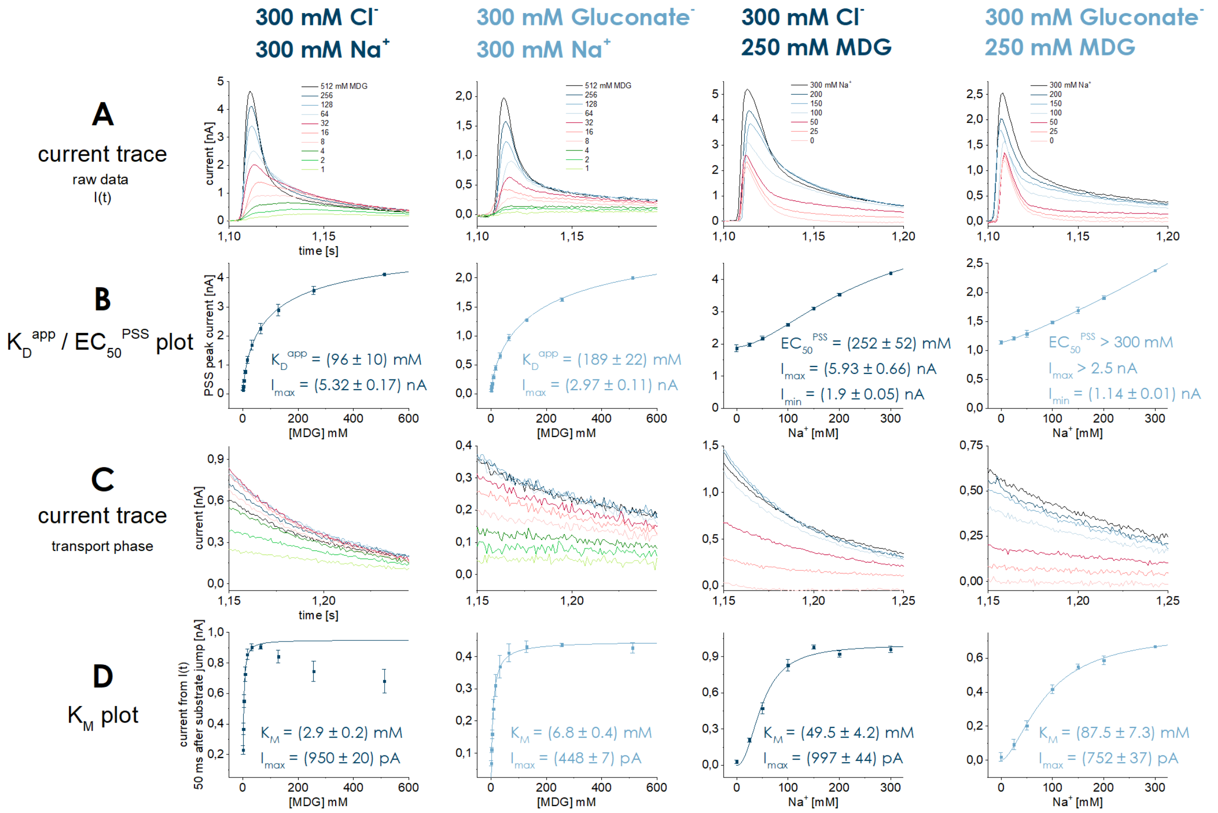

Figure SI-7: Effect of Cl^- on K_M and K_D^{app} / EC_{50}^{PSS} for MDG and Na^+ during Na^+/MDG cotransport. MDG concentration jumps were performed in the presence of either 300 mM Cl^- or 300 mM gluconate in all measurement solutions. To determine K_M and K_D for MDG, MDG concentration jumps were performed in the presence of 300 mM Na^+ as shown in Figure 1 for D-glucose. To determine K_M and EC_{50}^{PSS} for Na^+, 250 mM MDG concentration jumps were performed in the presence of different Na^+ concentrations as shown in Figure 3 for D-glucose. Data was averaged and normalized across at least 4 different sensors as described in Figure SI-3. **A** Representative current traces recorded on a 1 mm sensor for different MDG or different Na^+ concentrations in the presence or absence of Cl^-. Na^+ and MDG concentrations are indicated. **B** Fits of the PSS peak currents using a hyperbolic equation to derive K_D^{app} for MDG or EC_{50}^{PSS} for Na^+ and relative I_{max} values in the presence or absence of Cl^-. **C** Zoom of the current traces shown in A to show the transport current phase. The MDG and Na^+ concentrations are color coded as indicated in A. **D** Fits of the currents 50 ms after substrate concentration jump as shown in C, using a hyperbolic equation to derive K_M and relative transport I_{max} values for MDG and Na^+ in the presence or absence of Cl^-.

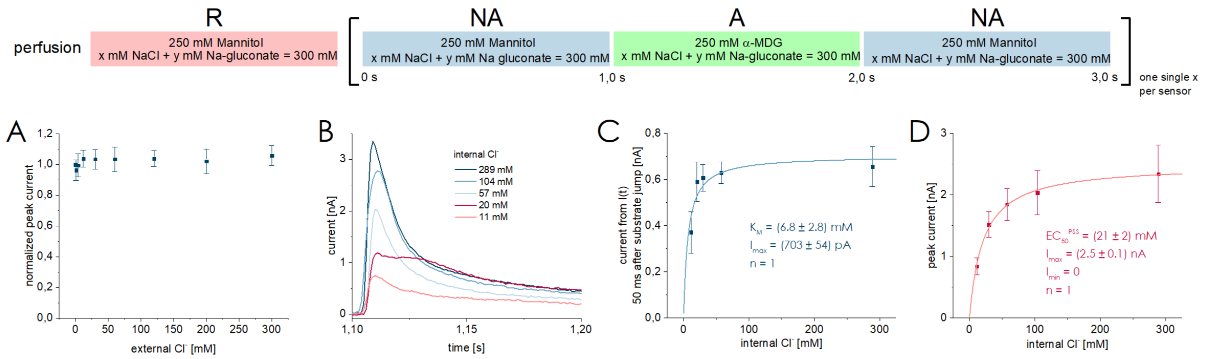

Figure SI-8: K_M and EC_{50}^{PSS} for Cl^- during Na^+/MDG cotransport. All current traces were recorded in the presence of different Cl^- concentrations upon 250 mM MDG concentration jumps. **A** Before sensor preparation, the internal composition of the vesicles was equilibrated at 0 mM chloride. Cl^- concentration was varied during SSME measurements by changing the composition of the external (NA,A) solutions on the same sensor for n=6 different sensors. Peak currents were normalized to the data point at 0 mM Cl^-, followed by averaging across sensors. No effects of external Cl^- on MDG-induced peak currents are observed. **B** Representative current traces at different internal Cl^- concentrations. Each current trace is recorded from a different sensor with vesicles equilibrated at different Cl^- concentrations before sensor preparation. Since the vesicles storage buffer contained 150 mM Cl^-, the minimum internal Cl^- concentration is limited by the dilution ratio with the respective measurement solutions containing 0 – 300 mM Cl^-. The corrected internal Cl^- concentration range used was 11.25 mM to 289 mM as indicated. **C** Currents 50 ms after substrate concentration jump are fitted using a hyperbolic equation to derive a K_M of 6.8 ± 2.8 mM for Cl^-. Average currents from n=3 different sensors for each internal Cl^- concentration were used. No normalization was applied, since individual data points were derived from different sensors. **D** Same as C, but peak currents are fitted to derive an EC_{50}^{PSS} of 21 ± 2 mM for Cl^-. In order to improve the fit, we fixed n = 1 and I_{min} = 0.

S4. Supplementary Tables

		Label	Parameter	D-glucose	α-MDG	D-galactose	3-OMG	D-xylose
Sugar-induced currents in the presence of 300 mM Na^+	Transport		$I_{max,rec}$ [pA] - raw data	782 ± 23	941 ± 17	1630 ± 60	2080 ± 74	671 ± 160
		**A**	**$I_{max,rec}$ [pA] – norm.**	**698 ± 20**	**1074 ± 19**	**961 ± 35**	**1338 ± 47**	**902 ± 215**
			$I_{max,t=50ms}$ [pA] - raw data	360 ± 12	439 ± 9	632 ± 11	765 ± 35	318 ± 48
			$I_{max,t=50ms}$ [pA] – norm.	308 ± 10	592 ± 8	508 ± 9	669 ± 31	436 ± 65
			$Q_{max,0\text{-}1.0s}$ [pC] - raw data	65.3 ± 1.2	90 ± 2	139 ± 3	130 ± 7	139 ± 79
		B	$Q_{max,0\text{-}1.0s}$ [pC] – norm.	65.7 ± 1.2	101 ± 2	97 ± 2	110 ± 6	129 ± 74
		**C**	**K_M (I_{rec}) [mM]**	**1.92 ± 0.21**	**4.1 ± 0.3**	**10.7 ± 1.1**	**241 ± 18**	**305 ± 167**
			K_M ($I_{t=50ms}$) [mM]	1.05 ± 0.08	1.95 ± 0.1	3.8 ± 0.22	68 ± 8	152 ± 57
			K_M ($Q_{0\text{-}1.0}$) [mM]	1.63 ± 0.13	4.8 ± 0.4	18.2 ± 1.6	89 ± 14	> 500
		D	k_{obs} [s^{-1}]	10.3 ± 0.3	10 ± 0.5	11.6 ± 0.7	8 ± 1.3	7.4 ± 0.8
	Pre-steady-state		I_{max} [nA] - raw data	2.56 ± 0.09	2.48 ± 0.08	2.86 ± 0.13	2.57 ± 0.47	> 0.8
		E	I_{max} [nA] – norm.	3.06 ± 0.1	2.39 ± 0.07	2.39 ± 0.11	2.47 ± 0.28	> 0.7
			$Q_{max,0\text{-}0.03s}$ [pC] - raw data	29.9 ± 0.7	30.4 ± 0.5	40.8 ± 1	34.3 ± 7	> 14 pC
		**F**	**$Q_{max,0\text{-}0.03s}$ [pC] – norm.**	**34 ± 0.1**	**40.3 ± 0.6**	**39.2 ± 1**	**45.6 ± 9**	**> 15 pC**
		**G**	**$K_{D,Na}^{app}$ (I_{peak}) [mM]**	**14.4 ± 1.6**	**32 ± 3**	**49 ± 7**	**337 ± 105**	**>> 500**
			$K_{D,Na}^{app}$ ($I_{rec,peak}$) [mM]	15.9 ± 1.1	32 ± 1	53 ± 5	331 ± 119	>> 500
			$K_{D,Na}^{app}$ ($Q_{0\text{-}0.03s}$) [mM]	8.6 ± 0.7	28.7 ± 1.3	54 ± 4	371 ± 131	>> 500
			$K_{D,Na}^{app}$ (k_{obs}) [mM]	26.5 ± 3.4	78.2 ± 25.4	82 ± 62	172 ± 8	N.A.
		H	k_{on} [s^{-1}]	208 ± 3	187 ± 17	145 ± 17	95 ± 5	N.A.
			k_{off} [s^{-1}]	56 ± 4	~0	~0	2 ± 1	N.A.
Sugar-induced currents in the absence of Na^+	Pre-steady-state		I_{max} [nA] - raw data	1.85 ± 0.51	1.2 ± 0.19	>> 0.5	>> 0.3	>> 0.3
		I	I_{max} [nA] – norm.	2.27 ± 0.62	1.51 ± 0.24	>> 0.7	>> 0.25	>> 0.25
			Q_{max} [pC] - raw data	20 ± 1.8	23.3 ± 2.6	23.6 ± 3.1	26.4 ± 13.4	> 9 pC
		**J**	**Q_{max} [pC] – norm.**	**15 ± 1.4**	**21.3 ± 2.4**	**18.3 ± 2.4**	**19.2 ± 9.7**	**> 6 pC**
		K	k_{obs} [s^{-1}]	94 ± 7	74 ± 5	84 ± 4	36 ± 1	37 ± 8
			$K_{D,K}^{app}$ (I) [mM]	507 ± 383	233 ± 86	>> 500	>> 500	>> 500
		**L**	**$K_{D,K}^{app}$ (Q) [mM]**	**210 ± 52**	**234 ± 64**	**851 ± 262**	**734 ± 534**	**>> 500**
Derived constants		M	$I_{max,rec}/K_M$ [pA/mM]	364	262	90	5.6	3.0
		N	$I_{max,rec}/K_{D,Na}$ [pA/mM]	48.5	33.6	19.6	4.0	< 1.8
		O	$K_{D,K}/K_{D,Na}$	14.6	7.3	17.4	2.2	N.A.
		P	$K_{D,Na}/K_M$	7.5	7.8	4.6	1.4	> 1.6
		Q	$K_{D,K}/K_M$	109	57	80	3.0	> 1.6

Table SI-1: **Kinetic parameters obtained from transport and PSS currents using different sugar substrates in the presence and absence of Na^+.** Full version of Table 1 found in the main manuscript. Rows A-Q are identical with table 1. This table contains two additional sets of information: First, parameters labeled as 'raw data' refer to I_{max} or Q_{max} values obtained from different sets of sensors for each sugar species as found from the raw data provided in Figure SI-3, SI-4 and SI-5. There was no normalization to reflect proper relative currents or charges for different sugars. Parameters labeled as 'norm.' reflect those provided in the main manuscript. Norm. values were recalculated using the raw data values by normalization to the datasets shown in Figure 6. Here, all sugar species were recorded on the same sensor, hence parameters may be compared directly. For comparison of I_{max} and Q_{max} across sugar substrates, only the normalized values are used. Second, K_D^{app} and K_M values obtained from different analysis methods are shown to provide insights about how parameters compare, when different read-outs are used. To obtain K_M values three methods are compared: we used the steady-state current from current reconstruction (K_M (I_{rec})), the current 50 ms after substrate jump (K_M ($I_{t=50ms}$)) and the current integral (K_M ($Q_{0\text{-}1.0}$)). To determine the sugar affinity for the Na^+ bound carrier ($K_{D,Na}^{app}$), we have used the peak currents from the raw data ($K_{D,Na}^{app}$ (I_{peak})), the peak currents from current reconstruction ($K_{D,Na}^{app}$ ($I_{rec,peak}$)), the current integral within the first 30 ms after substrate jump ($K_{D,Na}^{app}$ ($Q_{0\text{-}0.03s}$)) and a model equation fit based on the k_{obs} values determined via bi-exponential fit of the current decays ($K_{D,Na}^{app}$ (k_{obs})). To determine the sugar affinity for the empty carrier ($K_{D,K}^{app}$), we either used the peak currents ($K_{D,K}^{app}$ (I)) or the current integral ($K_{D,K}^{app}$ (Q)).

parameter	conditions	Experimental output	Model output
**Steady-state parameters**			
$K_M(Na)$	Sugar: 250 mM	39 ± 4 mM (Figure 3B),<br>59 ± 13 mM (Figure 3B)	31 mM
$K_M(S)$	Na: 300 mM	0.8 ± 0.2 mM (Figure 5C),<br>1.92 ± 0.21 mM (Figure 1G)	2 mM
Na gradient effect on transport I_{max}	Na_o: 300 mM, Na_i: 0 mM, Sugar: 250 mM	x (3.48 ± 0.58) (Figure 7A, inset)	x 2.06
$K_M(S)^{100\ mM\ Na}/K_M(S)^{300\ mM\ Na}$		2.9 ± 0.8 (Figure 5C)	3.5
$K_M(S)^{50\ mM\ Na}/K_M(S)^{300\ mM\ Na}$		9.4 ± 6 (Figure 5C)	9.9
$K_M(S)^{20\ mM\ Na}/K_M(S)^{300\ mM\ Na}$		55 ± 39 (Figure 5C)	34
$K_M(Na)^{20\ mM\ S}/K_M(Na)^{250\ mM\ S}$		1.3 ± 0.1 (Figure 5D)	1.8
$K_M(Na)^{4\ mM\ S}/K_M(Na)^{250\ mM\ S}$		2.3 ± 0.3 (Figure 5D)	2.5
$K_M(Na)^{1\ mM\ S}/K_M(Na)^{250\ mM\ S}$		3.6 ± 0.9 (Figure 5D)	2.9
$K_M(S)$ in efflux mode	Na: 300 mM	2.2 ± 1.7 (Figure SI-1)	5.5 mM
**Pre-steady-state parameters: 4 ↔ 5<br>Sugar binding to Na^+ bound carrier**			
k_{obs}	Na: 300 mM, Sugar: 250 mM	244 ± 2 s^{-1} (Figure 4)	191 s^{-1}
k_{obs}	Na: 300 mM, Sugar: 8 mM	103 ± 5 s^{-1} (Figure 4)	92 s^{-1}
$EC_{50}(S)$ from current fit	Na: 300 mM	14.5 ± 1.6 mM (Figure 1H),<br>38 ± 6 mM (Figure 5C)	20 mM
$EC_{50}(S)$ from model equation fit	Na: 300 mM	26.5 ± 3.4 mM (Figure 4C)	27 mM
$EC_{50}(Na)$ from current	Sugar: 250 mM	231 ± 12 mM (Figure 5D),<br>285 ± 39 mM (Figure 3H)	102 mM
Na gradient effect on PSS I_{max}	Na_o: 300 mM, Na_i: 0 mM, Sugar: 250 mM	x (1.36 ± 0.12) (Figure 7A, inset)	x 1.17
PSS / SS ratio	Na: 300 mM, Sugar: 250 mM	4.38 ± 0.27 (Table 1)	15
Peak current ratio influx / efflux	Na: 300 mM, Sugar: 250 mM	7.2 ± 2.4 (Figure SI-1)	2.74
**Pre-steady-state parameters: A ↔ B<br>Sugar binding to empty carrier**			
k_{obs}	Na: 0 mM, Sugar: 500 mM	95 ± 18 s^{-1} (Figure 2)	53 s^{-1}
k_{obs}	Na: 0 mM, Sugar: 15 mM	85 ± 17 s^{-1} (Figure 2)	44 s^{-1}
$EC_{50}(S)$ from charge translocation	Na: 0 mM	210 ± 52 mM (Figure 2E)	182 mM
**Pre-steady-state parameters: C ↔ 5<br>Na^+ binding to sugar bound carrier**			
k_{obs}	Na: 60 mM, Sugar: 250 mM	139 ± 20 s^{-1} (Bazzone et al., 2022a)	167 s^{-1}

Table SI-2: Comparison of experimentally determined kinetic parameters with those obtained from model simulations under the same conditions. When more than one experimental output was obtained due to multiple sets of experiments or different approaches to fit the data, highest and lowest obtained values are given. The data obtained from model simulations are based on the set of rate constants shown in Table SI-3, plus a time filter of 2 ms which simulates the time resolution threshold for the solution exchange in SSME experiments (Bazzone et al., 2017a).

parameter	value	process	origin of the value
**substrate binding pathway - outward facing: Na⁺ first**			
k_{12}	500.000 $M^{-2}s^{-1}$	Na^+ binding to empty carrier	Na^+ binding kinetics was taken from (Loo et al., 2006) and slightly adjusted to match experimental K_D of 20 mM from (Loo et al., 2013).
k_{21}	10.000 s^{-1}		
k_{23}	250 s^{-1}	Non-electrogenic transition, e.g. opening of the extracellular gate	Not accessible via experiment, since not electrogenic (Bazzone et al., 2022a). Added for symmetry purposes and adjusted to match experimental results
k_{32}	35 s^{-1}		
k_{34}	40.000 $M^{-1}s^{-1}$	Sugar binding to Na^+ bound carrier	k_{on} taken from (Loo et al., 2006) - k_{off} set to match K_D^{app}= 26 mM from data fit in Figure 4.
k_{43}	1040 s^{-1}		
k_{45}	200 s^{-1}	Electrogenic PSS upon sugar binding	From data fit in Figure 4.
k_{54}	60 s^{-1}		
**substrate binding pathway - outward facing: Sugar first**			
k_{1A}	40.000 $M^{-1}s^{-1}$	Sugar binding to empty carrier	k_{on} is assumed to be the same as for sugar binding in the Na^+ first pathway. k_{off} is set to match K_D = 200 mM from data fit in Figure 2.
k_{A1}	8.000 s^{-1}		
k_{AB}	100 s^{-1}	Electrogenic PSS upon sugar binding	k_{on} is required to simulate k_{obs} of PSS current in absence of Na^+ (Figure 2). k_{off} is adjusted to match experimental results
k_{BA}	57 s^{-1}		
k_{BC}	500.000 $M^{-2}s^{-1}$	Na^+ binding to sugar bound carrier	Na^+ binding kinetics is assumed to be the same as in the Na^+ first pathway.
k_{CB}	10.000 s^{-1}		
k_{C5}	522 s^{-1}	Electrogenic PSS upon Na^+ binding	Required to simulate k_{obs} for Na^+ induced PSS in the presence of sugar as found in (Bazzone et al., 2022a). k_{on} was set according to detailed balance
k_{5C}	5 s^{-1}		
**alternating access and substrate release**			
k_{56}	50 s^{-1}	Alternating access of substrate bound carrier	k_{on} was taken from (Loo et al., 2006) and k_{off} slightly adjusted to match experimental results. Rate limiting step for D-glucose translocation, likely increased for minor substrates with lower apparent affinity.
k_{65}	135 s^{-1}		
k_{67}	480 s^{-1}	Sugar release	Similar as reported in (Eskandari et al., 2005) (here: k_{on} = 444k $M^{-1}s^{-1}$ and k_{off} = 800 s^{-1}). Sugar binding rate is set via detailed balance; rates match a K_D of ~5 mM.
k_{76}	91.119 $M^{-1}s^{-1}$		
k_{78}	500 s^{-1}	Na^+ release	In contrast to (Loo et al., 2006), Na^+ release is assumed to be faster than 5 s^{-1}, similar as in (Longpré et al., 2012) who merged all release steps at a rate of 300 s^{-1}. The rates match with a K_D of 50 mM. V_{max} dependence on internal Na^+ concentration is a result of the velocity of the subsequent empty carrier translocation.
k_{87}	10.000 $M^{-2}s^{-1}$		
k_{81}	15 s^{-1}	Alternating access of empty carrier	This step is subdivided in multiple voltage dependent steps by (Loo et al., 2006) and (Longpré et al., 2012) with the slowest rate being 5 s^{-1} and 60 s^{-1}, respectively. We assume k_{on} being rate limiting, required to match results in the presence of a Na^+ gradient (Figure 7A). k_{off} is set according to match experimental results.
k_{18}	67 s^{-1}		

Table SI-3: Parameters for the 11-state kinetic model describing Na^+/D-glucose cotransport. Rate constants used to simulate the kinetic model shown in Figure 8 are listed. The last column explains the reasoning behind the value. Rate constants for the substrate induced conformational transitions recorded as PSS currents in SSME and K_D values for sugar binding were experimentally determined. They were combined with information from the literature as explained. Some rate constants are modified to account for the law of detailed balance (Alberty, 2004), others are modified to match the experimental data.

References

Adelman, J. L., Ghezzi, C., Bisignano, P., Loo, D. D. F., Choe, S., Abramson, J. et al. (2016). Stochastic steps in secondary active sugar transport. *Proceedings of the National Academy of Sciences of the United States of America* 113, E3960-6. doi: 10.1073/pnas.1525378113.

Alberty, R. A. (2004). Principle of Detailed Balance in Kinetics. *J. Chem. Educ.* 81, 1206. doi: 10.1021/ed081p1206.

Bazzone, A., and Barthmes, M. (2020). Functional Characterization of SLC Transporters Using Solid Supported Membranes. *Methods in molecular biology (Clifton, N.J.)* 2168, 73–103. doi: 10.1007/978-1-0716-0724-4_4.

Bazzone, A., Barthmes, M., and Fendler, K. (2017a). SSM-Based Electrophysiology for Transporter Research. *Methods in enzymology* 594, 31–83. doi: 10.1016/bs.mie.2017.05.008.

Bazzone, A., Körner, A., Meincke, M., Bhatt, M., Dondapati, S., Barthmes, M. et al. (2022a). SSM-based electrophysiology, a label-free real-time method reveals sugar binding & transport events in SGLT1. *Biosensors & bioelectronics* 197, 113763. doi: 10.1016/j.bios.2021.113763.

Bazzone, A., Madej, M. G., Kaback, H. R., and Fendler, K. (2016). pH Regulation of Electrogenic Sugar/H+ Symport in MFS Sugar Permeases. *PloS one* 11, e0156392. doi: 10.1371/journal.pone.0156392.

Bazzone, A., Tesmer, L., Kurt, D., Kaback, H. R., Fendler, K., and Madej, M. G. (2022b). Investigation of sugar binding kinetics of the E. coli sugar/H+ symporter XylE using solid-supported membrane-based electrophysiology. *The Journal of biological chemistry* 298, 101505. doi: 10.1016/j.jbc.2021.101505.

Bazzone, A., Zabadne, A. J., Salisowski, A., Madej, M. G., and Fendler, K. (2017b). A Loose Relationship: Incomplete H+/Sugar Coupling in the MFS Sugar Transporter GlcP. *Biophysical journal* 113, 2736–2749. doi: 10.1016/j.bpj.2017.09.038.

Eskandari, S., Wright, E. M., and Loo, D. D. F. (2005). Kinetics of the reverse mode of the Na+/glucose cotransporter. *The Journal of membrane biology* 204, 23–32. doi: 10.1007/s00232-005-0743-x.

Garcia-Celma, J. J., Ploch, J., Smirnova, I., Kaback, H. R., and Fendler, K. (2010). Delineating electrogenic reactions during lactose/H+ symport. *Biochemistry* 49, 6115–6121. doi: 10.1021/bi100492p.

Gorraitz, E., Hirayama, B. A., Paz, A., Wright, E. M., and Loo, D. D. F. (2017). Active site voltage clamp fluorometry of the sodium glucose cotransporter hSGLT1. *Proceedings of the National Academy of Sciences of the United States of America* 114, E9980-E9988. doi: 10.1073/pnas.1713899114.

Hirayama, B. A., Loo, D. D. F., Díez-Sampedro, A., Leung, D. W., Meinild, A.-K., Lai-Bing, M. et al. (2007). Sodium-dependent reorganization of the sugar-binding site of SGLT1. *Biochemistry* 46, 13391–13406. doi: 10.1021/bi701562k.

Longpré, J.-P., Sasseville, L. J., and Lapointe, J.-Y. (2012). Simulated annealing reveals the kinetic activity of SGLT1, a member of the LeuT structural family. *The Journal of general physiology* 140, 361–374. doi: 10.1085/jgp.201210822.

Loo, D. D., Eskandari, S., Boorer, K. J., Sarkar, H. K., and Wright, E. M. (2000). Role of Cl- in electrogenic Na+-coupled cotransporters GAT1 and SGLT1. *The Journal of biological chemistry* 275, 37414–37422. doi: 10.1074/jbc.M007241200.

Loo, D. D. F., Hirayama, B. A., Karakossian, M. H., Meinild, A.-K., and Wright, E. M. (2006). Conformational dynamics of hSGLT1 during Na+/glucose cotransport. *The Journal of general physiology* 128, 701–720. doi: 10.1085/jgp.200609643.

Loo, D. D. F., Jiang, X., Gorraitz, E., Hirayama, B. A., and Wright, E. M. (2013). Functional identification and characterization of sodium binding sites in Na symporters. *Proceedings of the National Academy of Sciences of the United States of America* 110, E4557-66. doi: 10.1073/pnas.1319218110.

Mikušević, V., Schrecker, M., Kolesova, N., Patiño-Ruiz, M., Fendler, K., and Hänelt, I. (2019). A channel profile report of the unusual K+ channel KtrB. *The Journal of general physiology* 151, 1357–1368. doi: 10.1085/jgp.201912384.

Peerce, B. E., and Wright, E. M. (1984). Conformational changes in the intestinal brush border sodium-glucose cotransporter labeled with fluorescein isothiocyanate. *Proceedings of the National Academy of Sciences of the United States of America* 81, 2223–2226. doi: 10.1073/pnas.81.7.2223.

Quick, M., Tomasevic, J., and Wright, E. M. (2003). Functional asymmetry of the human Na+/glucose transporter (hSGLT1) in bacterial membrane vesicles. *Biochemistry* 42, 9147–9152. doi: 10.1021/bi034842x.

Sala-Rabanal, M., Hirayama, B. A., Loo, D. D. F., Chaptal, V., Abramson, J., and Wright, E. M. (2012). Bridging the gap between structure and kinetics of human SGLT1. *American journal of physiology. Cell physiology* 302, C1293-305. doi: 10.1152/ajpcell.00397.2011.

Sauer, G. A., Nagel, G., Koepsell, H., Bamberg, E., and Hartung, K. (2000). Voltage and substrate dependence of the inverse transport mode of the rabbit Na + /glucose cotransporter (SGLT1). *FEBS Letters* 469, 98–100. doi: 10.1016/s0014-5793(00)01255-2.

Tadini-Buoninsegni, F., and Fendler, K. (2015). "Recording of Pump and Transporter Activity Using Solid-Supported Membranes (SSM-Based Electrophysiology)," in *Pumps, Channels, and Transporters*, ed. R. J. Clarke and M. A. A. Khalid (Hoboken, NJ: John Wiley & Sons, Inc), 147–177.
